# Interrelations of Sphingolipid and Lysophosphatidate Signaling with Immune System in Ovarian Cancer

**DOI:** 10.1016/j.csbj.2019.04.004

**Published:** 2019-04-10

**Authors:** Anastasia Meshcheryakova, Martin Svoboda, Markus Jaritz, Felicitas Mungenast, Martina Salzmann, Dietmar Pils, Dan Cacsire Castillo-Tong, Gudrun Hager, Andrea Wolf, Elena Ioana Braicu, Jalid Sehouli, Sandrina Lambrechts, Ignace Vergote, Sven Mahner, Peter Birner, Philip Zimmermann, David N. Brindley, Georg Heinze, Robert Zeillinger, Diana Mechtcheriakova

**Affiliations:** a Molecular Systems Biology and Pathophysiology Research Group, Department of Pathophysiology and Allergy Research, Center for Pathophysiology, Infectiology and Immunology, Medical University of Vienna, Vienna, Austria; b Research Institute of Molecular Pathology, Vienna Biocenter, Vienna, Austria; c Sectionfor Clinical Biometrics, Center for Medical Statistics, Informatics, and Intelligent Systems, Medical University of Vienna, Vienna, Austria; d Translational Gynecology Group, Department of Obstetrics and Gynecology, Comprehensive Cancer Center, Medical University of Vienna, Vienna, Austria; e Molecular Oncology Group, Department of Obstetrics and Gynecology and Comprehensive Cancer Center, Gynecologic Cancer Unit, Medical University of Vienna, Vienna, Austria; f Charité – Universitätsmedizin Berlin, Humboldt-Universität zu Berlin, Berlin Institute of Health, Department of Gynecology, Berlin, Germany; g Division of Gynecologic Oncology, University Hospital Leuven, Leuven Cancer Institute, KU Leuven, Leuven, Belgium; h Department of Gynecology and Gynecologic Oncology, University Medical Center Hamburg-Eppendorf, Hamburg, Germany; i Department of Pathology, Medical University of Vienna, Vienna, Austria; j Nebion AG, Zürich, Switzerland; k Cancer Research Institute of Northern Alberta, Department of Biochemistry, University of Alberta, Edmonton, Alberta, Canada

**Keywords:** Sphingolipid/lysophosphatidate system, On-site immune response, Patient-specific expression data sets, Integrative analysis algorithm, Patient stratification, From systems biology to systems medicine

## Abstract

The sphingolipid and lysophosphatidate regulatory networks impact diverse mechanisms attributed to cancer cells and the tumor immune microenvironment. Deciphering the complexity demands implementation of a holistic approach combined with higher-resolution techniques. We implemented a multi-modular integrative approach consolidating the latest accomplishments in gene expression profiling, prognostic/predictive modeling, next generation digital pathology, and systems biology for epithelial ovarian cancer. We assessed patient-specific transcriptional profiles using the sphingolipid/lysophosphatidate/immune-associated signature. This revealed novel sphingolipid/lysophosphatidate-immune gene-gene associations and distinguished tumor subtypes with immune high/low context. These were characterized by robust differences in sphingolipid‐/lysophosphatidate-related checkpoints and the drug response. The analysis also nominates novel survival models for stratification of patients with *CD68*, *LPAR3*, *SMPD1*, *PPAP2B*, and *SMPD2* emerging as the most prognostically important genes. Alignment of proprietary data with curated transcriptomic data from public databases across a variety of malignancies (over 600 categories; over 21,000 arrays) showed specificity for ovarian carcinoma. Our systems approach identified novel sphingolipid-lysophosphatidate-immune checkpoints and networks underlying tumor immune heterogeneity and disease outcomes. This holds great promise for delivering novel stratifying and targeting strategies.

## Graphical Abstract

**Figure:**
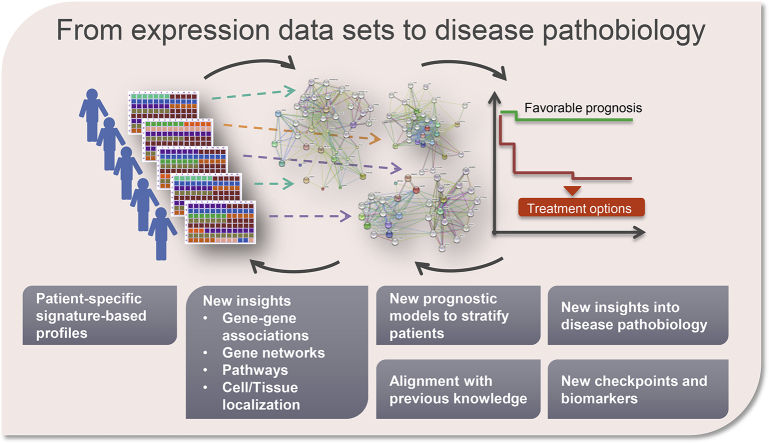

## 1 Introduction

### 1.1 Deciphering the Complexity of Ovarian Cancer in a Patient-orientated Manner

Serous ovarian cancer is one of the leading cancer types underlying cancer-related deaths in women. It is mostly diagnosed at an advanced stage and shows a relapse rate of about 70% [1,2]. The major clinical challenge of high-grade serous ovarian cancer is an ultimate development of progressive resistance to chemotherapy in the majority of patients [3]. A further distinctive cornerstone is the high inter- and intra-tumoral genetic, inflammatory, and immune heterogeneity [[4], [5], [6], [7], [8], [9], [10]]. There is a lack of clear molecular criteria to stratify (group) the patients for effective application of molecular-targeted agents, including immunotherapeutic interventions. Advanced techniques such as nanoString, CIBERSORT and Immunoscore are capable of stratifying patients according to molecular- or immune-related criteria and subsequently predicting disease outcome [[11], [12], [13]]. These techniques have provided unprecedented perspectives in decoding heterogeneity of several tumor types. Unfortunately, there is no breakthrough in understanding of serous ovarian cancer. Thus, for this aggressive type of cancer, there is a particular need to continue searching for new pathways/ targets for therapeutic modulation and new effective strategies to stratify patients to risk groups. We will provide a novel approach to this challenge based on the understanding of the dysregulation of the sphingolipid signaling system that occurs in cancer, its crosstalk with the lysophosphatidate system, and the interrelation with the local tumor immune microenvironment.

### 1.2 The Complexity of Sphingolipid System and the Sphingolipid-related Checkpoints

The sphingolipid machinery consists of a complex interconnected network of bioactive sphingolipid mediators, the enzymes responsible for their syntheses, degradation or turnover, and the family of five sphingosine 1-phosphate (S1P)-specific G-protein-coupled receptors (S1PR1-5). The system is crucially involved in the regulation of diverse biological processes. These involve the decision-making for cell proliferation versus differentiation, cell survival versus apoptosis, cell residence versus migration, as well as of angiogenic response and immune response/immunomodulation [[14], [15], [16], [17], [18]]. Aberrations/dysregulations of the mechanisms underlying these processes are linked to several pathologies. Summing-up the current knowledge on the (patho)biology of the sphingolipid signaling machinery encouraged us to introduce the concept of “sphingolipid-related checkpoints” – similarly to “cell cycle checkpoints” and “immune checkpoints”. These checkpoints are critical for therapeutic interventions [19].

### 1.3 The Sphingolipid/Lysophosphatidate-related Checkpoints as Biomarkers and/or Targets in Cancer

Extensive research over the last two decades identifies a fundamental and multistep involvement of the sphingolipid machinery in a variety of aspects of neoplastic cell behavior that is critical for tumor development and progression [16,[20], [21], [22]]. The below list of examples emphasizes the ultimate impact of the sphingolipid system in cancer pathobiology and gives the rationale to consider sphingolipid-related checkpoints as attractive targets for therapeutic intervention. This furthermore points out the necessity of taking into account the interconnections between the sphingolipid and lysophosphatidate (also known as lysophosphatidic acid, LPA) signaling systems for harnessing the power of potential complementary targeting strategies. (I) *SPHK1* – gene encoding a lipid kinase that phosphorylates sphingosine to S1P – acts as an oncogene ([23] and as reviewed in [16,20]). SPHK1 is involved in the development of drug resistance for cancer treatments and acts as a chemotherapy sensor [[24], [25], [26], [27]]. (II) S1P gradient and S1P/S1P receptor axis represents an obligatory signal for trafficking of immune cells [28]. Although the role of LPA is largely unexplored in immune system, recent studies identified its role in transdifferentiation of leukocytes within the monocyte/macrophage lineage [29]. (III) S1P acts on endothelial cells as a strong pro-angiogenic factor and affects tumor angiogenesis [14,30] as well as lymphangiogenesis [31,32]. (IV) S1P and LPA, the related bioactive lipid, in part share the lipid enzymatic machinery, and a cell simultaneously expresses multiple receptors for S1P and LPA [[33], [34], [35]]. (V) The S1P outward-facing gradient (as well as the LPA gradient) created by tumor and/or stroma cells provides the strongest driving factor for cancer cell invasion [36,37]. (VI) S1P and LPA participate in the pro-inflammatory cycle that promotes an inflammatory environment within tumor tissue [33,[38], [39], [40], [41]]. This can contribute to cancer cell evasion of surveillance by the local immune system. (VII) There is a cross-talk between the transactivation of S1PRs and LPARs with critical cancer-attributed receptor tyrosine kinases, including EGFR and PDGFR, and the estrogen receptor [[42], [43], [44], [45], [46]]. (VIII) Ceramides are mediators of cellular stress and, in contrast to pro-survival S1P, ceramides are important regulators of apoptosis (reviewed in [47]). There are multiple enzymes utilizing ceramide as a substrate or producing ceramides, which means that there are numerous agents that can modulate ceramide levels within cancer cells including cytokines, radiation, and chemotherapeutics. Thus, cellular S1P/ceramide rheostat is a decisive checkpoint. (IX) We reported the multidimensional contribution of the sphingolipid machinery to the mechanisms underlying the pathological epithelial to mesenchymal transition (EMT) program during metastasis [33]. (X) The sphingolipid system is druggable at multiple checkpoints (reviewed in [20,48]). Furthermore, several drugs targeting LPA signaling are in clinical trials [39].

### 1.4 Multi-modular Analysis Algorithm for Dissecting the On-site Interrelations between the Sphingolipid and Immune System in Ovarian Cancer

**Fig. 1:**
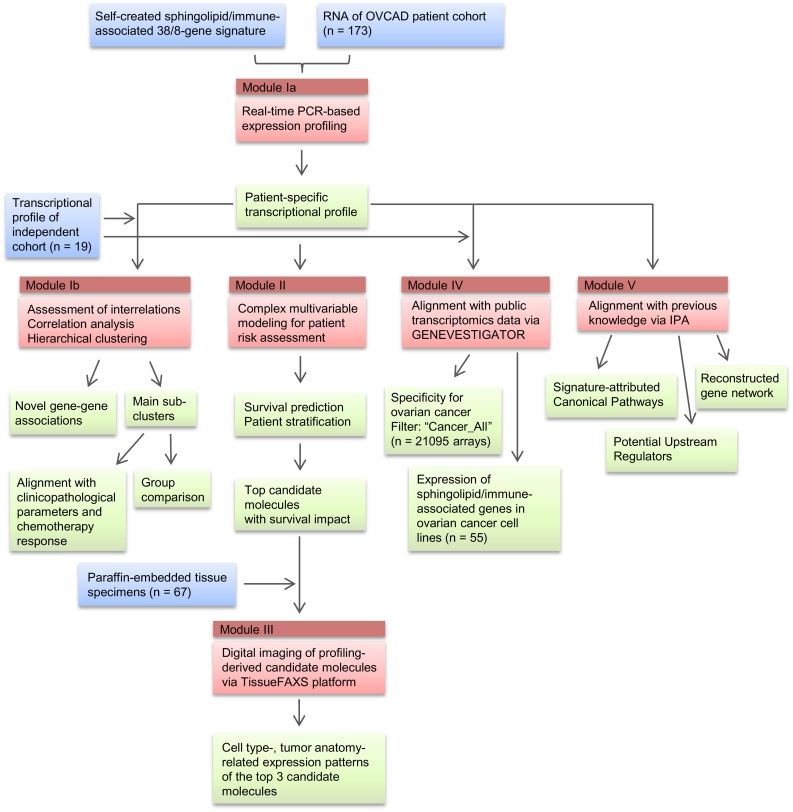
Overview of the study design. Experimental and analytical steps composing the herein used 5-module MuSiCO algorithm are shown. Color code: *blue*, input; *red*, individual modules showing the method/analysis strategy or bioinformatics pipeline; *green*, output. (For interpretation of the references to color in this figure legend, the reader is referred to the web version of this article.)

Despite intense research and considerable progress, there are many gaps in our understanding of the cancer type-specific checkpoints within the sphingolipid machinery and their contribution to the establishment of local immune response within the tumor immune microenvironment. As we previously proposed, the complexity of the sphingolipid signaling system multiplied by the heterogeneity of tumor and the tumor immune microenvironment necessitates the implementation of integrative, systems biology-based approaches for analysis and for obtaining a comprehensive picture. We recently developed such an approach to uncover novel interrelations between the sphingolipid machinery and the EMT program in lung cancer [33]. Here, we performed an integrative analysis to address the impact of sphingolipid and LPA systems to the pathobiology of ovarian cancer in conjunction with immune responses. We further refined the MuSiCO algorithm (from Multigene Signature to the Patient-Orientated Clinical Outcome), which we established recently [49,50], and achieved a comprehensive integration of a set of transcriptional profiles reflecting the perturbations of particular biological system(s). The upgraded MuSiCO algorithm is based on consolidation of the consecutive analytical modules, allowing us to enrich the gene expression data with meaning and context to get more knowledge and insights: (i) multigene signature-based expression profiling of specimens from a clinically well-characterized cohort of patients with primary ovarian cancer and in-depth analysis of transcriptional profiles; (ii) sophisticated statistical modeling for survival prediction using both the profiling-derived variables and clinical risk factors; the added value of the gene signature-derived variables is estimated by comparison of the models performances on the basis of a defined parameter set; (iii) digital imaging of tissue sections to explore the cell type- and tissue anatomy-attributed localization of profiling-derived nominated top candidate molecules; (iv) alignment of signature-derived profiles with publically available transcriptomic data sets covering pathological/cancer conditions using the GENEVESTIGATOR platform to address the question of singularity or commonality, and (v) delineation of data-driven sphingolipid‐/lysophosphatidate- and immune-associated gene network(s) and pathways by applying bioinformatics tools. In comparison with our previously applied strategy [49], the herein refined version of MuSiCO newly includes the digital imaging-based module and the GENEVESTIGATOR-driven analysis of signature specificity. Fig. 1 gives an overview of the study design.

Our results and new algorithm provide unique knowledge about the complex expression patterns of sphingolipid-related genes as well as relationships between sphingolipid-, lysophasphatidate-, and immune-related genes. This information delivers novel patient-stratification strategies for differentiating between immunologically enriched/*immune high* and immunologically poor/*immune low* tumor types and for predicting the clinical outcomes of survival and drug resistance. The study identifies sphingolipid/lysophosphatidate/immune-associated checkpoints as candidates for development of novel targeting and clinical decision-making strategies. Among those S1P/S1PR4 and ENPP2/LPA/LPAR5,6 appeared as critical axes for immune infiltrates; the role of CD68-/SMPD1-associated molecular events was accentuated in tumor microenvironmental macrophages. Overall, this new integrative strategy bridges systems biology and systems medicine. It can be used to deliver novel patient-orientated therapies for ovarian cancer patients. This includes the attractive approach of combinatorial targeting of sphingolipid and lysophosphatidate/LPA systems that offers the power for improving the efficacy of existing therapies and possibly immunotherapies.

## 2 Materials and Methods

### 2.1 Profile of Study Patients

Tumor samples of the first patient group were collected from patients with epithelial ovarian cancer (EOC), which were recruited in the course of the European Commission's sixth framework program project OVCAD from five European university hospitals (Ovarian Cancer: Diagnosis of a silent killer; grant agreement no. 018698) [51]. The patient cohort included 173 patients with primary EOC; the clinicopathological characteristics were documented by experienced clinicians and are summarized in Table 1. For this retrospective study the sample size was determined by the size of the OVCAD patient cohort. The cohort was partially overlapping with the patient group used by us in the previous study [49]. Patient inclusion criterion comprised the EOC with advanced disease (International Federation of Obstetrics and Gynecology (FIGO) II – IV); the majority of patients had advanced-stage ovarian cancer (FIGO III and IV, 95%), G3 tumors (72%), and tumors of serous histology (88%). *Histology* (serous vs. non-serous), *FIGO stage* (II vs. III vs. IV), and *Grading* (G1 and G2 vs. G3) were included as variables in the multivariable modeling. All patients received standard platinum-based chemotherapy, mostly in the adjuvant setting, including intraperitoneal application, but also in the neoadjuvant setting [51]. For the latter, samples were taken before neoadjuvant treatment option (during laparoscopic staging operation or suboptimal cytoreduction). Thus, the entire cohort is uniform in the sense that all tissue samples were taken before chemotherapy. Patients with recurrence or progressive disease until 6 months after the end of chemotherapy were defined as non-responders. The median follow-up time was 31.0 months (95% CI, 28.0–34.0). There were 50 cases (28.9%) of death related to EOC reported during the follow-up period, designated below as events. The second independent group under investigation included 19 patients with primary EOC; the samples were collected at the Medical University of Vienna. The clinicopathological characteristics are summarized in Table S3. In addition, a panel of paraffin-embedded specimens with EOC was retrieved retrospectively from 67 patients who underwent surgery at the Medical University of Vienna; the clinicopathological characteristics of this group are summarized in Table S9. These specimens were used to characterize the cell type- and tumor anatomy-related expression patterns of the top candidate molecules identified by gene expression profiling.

**Table 1:** Clinicopathological characteristics of patient cohort (*n* = 173) used for gene expression profiling.

Patients (%)	173	(100.0)
Age at diagnosis [years]		
Median (range)	56	
Progression-free survival [months]		
Median (range)	16	(1–48)
Number of recurrencies (%)	99	(57.2)
Overall survival [months]		
Median (range)	25	(1–49)
Number of deaths (%)	50	(28.9)
Histology (%)		
Serous	153	(88.4)
Endometrioid	5	(2.9)
Mucinous	2	(1.2)
Mixed epithelial tumor	6	(3.5)
Undifferentiated carcinoma	7	(4.0)
FIGO (%)		
II	8	(4.6)
III	139	(80.3)
IV	26	(15.0)
Histological Grading (%)		
1	6	(3.5)
2	42	(24.3)
3	125	(72.3)
Peritoneal carcinomatosis		
no	52	(30.1)
yes	121	(69.9)
Residual disease after initial surgery (%)		
None	127	(73.4)
≤ 1 cm	30	(17.3)
> 1 cm	16	(9.2)
Type of chemotherapy (%, 26 missing [15.0%])		
Adjuvant	120	(69.4)
Adjuvant Intraperitoneal	15	(8.7)
Neoadjuvant	12	(6.9)
Response to first-line chemotherapy (%, 1 missing [0.6%])		
Responder	127	(73.4)
Non-Responder	45	(26.0)

### 2.2 Ethical Approval and Consent to Participate

The study was approved in accordance to the requirements of the ethical committees of the individual institutions participating in OVCAD (EK207/2003, ML2524, HEK190504, EK366, EK260). Informed consent for the scientific use of biological material was obtained from all patients in accordance with the requirements of the ethics committees of the institutions involved; the herein participating OVCAD partners include Department of Gynecology, Charité – Universitätsmedizin Berlin (Berlin, Germany), Division of Gynecologic Oncology, University Hospital Leuven, Leuven Cancer Institute, KU Leuven (Leuven, Belgium), Department of Obstetrics and Gynecology, Medical University of Vienna (Vienna, Austria), and Department of Gynecology and Gynecologic Oncology, University Medical Center Hamburg-Eppendorf (Hamburg, Germany). The study with the second patient group (*n* = 19) and with paraffin-embedded specimens was approved by the Ethics Committee of the Medical University of Vienna (EK-Nr. 1101/2013). The informed consent was waived by the institutional review board due to the retrospective nature of the study.

### 2.3 RNA Isolation from Tumor Tissues

Total RNA from tumor tissues of the first patient group (*n* = 173) was isolated using the ABI 1600 nucleic acid prepstation (Applied Biosystems) and RNA concentration, purity and integrity were determined on a Nanodrop ND-1000 (Kisker-Biotech, Steinfurt, Germany) and by agarose gel electrophoresis as described previously [52]. Total RNA from tumor tissues of the second patient group (*n* = 19) was isolated using NucleoSpin TriPrep kit (Macherey-Nagel) according to manufacture procedure. RNA integrity was assessed using Experion system (Bio-Rad); samples showed high RNA Quality Index (> 8.0).

### 2.4 Real-timePCR analysis

Total RNA (0.5 μg) was reverse transcribed using the High Capacity cDNA RT kit (Applied Biosystems) according to the manufacturer's instructions. For accurate normalization of mRNA between ovarian cancer tissue specimens, we selected *ACTB*, *TOP1*, *UBC*, and *YWHAZ* as appropriate housekeeping genes (HKGs), as previously described in detail [49]. Primers for genes composing the sphingolipid/lysophosphatidate/immune-associated multigene signature and for HKGs were designed using Primer Express 3.0 software (Applied Biosystems) and validated using a normal tissue panel (Takara, Clontech Laboratories Inc.) as described [40,53]; primer sequences are summarized in Table S17. Real-time PCR analysis was performed in the 384-well plate format using POWER SYBR Green Master Mix (Applied Biosystems) on ABI 7900HT instrument equipped with SDS 2.3 software (Applied Biosystems). The qPCR Human Reference Total RNA (Clontech Laboratories Inc.) was assigned as calibrator sample. Expression levels of genes of interest were normalized to the geometric mean of the four HKGs and shown relative to the calibrator sample.

### 2.5 The Composition of the Sphingolipid/Lysophosphatidate/Immune-associated 38/8-gene Signature

We created a sphingolipid/lysophosphatidate/immune-associated multigene signature to assess a role of sphingolipid machinery and the lysophosphatidate signaling axis in conjunction with the on-site immune response in ovarian cancer tissue. The latter is represented by immune subsets-related molecules and includes the B-cell, the T-cell and the monocyte/macrophage lineage markers. An approach using a self-created sphingolipid-associated multigene signature was recently applied by us to characterize novel checkpoints in sphingolipid machinery attributed to pathological EMT process in lung cancer [33]. The herein applied sphingolipid/lysophosphatidate/immune-associated 38/8-gene signature covers: (i) the interconnected gene network of the sphingomyelin (SM)/salvage pathway including genes encoding the S1P-modifying enzymes such as two families of phosphatases *SGPP1* and *SGPP2*, and *PPAP2A*/*PPAP2C*/*PPAP2B* (also known as *LPP1*/*LPP2*/*LPP3* and *PLPP1*/*PLPP2*/*PLPP3*, respectively); the S1P-degrading *SGPL1* and the S1P-producing sphingosine kinases *SPHK1* and *SPHK2*; the lysophospholipase D, *ENPP2* (also known as autotaxin); genes encoding the Cer-modifying enzymes including Cer-producing *SMPD1*, *SMPD2*, *SMPD3*, and family of Cer synthases *CERS1*, *CERS2*, *CERS3*, *CERS4*, *CERS5*, and *CERS6*; the ceramidases *ASAH1* and *NAAA* (also known as *ASAHL*); the C1P-producing ceramide kinase *CERK*; SM-producing *SGMS1* and *SGMS2*; transferases utilizing Cer *UGT8* and *UGCG*; (ii) the family of G-protein-coupled S1P receptors, *S1PR1*, *S1PR2*, *S1PR3*, *S1PR4*, and *S1PR5*, and, given a cross-talk between S1P and LPA in respect of some metabolizing enzymes and downstream signaling, the related family of LPA receptors, *LPAR1*, *LPAR2*, *LPAR3*, *LPAR4*, *LPAR5*, and *LPAR6*; (iii) the MHC-like molecules *CD1B* and *CD1D* known to present lipids as antigens [54]; and (iv) immune-related markers of the B-cell lineage, *MS4A1* (also known as *CD20*) and *IGHG1* (IgG mature transcripts) and *IGHM* (IgM mature transcript); the monocyte/macrophage lineage, *CD14*, *CD68* and *CD163*; the T-cell marker *CD3E*; the general immune cell marker *PTPRC* (also known as *CD45*). The signature did not cover genes encoding the enzymatic machinery for *de novo* sphingolipid biosynthesis, ACER family, and genes encoding the enzymes of complex glycosphingolipids biosynthesis. Gene symbol, synonyms, gene name, NCBI accession number, and short functional description of genes composing the signature are provided in Table S1.

### 2.6 Immunostaining on Paraffin-embedded Tissue Sections and Staining Evaluation

To determine the localization and expression patterns of the top candidate molecules within cancer tissue of patients with ovarian carcinoma, paraffin-embedded 4 μm-thick sections were stained and acquired using protocols and microscopy-based technology, established by us previously [55]. Sections underwent routine staining with hematoxylin and eosin (HE) to visualize the tumor anatomy. For CD45 and CD68 the entire patient cohort was used (*n* = 67); representative specimens were included to detect LPAR3 (*n* = 12) and SMPD1 (*n* = 7); detailed characterization of the CD45-based and the CD68-based immunological imprints attributed to ovarian cancer is a part of a separate study (manuscript in preparation). We used tonsil tissue sections for optimization of the staining protocol. Being the secondary lymphoid tissue, the tonsil is composed of diverse populations of immune cells (T cells, B cells, mast cells, macrophages, and dendritic cells) and their subpopulations at various activation stages. The tissue architecture, besides the tonsillar follicles with germinal centers, also includes surrounding surface epithelium, high endothelial vessels, crypts, and connective tissue. Such diverse cellular composition allows typically to have both positive (target protein-expressing cells) and negative cellular controls within the same tissue. The knowledge on subcellular localization of the molecule of interest ensures an additional internal control. For purpose of verification, the expression pattern of a candidate molecule can be easily extracted using the GENEVESTIGATOR platform (https://genevestigator.com/gv/ and 2.8, 2.9, 2.10, 2.11) and matched with the staining outcome. To detect CD45, a common leukocyte antigen, rabbit clonal antibody, clone E19-G (DB Biotech) was used; to detect CD68, mouse monoclonal antibody, clone KP1 (Thermo Scientific), was used. LPAR3 and SMPD1 were detected with rabbit polyclonal antibodies (Proteintech). After the first antibody, the DAKO EnVision+ System, Peroxidase/DAB (DAKO) was applied. Tissue sections were counterstained with hematoxylin for nuclear visualization. The automated microscopy-based tissue analysis system, TissueFAXS (TissueGnostics, Vienna, Austria), was used for the acquisition of entire tissue specimens. For acquisition, the 20×/0.5 objective (EC Plan_NeoFluar, Zeiss) was used. HistoQuest software (TissueGnostics, Vienna, Austria) was used for the export of the representative images.

### 2.7 Statistical Analysis and Data Visualization

Expression profiling-derived values were log2 transformed to reduce the influence of disproportionally high expression values. Missing values were imputed using chained Eqs. [56]. For both the clinicopathological variables and the gene profiling-based variables hazard ratios (HR) and corresponding 95% confidence intervals (CI) were estimated by univariate Cox regression analysis using the IBM SPSS statistical package (version 24.0; SPSS Inc., an IBM company).

Model estimation. The R (R Foundation for Statistical Computing, Vienna, Austria) package glmnet [57] was applied to develop prognostic models using regularized multivariable Cox regression as previously [49]. In brief, we applied two different types of penalties which correct for a possible overfit by shrinking regression coefficients towards zero. The optimal amount of shrinkage was thereby estimated by minimizing the partial deviance in a leave-one-out cross-validation procedure. A ridge penalty (ridge) and the LASSO (L1-norm penalization) shrinkage and selection operator were used, supplying a model including all predictor variables or a model with only those deemed most important to predict the outcome, respectively. Absolute values of standardized regression coefficients (STDBETA or$\;{\hat{\beta }}_{j}^{*}$,) were used for comparing and ranking the variables by their importance in prediction. Standardized coefficients were then transformed into ${\hat{S}}_{36}^{\exp\left({\hat{\beta }}_{j}^{*}\right)}$, the estimated 36 months overall survival probability in a subject whose value of *X_j_* differs from the mean of -*X_j_* by 1 SD, and visually compared to the 36 months overall survival rate ${\hat{S}}_{36}$ for a hypothetical subject with all covariate values at the sample means. Similarly, we fitted logistic regression models with ridge and lasso penalties to evaluate the predictive potential of genes for treatment response.

Model validation. To validate the survival models, an outer leave-one-out cross-validation loop was wrapped around the model development process and yielded cross-validated predictors for each patient. We explicitly applied the leave-one-out strategy of cross-validation instead of dividing the data set only once into a training and test set, because the latter strategy would make the results dependent on the particular random split. Global *p*-values for each model were calculated in SPSS using univariate Cox proportional hazards analyses with the corresponding cross-validated predictors of the model as single covariate. Overall survival (OS) and progression-free survival (PFS) were shown by Kaplan-Meier graphs, stratified by quartiles of the cross-validated linear predictors; group differences were tested using log-rank test. We also assessed the discriminative ability of the model by determining the concordance index (c-index) [58] and its proportion of explained variation (PEV) [59], using the cross-validated predictors. For validation of predictive models with binary outcomes we used 40 times repeated 5-fold cross-validation. The statistical test for added value was adopted from De Bin et al. [60]. As described in detail [49], our approach to performance evaluation is robust against falsely attributing any relevance to gene sets with no predictive value.

Correlation analysis was performed in SPSS using Pearson's correlation for log2 transformed expression data sets. Corresponding *p*-values were corrected by the Bonferroni-Holm method using the R package fdrtool (http://strimmerlab.org/software/fdrtool/). The correlation matrix-based bubble plot and heat map were created using the Spotfire software. The clustering method with Euclidean distance measure and average value as ordering weight was applied for the heat map. Clustering analysis and follow-up graphical representation was performed using Cluster 3.0 and Java TreeView programs. Levene's test was used to assess the equality of variances. Principal Component Analysis (PCA) was performed using Qlucore Omics Explorer software. Group differences were assessed by two-way analysis of variance (ANOVA) and Tukey's post hoc test for groups that showed equal variances and Welch's ANOVA and Games-Howell post hoc test for groups where variances were not equal, Chi-square test for categorical variables; Student's *t*-test for two-group comparisons of continuous variables with equal variances, replaced by Welch t-test in case of unequal variances. Two-sided *p*-values < .05 were considered as statistically significant. We followed the recommendations of the REMARK reporting guidelines [61].

### 2.8 Expression Profile of Genes Composing the Sphingolipids/Immune-associated 38/8-gene Signature in Ovarian Cancer Cell Lines

We used GENEVESTIGATOR(https://genevestigator.com/gv/), a manually curated database and analysis platform for publicly available transcriptomic data sets [62], to extract the expression profiles of genes composing the sphingolipid/lysophosphatidate/immune-associated multigene signature. Data from GENEVESTIGATOR are collected from multiple public repositories and extensively curated by experts to create a harmonized and well described compendium for global analysis. All studies assembled for any kind of analysis are referenced in the GENEVESTIGATOR tools. For analysis, we selected data sets from the Affymetrix Human Genome U133 Plus 2.0 Array platform attributed to ovarian cancer cell lines applying the filter “Cell Lines_Pathological Cell Lines_Neoplastic Cell Lines_Neoplastic Cell Lines of the Reproductive System_Ovary”; this selection included 149 arrays. The expression values (log2 transformed) were exported from GENEVESTIGATOR for statistical analysis.

### 2.9 Exploration of the Expression Profiles of S1PR4 and CD1B across Various Tissues Using GENEVESTIGATOR

We first used the Genevisible tool, being a part of the GENEVESTIGATOR platform (https://genevestigator.com/gv/), to assess the expression profile of *S1PR4* and *CD1B* across various tissues (*n* = 464) and identify the top 10 cell types/tissues showing the strongest expression. Second, using the Anatomy tool in GENEVESTIGATOR, we assessed the specificity of marker expression in hematopoietic/immune system cells versus other cell types applying the filter “Anatomy_Cell Type” which included 10,490 expression data sets; the outcome (top 50) was illustrated by a pie chart.

### 2.10 Identification of Diseased Conditions Showing Similarities to Signature-based Profile Using Curated Public Microarray Data Sets; Specificity of Signature-derived Profile

For the in silico identification of conditions that show similarities in gene expression pattern to the sphingolipid/lysophosphatidate/immune-associated signature, we used the Signature Tool from the GENEVESTIGATOR (https://genevestigator.com/gv/) search engine. For analysis, we selected all curated data sets from the Affymetrix Human Genome U133 Plus 2.0 Array platform covering distinct cancer types; this included 680 different neoplasm categories, based on the ICD-10/ICD-O-3 classification. The filtered category “Cancer_All” thus included 21,095 arrays; conditions with *n* < 15 were excluded. Selection of eligible Affymetrix probes for the genes composing the sphingolipid/lysophosphatidate/immune-associated signature was done automatically by GENEVESTIGATOR; for *CD68* the probe 203507_at was selected manually (as unique probe to CD68 according to GeneAnnot). The expression profile of the sphingolipid/lysophosphatidate/immune-associated gene signature, calculated for each gene as log2 transformed median of the expression values across the entire patient cohort (*n* = 173 for the main patient cohort; *n* = 19 for the independent patient cohort), was aligned using Pearson's correlation against the log2 transformed expression values of microarray data sets from the selected category. The same type of analysis was performed for the low risk and the high risk groups of the main patient cohort. The result shows conditions that are similar to the entered profile; the relative similarity index indicates the degree of their resemblance [33]. The ranking was done on the basis of relative similarity and the top 10 most correlated conditions were selected.

### 2.11 Analysis of Co-expressed Genes Using the Published Microarray-based Data Sets

We used the GENEVESTIGATOR search engine (https://genevestigator.com/gv/) for the *in silico* identification of genes showing co-expression with *CD68* and *SMPD1* across immune cells. For analysis, we selected samples from the Affymetrix Human Genome U133 Plus 2.0 Array platform from the anatomical category “Monocyte”, which includes 248 arrays and from the category “Macrophage”, which includes 541 arrays; for both conditions with *n* < 3 were excluded. The co-expression analysis was performed on the “Samples” level in GENEVESTIGATOR. The lists of the top 200 co-expressed probe sets for each gene of interest were exported for the follow-up analysis (Table S10 and Table S11); ranking was based on the Pearson correlation coefficient. Comparison of the two lists was done using VENNY 2.1 (http://bioinfogp.cnb.csic.es/tools/venny/index.html). Comparison was done on the basis of the corresponding gene names. Generally, the total number of genes included into the analysis might be < 200 as for some genes multiple probes identify the same gene. For non-annotated probes instead of the gene name the probe name was used for the follow-up comparative alignment.

### 2.12 Data-drivenSignature-associated Gene Network Reconstruction

The Ingenuity Pathway Analysis (IPA) tool (https://www.qiagenbioinformatics.com/products/ingenuity-pathway-analysis/) was used to reconstruct the gene network on the basis of the gene set representing the overlap between genes co-expressed with *CD68* and *SMPD1*. Furthermore, the IPA tool was used to assign the genes composing the sphingolipid/lysophosphatidate/immune-associated signature to common biological pathways and upstream regulating molecules and for the data-driven gene network reconstruction. We recently used a similar strategy that is described in detail [33,49]. The IPA Core analysis included the categories Canonical Pathways and Upstream Regulators. The corresponding IPA-derived *p*-value determined the probability that the association between the genes from expression profiling data set and a Canonical Pathway or Upstream Regulator can be explained by chance alone. The top ranking was based on the p-value. The following strategy was used to depict Upstream Regulators to be used for network reconstruction: the IPA Upstream Regulator module predicted 283 significant molecules. The top 10 most significant were selected excluding those of the category biological drug/chemical drug/chemical reagent. We additionally added S1P and LPA as IPA knowledge-based non-gene regulators of sphingolipid machinery.

### 2.13 Data and Code Availability

All data generated or analyzed during this study are included in this published article and its supplementary data. Previously published transcriptomic data sets analyzed during the study are available within GENEVESTIGATOR. R code to reproduce modeling results is available from the authors upon request.

## 3 Results

### 3.1 Assessment of the Patient-specific Transcriptional Profiles Using the Sphingolipid/Lysophosphatidate/Immune-associated 38/8-gene Signature

The starting point of the integrative MuSiCO is the assembling of the multigene signature and its application for real-timePCR-based gene expression profiling of clinically well-characterized patient material as described previously in detail [33,49]. The sphingolipid/lysophosphatidate/immune-associated 38/8-gene signature that we applied includes the families of S1P and LPA receptors, the interconnected gene network of sphingolipid-metabolizing enzymes within the sphingomyelin/salvage pathway and the LPA metabolizing enzymes, genes encoding the MHC-like molecules involved in lipid presentation and a set of immune-related genes. Detailed description of the signature composition is given in the *Materials and Methods* including gene symbol, synonyms, gene name, NCBI accession number, and a short functional description, which are provided in Table S1. We assessed the patient-specific transcriptional profiles of tumor tissues from patients with primary epithelial ovarian cancer, EOC (*n* = 173, clinicopathological characteristics of patient cohort are given in Table 1), using this signature.

**Fig. 2:**
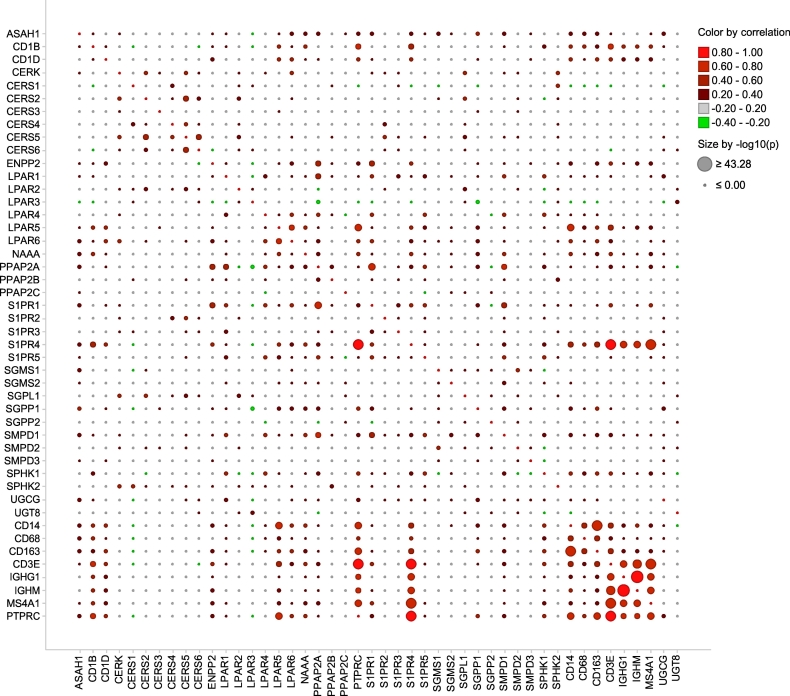
Correlation matrix for sphingolipid/lysophosphatidate/immune-associated genes illustrated by bubble plot. Pearson correlation matrix was calculated across all genes and the entire patient cohort (Table S2). Color indicates the strength and direction of the relationship between two genes based on the corresponding correlation coefficients; color code: *red*, positive correlation; *green*, negative correlation. Dot size is proportional to the *p*-value of correlation upon correction for multiple testing. The bubble plot was created using the Spotfire software. (For interpretation of the references to color in this figure legend, the reader is referred to the web version of this article.)

We utilized the signature-derived expression data sets to examine gene-gene associations. We performed a correlation analysis of profiling-derived variables across the entire patient cohort. Graphical representation of the correlation matrix (Table S2) by bubble plot illustrates both the correlation coefficients and the corresponding *p*-values (Fig. 2). We found multiple strong correlations (*r* > 0.5; *p* < .001) within the set of sphingolipid-associated genes, within the set of immune-related genes, and between genes composing the two sets. Similarity in expression patterns can indicate commonality in regulatory control and/or biological functions.

**Fig. 3:**
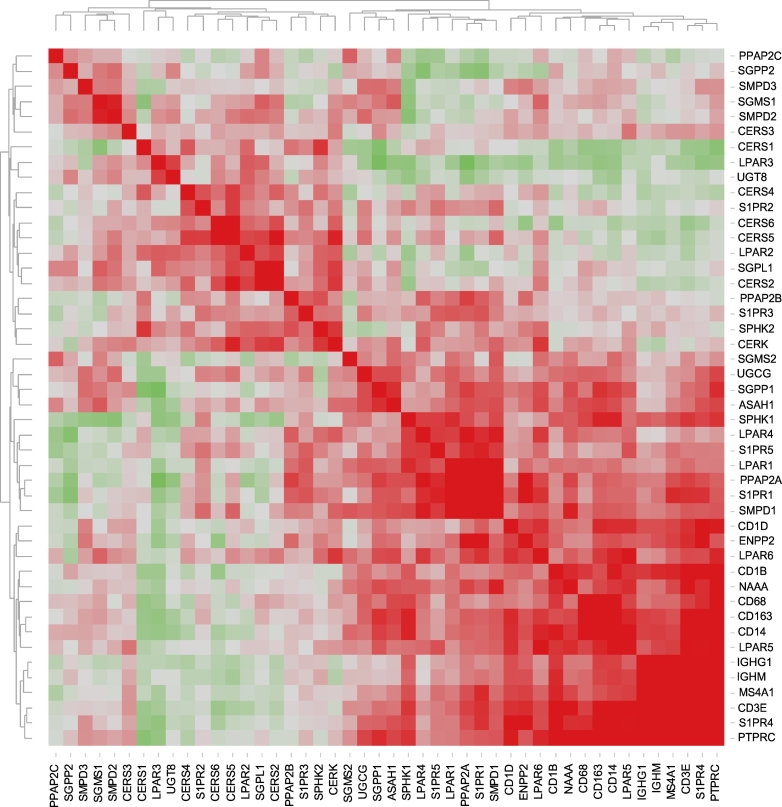
Heat map representing the correlation matrix for sphingolipid/lysophosphatidate/immune-associated genes. Pearson correlation matrix was calculated across all genes and the entire patient cohort (Table S2). The corresponding correlation coefficients were subjected to unsupervised hierarchical clustering using Euclidean distance measurement (average linkage clustering). Red-green color spectrum for correlation coefficients; each colored square illustrates a gene-gene interaction; the color intensity represents the correlation strength; color code: *red*, positive correlation; *green*, negative correlation. (For interpretation of the references to color in this figure legend, the reader is referred to the web version of this article.)

We also used hierarchical clustering of the gene-gene correlation matrix for further interpretation and visualization. Fig. 3 illustrates the heat map of associated correlation coefficients. Clustering reveals a separation into two major clusters, which are further subdivided into sub-clusters. Among the most significant positive associations, we found close associations among *PPAP2C*-*SGPP2*-*SMPD3*-*SGMS1*-*SMPD2*-*CERS3*, among *CERS4*-*S1PR2*-*CERS6*-*CERS5*-*LPAR2*-*SGPL1*-*CERS2* and *PPAP2B*-*S1PR3*-*SPHK2*-*CERK*, all within cluster I. There were also strong associations among *SPHK1*-*LPAR4*-*S1PR5*-*LPAR1*-*PPAP2A*-*S1PR1*-*SMPD1*, *CD1D*-*ENPPA*-*LPAR6* and *CD1B*-*NAAA*-*CD68*-*CD163*-*CD14*-*LPAR5*, and among *IGHG1*-*IGHM*-*MS4A1*-*CD3E*-*S1PR4*-*PTPRC* within cluster II. It is important to note that immune markers covering various immune cell populations and, thus, characterizing the on-site immune profile were positively correlated with each other and cluster together. These findings signify immune cell interactions that take place in the tumor microenvironment and point to sphingolipid/lysophosphatidate-related factors/mechanisms that, among others, could promote co-infiltration of immune cells of different lineages and/or their survival.

### 3.2 Clustering-based Patient Stratification and the Association with Chemotherapy Response

**Fig. 4:**
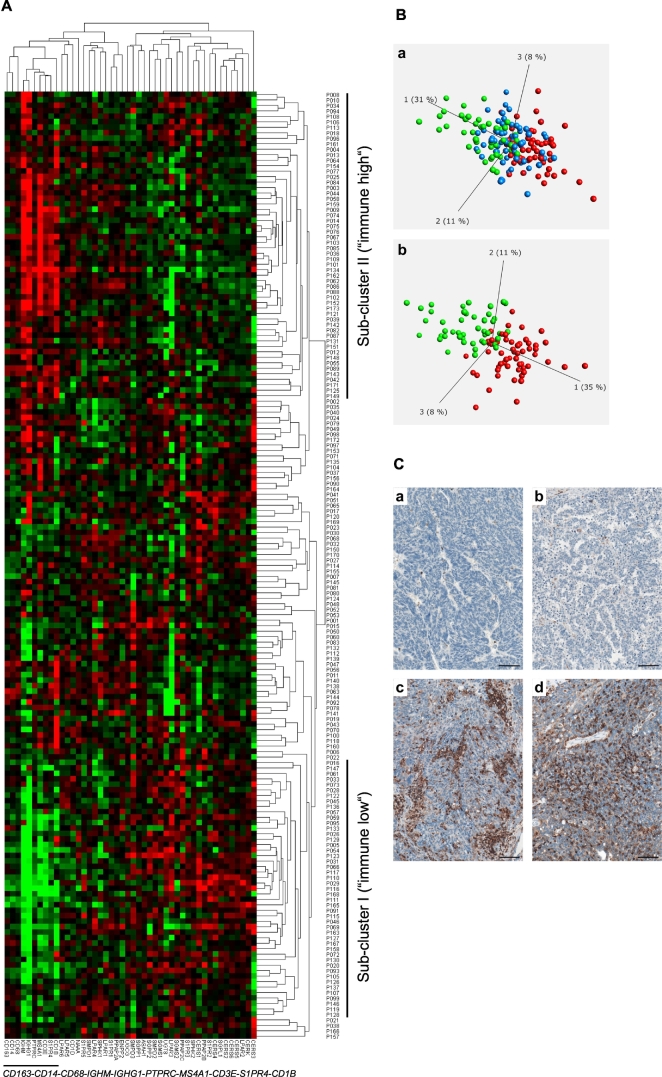
Clustering-based patient stratification. A Pearson uncentered hierarchical clustering was applied across log2 transformed expression values derived from sphingolid/immune-associated multigene signatures-based profiling and across the entire patient population. A result of a clustering run representing a pair of trees is shown for genes and patient samples (Cluster/TreeView programs). Color code: *red*, high expression and *green*, low expression according to Cluster/TreeView [91]. The two sub-clusters named as *immune low* and *immune high* as well as genes composing the immune-related sub-cluster are indicated. B A result is shown for PCA that was performed to visualize the data set in a three-dimensional space and identify significantly differential variables between 3 subgroups (a, immune low, mixed, and immune high) and 2 subgroups (b, immune low and immune high) which were obtained on the basis of hierarchical clustering. ANOVA was used for multigroup comparison and t-test for two-group comparison; a p-value cutoff of 0.05 was applied. Color code: green, immune low; blue, mixed; red, immune high. C Immunohistochemical staining of tissue sections obtained from patients with serous ovarian cancer (clinicopathological characteristics of patient cohort are given in Table S9); Module III in Fig. 1. Images of four representative specimens as shown for the immune low subgroup (a,b) with no/low infiltration of CD45-positive leukocytes and the immune high subgroup (c,d) with massive accumulation of CD45-positive leukocytes within the tumor tissue. Brown color, CD45 staining; blue color, nuclear counterstaining with hematoxylin. Scale bar: 100 μm. (For interpretation of the references to color in this figure legend, the reader is referred to the web version of this article.)

We next examined whether we could stratify (subdivide) the examined patient group into subgroups showing similarities in expression patterns on the basis of the 38/8-gene signature-derived expression profiles. We applied a hierarchical cluster analysis across all expression profiles of genes composing the signature and across the entire patient population. This algorithm arranged the data into a tree structure providing information about the relationship among the specimens and genes (Fig. 4A). Clustering revealed a clear separation of the patient group into sub-cluster I (47 out of 173 specimens), herein named as *immune low*. These are specimens that showed low expression of the immune-associated genes (*CD163*, *CD14*, *CD68*, *IGHM*, *IGHG1*, *PTPRC*, *MS4A1*, and *CD3E*) and two sphingolipid-associated genes (*S1PR4* and *CD1B*). Sub-cluster II (56 out of 173 specimens), herein named as *immune high*, consisted of specimens with high expression levels of the genes listed above. Additional subgroups of patients (70 out of 173 specimens) showed a mixed expression pattern (*mixed*).

**Fig. 5:**
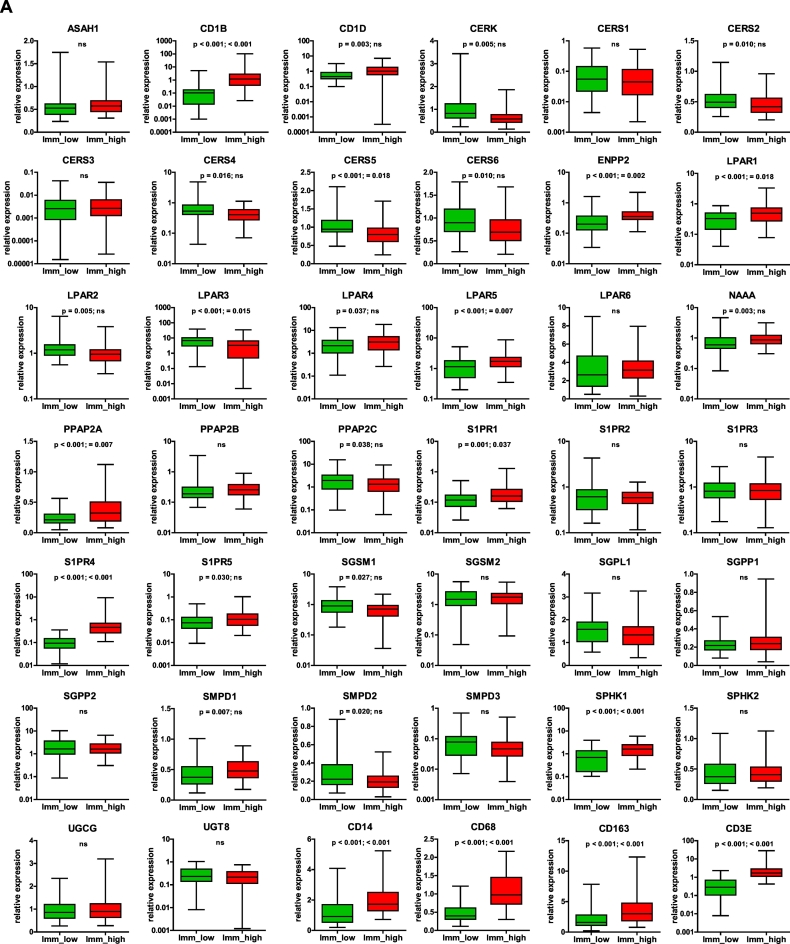

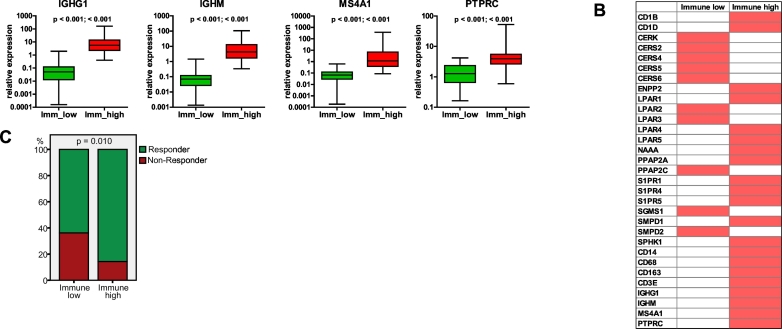
Comparative analysis of gene expression data sets in *immune low* versus *immune high* subgroups and the link to chemotherapy response. A Boxplots of mRNA expression levels for immune low (*n* = 47) and immune high (*n* = 56) subgroups are shown for all genes composing the sphingolipid/lysophosphatidate/immune-associated signature. The boxplot represents the distribution of values; the line across the box represents the median; the box stretches from the lower hinge (the 25th percentile) to the upper hinge (the 75th percentile). The *p*-values and the Bonferroni-Holm corrected *p*-values of two-sided t-test are indicated; ns, not significant. B Table summarizing the results of group comparison in (A). Only genes showing significant difference are included; considering this study as exploratory the non-corrected *p*-values were used. Red color indicates a higher level of expression within a patient subgroup. C Comparative assessment of contribution of responders and non-responders to the total quantity for the patient subgroups designated as immune low (*n* = 47) and immune high (*n* = 56); *p*-value of two-sided Chi-squared test is shown. (For interpretation of the references to color in this figure legend, the reader is referred to the web version of this article.)

The separation into 3 subgroups (*immune low*, *mixed*, and *immune high*) or into 2 subgroups (*immune low* and *immune high*) was additionally shown by Principal Component Analysis (PCA) (Fig. 4B, a or b, respectively). The *immune low* and *immune high* phenotypes were further illustrated by immunostaining of the paraffin-embedded tissue sections for CD45, a common leukocyte marker (Fig. 4C). Importantly, the two sub-clusters, *immune low* and *immune high*, were characterized by an inverse expression with respect to the additional sphingolipid/lysophosphatidate-associated genes with sub-cluster I showing overall a high expression and sub-cluster II an overall low expression. Statistical analysis was used for sub-cluster comparison with respect to the expression levels of each individual gene to verify the clustering outcome (Fig. 5A). Collectively, these data revealed specific expression patterns of sphingolipid/lysophosphatidate-associated genes characterizing the *immune low* and the *immune high* subgroups with *CD1B*, *CD1D*, *CERK*, *CERS2/4/5/6*, *ENPP2*, *LPAR1/2/3/4/5*, *NAAA*, *PPAP2A/C*, *S1PR1/4/5*, *SGSM1*, *SMPD1/2*, and *SPHK1* genes (*n* = 23) showing a significant difference as summarized in Fig. 5B. Furthermore, all immune-related genes under investigation (*n* = 8) showed highly significant differences.

Next, we explored whether there was a difference in clinicopathological parameters between patients found within the *immune low* versus *immune high* subgroups. We were intrigued to find an association with response to first-line chemotherapy with significantly more responders found in the *immune high* subgroup (*p* = .010, Fig. 5C). No statistically significant differences were found in respect to other clinicopathological parameters.

**Fig. 6:**
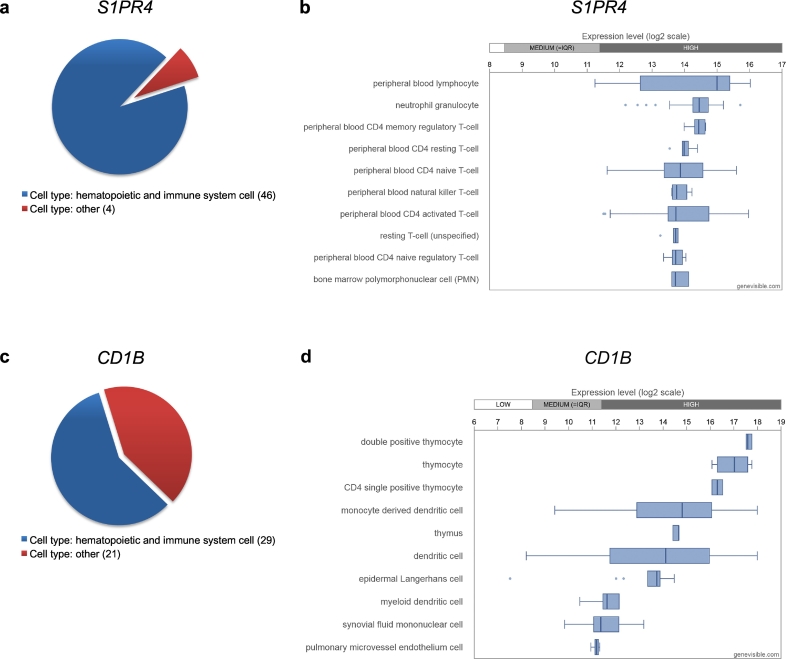
Integrative analysis of the expression patterns of *S1PR4* and *CD1B* across various cells types using microarray data sets. The GENEVESTIGATOR tool was used to assess the expression pattern of (*a*) *S1PR4* and (*c*) *CD1B* across all cell types under physiological conditions (filter: “Anatomy_Cell Type_All”; *n* = 10,490 arrays). Pie charts show the top 50 outcomes. Detailed illustration of the top 10 outcomes for (*b*) *S1PR4* and (*d*) *CD1B* was extracted via Genevisible.

Given that two sphingolipid-associated genes, *S1PR4* and *CD1B*, were found within the immune cluster, we performed integrative analysis using the GENEVESTIGATOR tool and assessed the expression pattern of these markers across all cell types. Others have previously suggested that S1PR4 is predominantly expressed by immune cells [63]. Our comprehensive analysis revealed that *S1PR4* is almost exclusively expressed by cells of the hematopoietic/immune cell linage (Fig. 6a) with a particular prevalence for various T-cell subsets as can be seen from the top 10 outcomes (Fig. 6b). Furthermore, our results show that the expression of *CD1B* is predominantly attributed to immune cells/immunological tissues (Fig. 6c, d). This is compatible with the major biological function of the protein encoded by this gene, which is an antigen-presenting protein that binds particular lipid antigens and mediates their presentation [54].

To assess whether a similar clustering pattern could be seen on signature-derived expression data sets from an independent EOC cohort (Table S3), we profiled 19 samples by real-time PCR assay using the 38/8-gene sphingolipid/lysophosphatidate/immune-associated signature. Hierarchical clustering revealed a similar separation of specimens into *immune low* and *immune high* subgroups on the basis of immune-related core genes (Fig. S1). The outcome emphasizes the reproducibility of our finding and demonstrates that the herein applied set of immune-related genes in conjunction with the sphingolipid/lysophosphatidate-associated signature is able to map the fundamental differences for immune-based subtypes of ovarian carcinoma and on the basis of this to stratify patients.

### 3.3 Univariate Associations of the Individual Gene Expression-derived Variables and the Clinicopathological Parameters with Overall Survival and Progression-free Survival

Univariate Cox regression analysis of overall survival (OS) and progression-free survival (PFS) was used to determine the clinical relevance of the gene expression data sets (*n* = 46 per patient). These were obtained on the basis of the sphingolipid/lysophosphatidate/immune -associated 38/8-gene signature and on clinicopathological parameters (*n* = 6) (Table S4). With respect to OS, three genes showed statistically significant associations, namely *LPAR3* (HR = 0.883, 95% CI: 0.804–0.969, *p* = .009), *SMPD2* (HR = 0.619, 95% CI: 0.433–0.884, *p* = .008), and *CD68* (HR = 0.824, 95% CI: 0.717–0.947, *p* = .006). All showed a positive prognostic effect of more favorable survival with higher gene expression. Among clinicopathological parameters, four variables showed significant association with OS (Table S4). With respect to PFS, the gene *CD1B* (HR = 0.936, 95% CI: 0.880–0.996, *p* = .037) and four clinicopathological parameters showed significant association (Table S4). Of note, the subsequent multivariable modeling using Cox regression with ridge and LASSO penalties was not based on univariate pre-selection of variables.

### 3.4 Multivariable Prognostic Models for OS and PFS

As the next step of the MuSiCo approach, we developed multivariable survival models for evaluating patient prognosis and stratifying patients into risk groups. We previously applied this strategy to dissect the role of the AID/APOBEC gene network in ovarian cancer and it was described in detail [49].

**Fig. 7:**
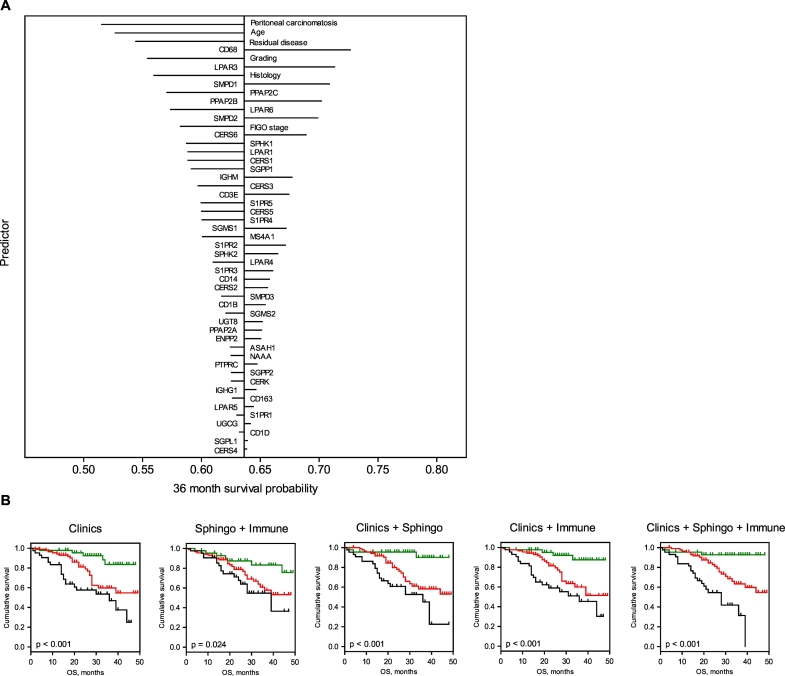
Multivariable prognostic models. A Impact of individual variables on survival prediction of the Clinics + Sphingo + Immune multivariable model (ridge) for OS if predictors are changed by +1 SD. Survival probabilities are estimated at 36 months of follow-up time. Variables are ranked according to the absolute changes in prediction. The length of the lines is proportional to the change in prediction in case the value of the indicated variable changes by +1 SD (left: negative effect; right: positive effect). B Kaplan-Meier estimates for patient stratification based on the multivariable models (ridge) for OS. Kaplan-Meier curves for OS are shown giving patients' stratification into low risk (green), intermediate risk (red), and high risk (black) groups with the 25th and the 75th percentiles serving as thresholds (lower than the 25th percentile indicates low risk); p-value of the log-rank test is indicated. Kaplan-Meier estimates are not shown for models that were not statistically significant (Table 2). (For interpretation of the references to color in this figure legend, the reader is referred to the web version of this article.)

Modeling results. Briefly, multivariable Cox regression was applied to develop prognostic models using regularization with ridge and LASSO penalties. Calculations were based on: (i) the clinicopathological parameters, (ii) the gene profiling-derived sphingolipid-associated variables, (iii) the gene profiling-derived immune-associated variables, and (iv) all possible combinations; in a total of 7 models. The results for the multivariable ridge and LASSO models are summarized in Table 2 and Table S5, respectively. Both regularization methods showed similar predictive abilities. The outcome of the Cox regression with LASSO penalties is summarized in Supplementary Data (Table S5, Table S6 and Fig. S2). Herein, we describe the results of the Cox regression with the ridge penalties. The modeling strategy allowed us to rank the contribution of the individual variables to the overall prognostic effect of the model according to their standardized regression coefficients (Table S7) or according to the absolute change in 36 month survival probability (Fig. 7A). Within the *Clinics + Sphingo + Immune* model, besides the clinicopathological variables, the sphingolid/lysophosphatidate/immune-associated variables were found in the top positions (Table S7). This includes *CD68* (position 3), *LPAR3* (position 5), *SMPD1* (position 6), *PPAP2B* (position 9), and *SMPD2* (position 10). Of note, for a particular model the overall prognostic effect was based on adding the independent prognostic effects of the individual variables. The effect was more pronounced for those variables which showed an independent impact and thus are mutually supportive. Thus, in case of a signature-based model, genes found at the top positions are likely to be attributed to diverse pathobiological pathways/mechanisms.

**Table 2:** Comparative analysis of multivariable models (ridge) for prognostication of OS and PFS. Cross-validated performance assessment of Cox regression models (ridge) by proportion of explained variation (PEV), concordance index (c-index) and p-value.

	OS			PFS		
	PEV %	c-index	P	PEV %	c-index	P
Clinics	**10.29**	0.704	< 0.001	15.10	0.668	< 0.001
Sphingo	1.24	0.580	0.116	–	–	–
Immune	0.84	0.508	0.373	–	–	–
Sphingo + Immune	**2.44**	**0.602**	**0.036**	–	–	–
Clinics + Sphingo	**13.77**	**0.727**	**< 0.001; 0.004**⁎	**13.91**	**0.652**	**< 0.001**; 0.099⁎
Clinics + Immune	**14.65**	**0.725**	**< 0.001; 0.002**⁎	**13.48**	**0.657**	**< 0.001**; 0.726⁎
Clinics + Sphingo + Immune	**19.34**	**0.751**	**< 0.001; < 0.001**⁎	**12.45**	**0.644**	**< 0.001**; 0.188⁎

⁎ *p*-Value for added value of Sphingo or Immune or Sphingo + Immune on top of Clinics in bivariable models with cross-validated predictors.Statistically significant outcomes are shown in bold.

Model validation. In leave-one-out cross-validation with respect to OS, the clinicopathological variables-based model showed moderate predictive accuracy (*Clinics*; PEV = 10.29%, c-index = 0.704, *p* < .001). No statistically significant models could be built on the basis of either sphingolipid-associated variables (*Sphingo*) or immune-associated variables (*Immune*). A weak but significant predictive accuracy was observed for their combination (*Sphingo *+* Immune*; PEV = 2.44%, c-index = 0.602, *p* = .036). The combination of clinicopathological variables with sphingolipid-associated variables (*Clinics *+ *Sphingo*; PEV = 13.77%, c-index = 0.727, *p* < .001) and with immune-associated variables (*Clinics *+* Immune*; PEV = 14.65%, c-index = 0.725, *p* < .001) showed a moderate/high predictive accuracy. The combined model of clinicopathological variables, sphingolipid-associated variables, and immune-associated variables had the highest predictive accuracy (*Clinics *+* Sphingo *+* Immune*; PEV = 19.34%, c-index = 0.751, *p* < .001). With respect to PFS, the inclusion of the sphingolid/immune-associated signature-based variables did not significantly improve the prognostic power of the clinicopathological variables for survival (Table 2).

Next, patients were sub-divided according to their cross-validated predictors for OS into low, intermediate, and high-risk groups. As shown by Kaplan-Meier estimates (Fig. 7B) the combined model (*Clinics *+* Sphingo *+* Immune*) gave a major improvement of patient stratification and showed statistically significant differences between the risk groups (log-rank test: *p* < .001).

With respect to treatment response, the inclusion of the sphingolipid/lysophosphatidate/immune-associated signature-based variables did not improve on the model with only clinicopathological variables (Table S8).

### 3.5 Expression of Genes Composing the Sphingolipid/Lysophosphatidate/Immune-associated Signature in 55 Ovarian Cancer Cell Lines

**Fig. 8:**
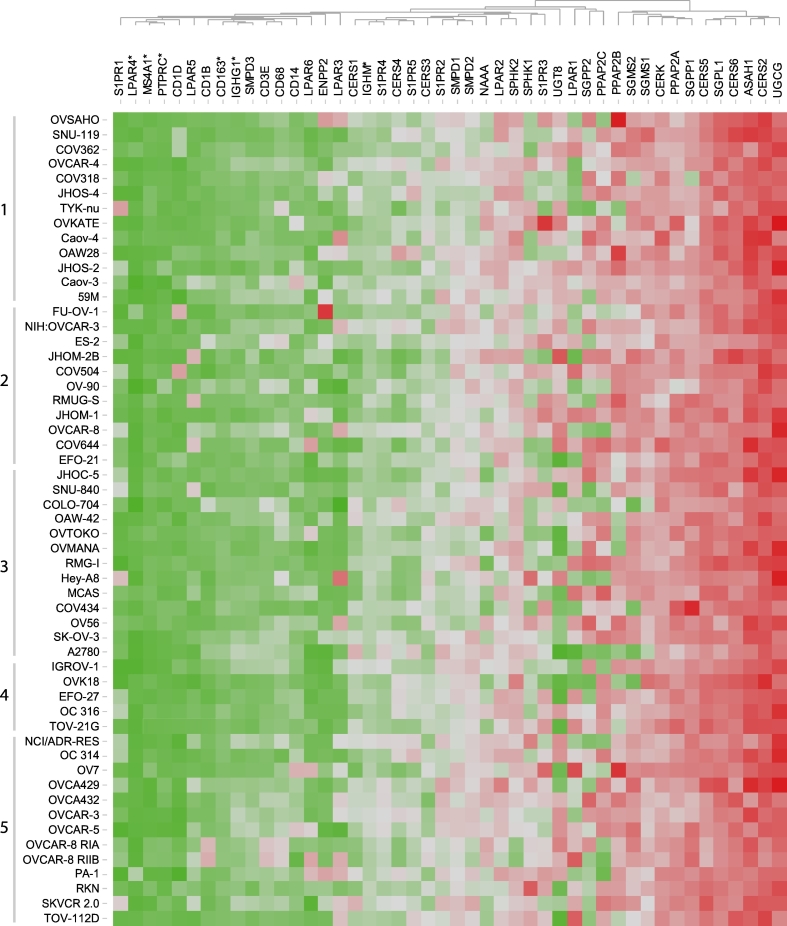
Heat map representing the expression profiles of genes composing the sphingolipid/lysophosphatidate/immune-associated signature in a wide range of ovarian cancer cell lines. Expression values (log2 transformed) for individual genes across 55 ovarian cancer cell lines were extracted using GENEVESTIGATOR. Expression values were subjected to unsupervised hierarchical clustering using Euclidean distance measurement (average linkage clustering). Color code: *red*, high expression (max 17.25); *green*, low expression (min 7.13). Genes exhibiting expression at microarray detection limit are indicated by asterisks. Cell lines were grouped into five sub-categories according to Domcke S. et al. [64]: *1*, likely high-grade serous; *2*, possibly high-grade serous; *3*, unlikely high-grade serous; *4*, hyper-mutated; *5*, unclassified. (For interpretation of the references to color in this figure legend, the reader is referred to the web version of this article.)

Given the complexity of ovarian cancer tissue in respect of cellular composition, we assessed mRNA expression levels of sphingolipid/lysophosphatidate/immune-associated genes across a wide range of ovarian cancer cell lines (*n* = 55). This determined the expression profile attributed to cancer cells only. The GENEVESTIGATOR platform allowed us to extract the expression values from previously published microarray data sets. The panel of ovarian cancer cell lines included, among others, those cell lines that were evaluated by Domcke et al. [64] on the basis of genomic profiling. These were sub-grouped by their suitability as high-grade serous ovarian cancer models. With the exception of six genes (*LPAR4*, *CD163*, *IGHG1*, *IGHM*, *MS4A1*, and *PTPRC*), that showed low expression and/or expression at the microarray detection limit, the genes composing the sphingolipid/lysophosphatidate/immune-associated signature were expressed in various cell lines (Fig. 8 and Fig. S3). There were no statistically significant differences in the expression levels of analyzed genes across the sub-categories of ovarian cancer cell lines defined by Domcke et al.

### 3.6 Expression Pattern of the Top Candidate Molecules in Ovarian Cancer Tissues: the Pilot Study to Expand Gene Expression Data to Imaging and to a System Approach

**Fig. 9:**
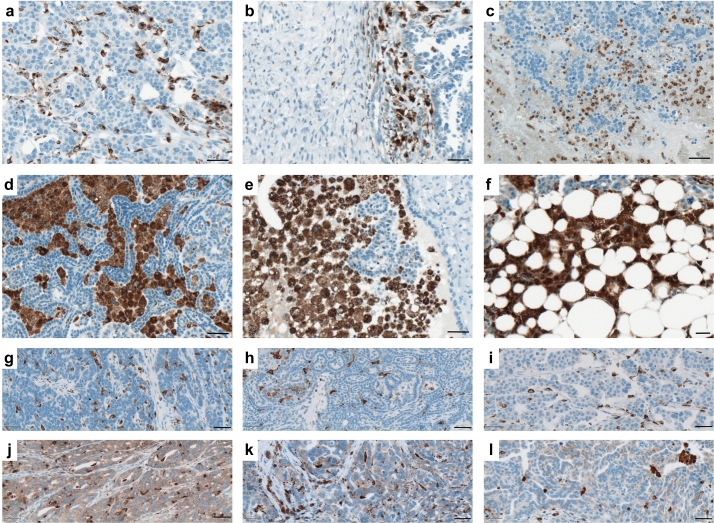
Localization patterns and morphology of CD68-positive cells of the monocyte/macrophage lineage at tumor site and the CD68 expression by tumor cells. Immunohistochemical staining of tissue sections that were obtained from patients with serous ovarian cancer. CD68 belongs to the lysosomal/endosomal-associated membrane glycoprotein (LAMP) family and primarily localizes to lysosomes and endosomes with a smaller fraction circulating to the cell surface. Representative images of various sub-populations of CD68-positive leukocytes are shown: (*a*) elongated well-spread intra-tumoral macrophages, (*b*) macrophage accumulation at the tumor – stroma border, (*c*) accumulation of round shaped monocytes in mucous tissue, (*d*,*e*) large phagocytes organized into phagocytic islands, (*f*) accumulation of macrophages in adipose tissue. Expression of CD68 by tumor cells: representative images of ovarian cancer specimens (*n* = 6) showing (*g*-*i*) the absence or (*j*-*l*) presence of CD68 expression by tumor cells. Brown color, CD68 staining; blue color, nuclear counterstaining with hematoxylin. Scale bar: 50 μm. (For interpretation of the references to color in this figure legend, the reader is referred to the web version of this article.)

In this exploratory study, we aimed to determine the tumor anatomy-attributed expression pattern of the top 3 candidate molecules – CD68, SMPD1, and LPAR3 – within complex ovarian cancer tissues. We used a panel of paraffin-embedded specimens from an independent patient cohort (Table S9) for immunohistochemical staining. For CD68, evaluation of the entire slides/tissue specimens for the on-site immune response revealed strong inter-patient variability regarding spatial distribution of CD68-positive immune cells, their organization pattern, and morphology. We found CD68-positive cells as well-spread intra-tumoral macrophages (Fig. 9a) and those at the tumor – stroma border (Fig. 9b), as small round monocytes in mucous tissue (Fig. 9c), as large phagocytes organized into phagocytic islands (Fig. 9d-e), and as macrophages accumulated within adipose tissue (Fig. 9f). In addition to cells of the monocyte/macrophage lineage in a subgroup of patients, we detected the presence of CD68-positive tumor cells (Fig. 9 j-l; to compare to CD68-negative tumors, Fig. 9g-i). This observation agrees with previously published data [65], and the present results showing moderate expression of *CD68* in some ovarian cancer cell lines at the mRNA level (Fig. S3).

**Fig. 10:**
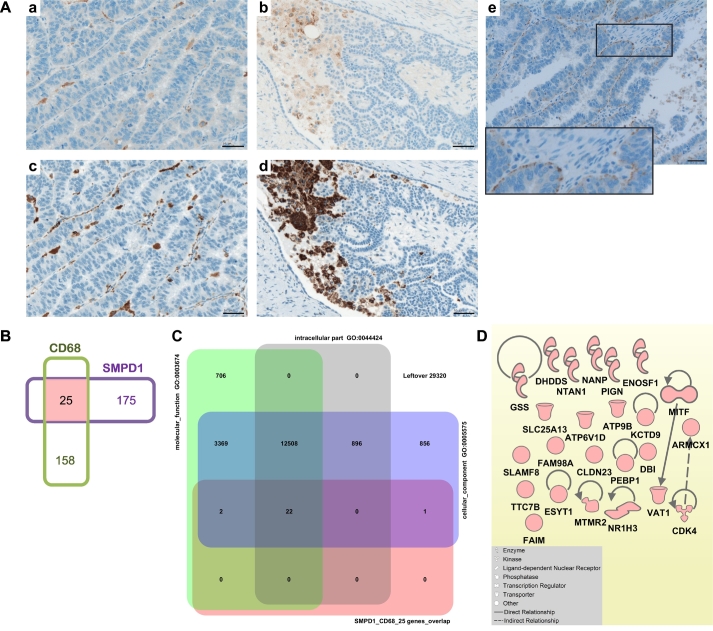
Similarity and overlap in expression patterns between SMPD1 and CD68. A Immunohistochemical staining of tissue sections obtained from patients with serous ovarian cancer; Module III in Fig. 1. Consequent tissue sections were stained for SMPD1 and CD68. Representative images show the overlap in expression for intra-tumoral macrophages (a, SMPD1; c, CD68) and phagocytes organized into phagocytic islands (b, SMPD1; d, CD68). Furthermore, (e) example of SMPD1 expression by the tumor cells is shown; staining was detected at the basolateral side of the epithelial layer; insert: the high-power view. Brown color, SMPD1 or CD68 staining; blue color, nuclear counterstaining with hematoxylin. Scale bar: 50 μm. B Venn diagram shows overlap between genes co-regulated with CD68 and SMPD1 in monocytes/macrophages. For both, CD68 and SMPD1, the top 200 co-regulated Affymetrix probes sets were extracted via GEVESTIGATOR from microarray studies exploring gene expression in monocytes/macrophages (described in detail in Methods). Comparison was done on the basis of the corresponding gene names (n = 183 for CD68; n = 200 for SMPD1). The overlap (color code: rose) between the two lists includes the following 25 genes: CLDN23, ARMCX1, ATP9B, ATP6V1D, MTMR2, KCTD9, TTC7B, SLC25A13, VAT1, NTAN1, MITF, ENOSF1, NR1H3, UNC13B, CDK4, GSS, DHDDS, SLAMF8, NANP, PEBP1, FAM98A, FAIM, ESYT1, DBI, PIGN. C Gene enrichment analysis for Gene Ontology (GO) using GENEVESTIGATOR gene set enrichment tool. The Venn diagram displays the input gene set (SMPD1_CD68_25 genes_overlap; color code: rose) and the top 3 gene sets from the background collection (color code: grey, violet, green). The respective GO terms and annotations are provided. The numbers indicate how many genes are found in overlaps. (D) Gene network reconstruction using the Ingenuity Pathway Analysis Software (IPA) on the basis of the gene set SMPD1_CD68_25 genes_overlap; Module V in Fig. 1. Insert: the IPA-based description of symbols and relationships. (For interpretation of the references to color in this figure legend, the reader is referred to the web version of this article.)

An important additional finding describes the strong similarity in expression pattern between SMPD1 and CD68 for cells of the monocyte/macrophage lineage at the tumor site (Fig. 10A, a and c, b and d). Furthermore, we found SMPD1 expression in tumor cells; the staining showed a polarized appearance towards the basolateral side of the epithelial layer (Fig. 10A, e). To further investigate potential crosstalk between CD68- and SMPD1-attributed molecular events in monocytes/macrophages, we extracted the co-expressed genes for each molecule from microarray studies exploring gene expression using GENEVESTIGATOR and applying the filter “Anatomy_Cell Type_Hematopoietic and Immune Cell_Leukocyte_ Myeloid Leukocyte_Monocyte/Macrophage”. We found 25 overlapping genes (Fig. 10B) by comparing the lists of the top 200 co-expressed probe sets (Table S10 and Table S11). We used the 25-gene set as input to perform gene enrichment analysis by comparing with background collection of gene sets related to known biological processes, conditions or pathways. By using the gene set enrichment tool from GENEVESTIGATOR related to Gene Ontology categories, we found cellular_component (GO: 0005575), intracellular_part (GO: 00044424), and molecular_function (GO: 0003674) among the top 3 most significant outcomes. These associations indicated that gene products could be located in particular parts of a cell within a particular macromolecular complex and they could function together (Fig. 10C). Furthermore, we used the Ingenuity Pathway Analysis software (IPA) to investigate the relationships among components of the 25-gene set and reconstructed a gene network. Fig. 10D shows the limited knowledge of the crosstalk between CD68- and SMPD1-attributed molecular events represented by a limited number of IPA-identified associations.

**Fig. 11:**
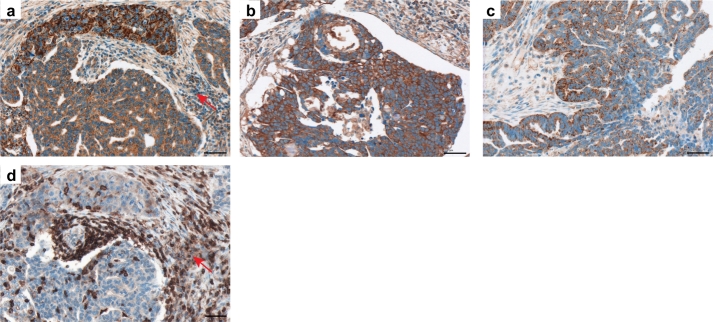
Expression of LPAR3 in ovarian cancer specimens. Immunohistochemical staining of tissue sections obtained from patients with serous ovarian cancer. Representative images of LPAR3 staining of three ovarian cancer specimens are shown (*a*-*c*). The tumor-infiltrating leukocytes are indicated by an arrow in (*a*) and visualized by anti-CD45 staining of consecutive slide in (*d*). Brown color, LPAR3 or CD45 staining; blue color, nuclear counterstaining with hematoxylin. Scale bar: 50 μm. (For interpretation of the references to color in this figure legend, the reader is referred to the web version of this article.)

With respect to the additional top candidate molecule, LPAR3, we found heterogeneous expression by various cell types within complex ovarian cancer tissue. There was strong expression in cancer cells, but also in stroma cells including tumor-infiltrating leukocytes (Fig. 11a–c). The staining patterns suggest heterogeneous expression levels in tumor islands and a gradient in expression for the individual tumor parts. As described above, strong heterogeneity in *LPAR3* mRNA expression levels was detected within a panel of 55 ovarian cancer cell lines (Fig. S3).

We conclude that the three molecules with the highest impact to the combined prognostic model show tumor anatomy- and patient-specific characteristics in their expression patterns with potentially complementary molecular events for CD68 and SMPD1 taking place in cells of the monocyte/macrophage lineage.

### 3.7 Integrated Analysis of the Sphingolipid/Lysophosphatidate/Immune-associated Expression Profile under Diverse Neoplastic Conditions Using Curated Microarray Data Sets from Public Databases

To assess whether the defined sphingolipid/immune-associated signature was specific for ovarian cancer or if it represented a general cancer-attributed transcriptional profile, we examined the similarities with expression profiles across previously published transcriptomic data sets using the GENEVESTIGATOR platform. The comparison was based on the measurement of a distance that quantifies the degree of similarity between the defined signature profile and the transcriptional profile for a condition. The signature profile was given by the median value of expression levels, calculated across the patient cohort (*n* = 173), for each gene of the sphingolipid/lysophosphatidate/immune-associated 38/8-gene signature. A signature analysis using the cancer subset of Affymetrix Human 133 Plus 2 microarrays (*n* = 21,095 arrays) including over 600 different neoplasm categories designated herein as “Cancer_All” revealed that among the top 10 correlated conditions, 9 are attributed to neoplasms of the ovary (6/10), of the corpus uteri/endometrium (2/10), and of the peritoneum (1/10). One study (ranked at position 10) was attributed to meningioma, a neoplasm of the brain (Table 3). We next examined the specificity using the independent patient cohort (*n* = 19, Table S3) and found within the top 10 most correlated conditions only studies attributed to neoplasms of the ovary (8/10), of the corpus uteri/endometrium (1/10), and the peritoneum (1/10) (Table S12). These results collectively indicate a high specificity of the sphingolipid/lysophosphatidate/immune-associated 38/8-gene signature for ovarian cancer.

**Table 3:** The top 10 sphingolipid/lysophosphatidate/immune signature-linked neoplasms identified by GENEVESTIGATOR for the main patient cohort (n = 173). GENEVESTIGATOR nomenclature is used for malignant disorders. The top 10 results are shown; the ranking is based on the corresponding GENEVESTIGATOR-based relative similarity (Rel. Similarity). Tissue type, study number, and reference(s) annotating the corresponding studies are indicated.

Position	Study	Tissue type	Rel. Similarity	Study Number	Reference
1	Papillary serous cystadenocarcinoma, borderline malignancy	Ovary	1.409	GSE9899	[1]
2	Serous cystadenocarcinoma, NOS	Ovary	1.408	GSE2109,	part in [2, 3]
				GSE12172,	[4]
				GSE20565,	[5]
				GSE19352,	[6]
				GSE36668,	[7]
				GSE63885,	[8, 9]
				GSE32062	[10]
3	Endometrioid carcinoma, metastatic	Endometrium	1.387	GSE2109	part in [2, 3]
4	Papillary serous cystadenocarcinoma, metastatic	Peritoneum	1.382	GSE2109,	part in [2, 3]
				GSE9899	[1]
5	Papillary serous cystadenocarcinoma, metastatic	Ovary	1.367	GSE2109	part in [2, 3]
6	Endometrioid carcinoma	Ovary	1.351	GSE2109,	part in [2, 3]
				GSE9899,	[1]
				GSE20565,	[5]
				GSE63885	[8, 9]
7	Serous cystadenocarcinoma, NOS, unstated behavior	Ovary	1.350	GSE26193	[11]
8	Endometrioid carcinoma	Endometrium	1.325	GSE2109	part in [2, 3]
9	Serous cystadenocarcinoma, NOS, metastatic	Ovary	1.317	GSE2109,	part in [2, 3]
				GSE12172	[4]
10	Meningioma, NOS	Meninges	1.312	GSE4780,	[12]
				GSE9438,	[13]
				GSE16584	[14]

1. Tothill, R.W., et al., *Novel molecular subtypes of serous and endometrioid ovarian cancer linked to clinical outcome.* Clin Cancer Res, 2008. 14(16): p. 5198–208.

2. Kharma, B., et al., *Utilization of genomic signatures to identify high-efficacy candidate drugs for chemorefractory endometrial cancers.* Int J Cancer, 2013. 133(9): p. 2234–44.

3. Yamamura, S., et al., *The activated transforming growth factor-beta signaling pathway in peritoneal metastases is a potential therapeutic target in ovarian cancer.* Int J Cancer, 2012. 130(1): p. 20–8.

4. Anglesio, M.S., et al., *Mutation of ERBB2 provides a novel alternative mechanism for the ubiquitous activation of RAS-MAPK in ovarian serous low malignant potential tumors.* Mol Cancer Res, 2008. 6(11): p. 1678–90.

5. Meyniel, J.P., et al., *A genomic and transcriptomic approach for a differential diagnosis between primary and secondary ovarian carcinomas in patients with a previous history of breast cancer.* BMC Cancer, 2010. 10: p. 222.

6. Iorio, E., et al., *Activation of phosphatidylcholine cycle enzymes in human epithelial ovarian cancer cells.* Cancer Res, 2010. 70(5): p. 2126–35.

7. Elgaaen, B.V., et al., *ZNF385B and VEGFA are strongly differentially expressed in serous ovarian carcinomas and correlate with survival.* PLoS One, 2012. 7(9): p. e46317.

8. Lisowska, K.M., et al., *Gene expression analysis in ovarian cancer - faults and hints from DNA microarray study.* Front Oncol, 2014. 4: p. 6.

9. Lisowska, K.M., et al., *Unsupervised analysis reveals two molecular subgroups of serous ovarian cancer with distinct gene expression profiles and survival.* J Cancer Res Clin Oncol, 2016. 142(6): p. 1239–52.

10. Yoshihara, K., et al., *High-risk ovarian cancer based on 126-gene expression signature is uniquely characterized by downregulation of antigen presentation pathway.* Clin Cancer Res, 2012. 18(5): p. 1374–85.

11. Mateescu, B., et al., *miR-141 and miR-200a act on ovarian tumorigenesis by controlling oxidative stress response.* Nat Med, 2011. 17(12): p. 1627–35.

12. Stuart, J.E., et al., *Identification of gene markers associated with aggressive meningioma by filtering across multiple sets of gene expression arrays.* J Neuropathol Exp Neurol, 2011. 70(1): p. 1–12.

13. Claus, E.B., et al., *Specific genes expressed in association with progesterone receptors in meningioma.* Cancer Res, 2008. 68(1): p. 314–22.

14. Lee, Y., et al., *Genomic landscape of meningiomas.* Brain Pathol, 2010. 20(4): p. 751–62.

We applied the same approach to assess whether there was a difference in the sphingolipid/lysophosphatidate/immune-associated transcriptional profile between the patients within the low and the high risk groups stratified by Kaplan-Meier estimates (Fig. 7B) on the basis of the combined model. For the low risk group (*n* = 43), 10/10 studies within the top correlated conditions were attributed to neoplasms of the ovary, endometrium, and peritoneum (Table S13). For the high risk group (*n* = 43), similarity to neoplasms of other tissue origins was also observed (Table S14). We conclude that there was a difference in the expression profile across the signature genes, which was associated with differences in OS.

### 3.8 Dissection of the Data-driven Signature-associated Pathways and Upstream regulators and Reconstruction of the Sphingolipid/Lysophosphatidate/Immune-associated Gene Network

**Fig. 12:**
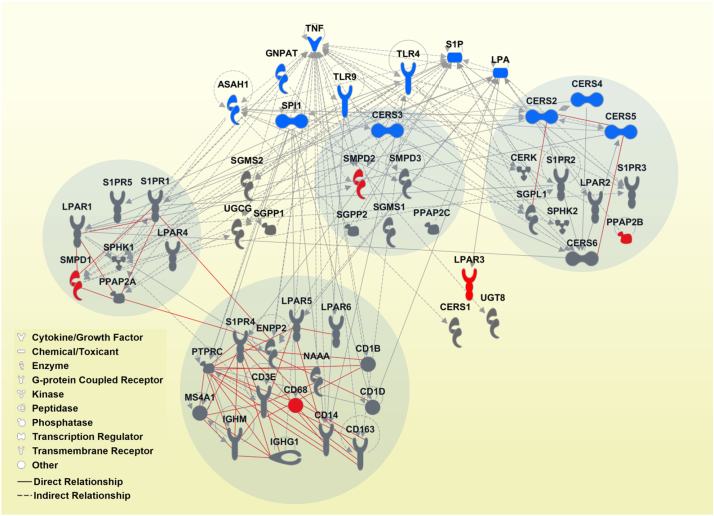
Sphingolipid/lysophosphatidate/immune-associated gene network. A reconstructed gene network was created using the IPA software on the basis of genes composing the sphingolipid/lysophosphatidate/immune-associated signature and the top IPA-predicted Upstream Regulators. Grey solid lines display the IPA-identified direct associations between molecules; grey dashed lines display the IPA-identified indirect associations between molecules. Additionally, statistically significant, herein defined biological associations assessed by correlation analysis (correlation coefficient > 0.5, *p* < .001; Table S2) are displayed by red solid lines. Color code: *grey fill*, genes composing the multigene signature; *red fill*, genes with strong prognostic effect (within the top 10 variables of the model *Clinics + Sphingo + Immune* for OS); *blue fill*, the top IPA-predicted Upstream Regulators (*ASAH1*, *CERS2*, *CERS3*, *CERS4*, *CERS5* were within the top IPA-predicted Upstream Regulators and are also part of the multigene signature). Grey circles indicate the groups of genes composing the main sub-clusters (> 5 genes, Fig. 3) identified by hierarchical clustering. Insert: the IPA-based description of symbols and relationships. (For interpretation of the references to color in this figure legend, the reader is referred to the web version of this article.)

To extend the signature- and modeling-derived knowledge and gain a higher-level overview, we applied a systems biology approach using IPA-based ‘core analysis’. We aligned the sphingolipid/lysophosphatidate/immune-associated data sets with IPA's Canonical Pathways and Upstream Regulators (complete lists of significant outcomes are given in Table S15 and Table S16, respectively). The top 10 Canonical Pathways were assigned to *Ceramide Signaling*, *Sphingosine-1-phosphate Signaling*, *Sphingomyelin Metabolism*, *Sphingosine and Sphingosine-1-phosphate Metabolism*, *RhoA Signaling*, *Gα12/13 Signaling*, *Human Embryonic Stem Cell Pluripotency*, *eNOS Signaling*, *Primary Immunodeficiency Signaling*, and *Lipid Antigen Presentation by CD1*. Furthermore, we reconstructed the interaction network on the basis of the signature genes and IPA-derived Upstream Regulators. Fig. 12 consolidates the outcomes of the integrative IPA-based analysis, the multivariable modeling, and the hierarchical cluster analysis of expression data sets. We highlighted the top genes contributing to the combined survival model with the strongest prognostic impact as node genes and indicated the groups of genes composing the main sub-clusters identified by hierarchical clustering. Noteworthy is the finding that each node gene is allocated in a separate sub-cluster, likely accentuating the independent and thus complementary impact of each top gene to the overall prognostic effect of the survival model.

## 4 Discussion

Our conclusions are based on novel insights gained during this study which deepen our knowledge on the multifaceted roles of the sphingolipid/lysophosphatidate system in the regulation of pathobiological mechanisms of cancer and the local immune response. We applied the sphingolipid/lysophosphatidate/immune-associated multigene signature within the integrative MuSiCo algorithm and demonstrated the applicability of the patient-specific, signature-derived gene expression data sets for identification of novel sphingolipid/lysophosphatidate/immune-related, disease-relevant aberrations and checkpoints. The comprehensiveness of analysis was herein strengthened by inclusion of next generation digital pathology/digital imaging module and by application of the Signature Tool for assessment of specificity of a transcriptional pattern.

Although many efforts have been invested in the field of sphingolipids and cancer, our approach is different in the following major aspects. First, the sphingolipid-related molecules and mediators, comprising the sphingolipid machinery, were considered as “system” with all those principals and parameters, which are valid for systems in general. The sphingolipid system is an open dynamic system that has multilevel interactions and interdependences among the components. Second, the sphingolipid- and other lysophosphatidate-related genes were jointly analyzed, reflecting an assumption about the exchange of information between the sphingolipid and lysophosphatidate systems. Third, we did not limit the analysis to either sphingolipid/lysophosphatidate system or local immune system but rather went a step further and tested the on-site interconnections and interdependences between the two biological systems. Fourth, tumor samples were profiled using a “focused” sphingolipid/lysophosphatidate/ immune gene set that does not include any tissue-specific cancer genes. This ensures the wide applicability of the algorithm.

The data presented in this study indicate that the features of the program established by the local sphingolipid machinery give an important impact to the organization of the ovarian cancer microenvironment by way of shaping immune infiltrates. Our results revealed a clear division of ovarian cancer patients into subgroups with no/low versus high levels of immune-related markers. Remarkably, for the latter there is a clear interconnection between various types of immune infiltrates. Specifically, the markers that represent the CD45-positive leukocytes or various resident/infiltrating immune cell lineages as well as subsets tend to cluster together. The subsets include B cells/CD20 and also B-cell-related IgG and IgM mature transcripts, T cells/CD3E, and the monocyte/macrophage lineage covered by CD14, CD68, and CD163. The tumor specimens thereby have been stratified by clustering into *immune high* and *immune low* subtypes, which have highly significant differences in mRNA expression levels of the indicated immune markers. This demonstrates that among primary serous ovarian tumors there are those who are under continual on-site immune pressure and those who are not. The first are characterized by: (i) a high magnitude of infiltrating T cells and B cells including the antigen-instructed memory cells and/or plasma cells, accompanied by the presence of high magnitudes of monocytes and macrophages, and potentially also CD68-positive dendritic cells; (ii) antigen presentation to T cells including enhanced presentation of lipid antigens; and generally, by (iii) multilevel immune editing of malignant cells.

**Fig. 13:**
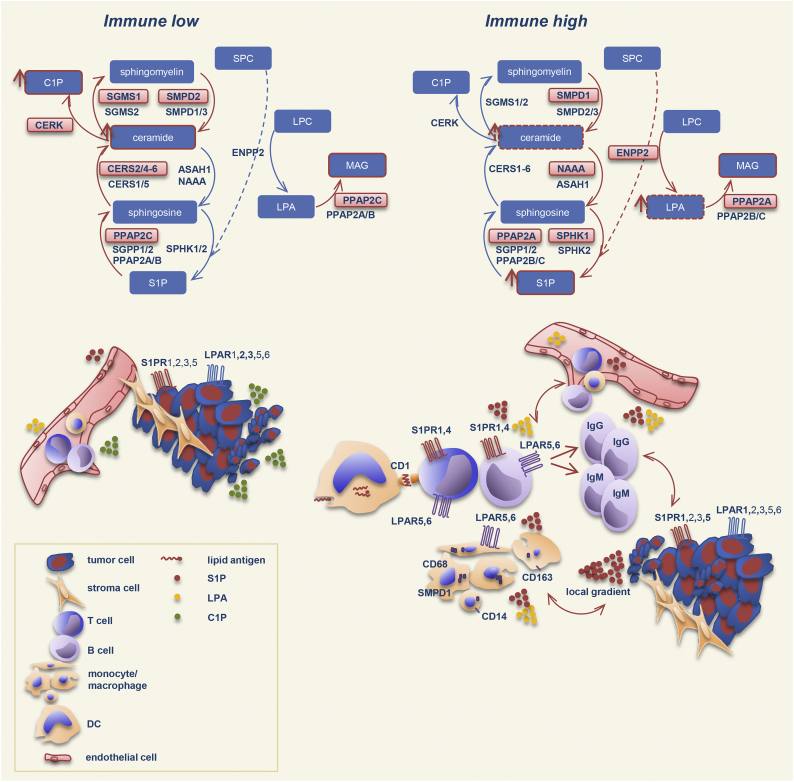
A partly hypothetical model describing the characteristic shifts of balance within the sphingomyelin/salvage pathway attributed to *immune low* and *immune high* tumor subtypes/subgroups of patients. The scheme shows the interconnected network of the sphingolipid mediators and the corresponding sphingolipid-modifying enzymes; those genes that are upregulated in either subgroup (Fig. 5) are highlighted in red. Furthermore shown is a schematic illustration of cellular interplay at the tumor site. The model consolidates cumulative data from the correlation analysis and unsupervised hierarchical clustering for sphingolipid/lysophosphatidate/immune-associated genes, clustering-based patient stratification and comparative analysis of gene expression data sets, expression profiles of genes composing the sphingolipid/lysophosphatidate/immune-associated signature in ovarian cancer cell lines, and integrative analysis of the expression patterns across transcriptomic data sets. SPC, sphingosylphosphorylcholine; LPC, lysophosphatidylcholine. (For interpretation of the references to color in this figure legend, the reader is referred to the web version of this article.)

There is, however, another sharp distinction between the two tumor subtypes. This is attributed to the differential expression of the sphingolipid-related genes within the sphingomyelin/salvage pathway as well as S1PRs and LPARs (Fig. 13). The sphingolipid-related gene signature of the *immune low* tumor subtype is preferentially attributed to tumor, stroma, and other ovarian tissue-specific cells composing the highly heterogeneous and complex ovarian tumor tissue. This group, by its definition, minimizes the contribution of immune cells. We showed that the *immune low* tumors were characterized by increased levels of *CERS2/4-6* encoding three different ceramide synthases. These enzymes synthesize different ceramide species that differ in the lengths and extent of fatty acid saturation. As an illustration, CERS2 utilizes long-chain C20- to C26-fatty acyl-CoAs, CERS4 uses C18- and C20-fatty acyl-CoAs, CERS5 is specific for palmitate (C16:0), and CERS6 is specific for myristate (C14:0) and palmitic acid [[66], [67], [68]]. The significance of the biophysical, molecular, and biological characteristics of the different ceramide species is a matter of intensive research. Currently available knowledge indicates clearly that ceramides can substantially influence pathophysiological processes in inflammation and cancer in a chain length-dependent manner (reviewed in [69]). We found increased levels of *CERK,* which encodes ceramide kinase to yield C1P. There are also complex interrelations between ceramide and sphingomyelin based on enhanced *SGMS1* and *SMPD2*, respectively. By adding the increased expression of *PPAP2C* (encoding the lipid phosphatase LPP2) the combined results indicate a shift from S1P and S1P/S1PR axis to ceramide/C1P-driven biological outcomes as well as an attenuation of LPA signaling in the *immune low* subgroup (Fig. 13). Furthermore, the enhanced expression of *LPAR2* and *LPAR3*, but none of *S1PRs,* was characteristic for the *immune low* tumor subtype. It is important to note that, according to our results, all of these genes are expressed by a variety of ovarian cancer cell lines as demonstrated in Fig. S3.

We were surprised to discover that the overall profile for *immune high* tumors was very different to what was seen in the *immune low* subgroup (Fig. 13). In *immune high* tumors we found increased levels of *SMPD1* encoding acidic sphingomyelinase that converts sphingomyelin to ceramide; *NAAA* encoding ceramidase that converts ceramide to sphingosine; *SPHK1,* a kinase that synthesizes S1P; *ENPP2* that encodes autotaxin, which produces LPA in the extracellular space; and *PPAP2A* encoding the lipid phosphatase LPP1, which, upon translocation to the plasma membrane, preferentially dephosphorylates LPA [70]. When the described aberrations are superimposed on the interconnected lipid — enzyme network covering the sphingomyelin/salvage pathway, we see a clear shift towards S1P and S1P/S1PR as well as LPA and LPA/LPAR axes (Fig. 13). Regarding the receptors for lipid mediators, enhanced expression in *immune high* tumors was detected for *S1PR1*, *S1PR4, S1PR5*, and for *LPAR1* and *LPAR5*. We also identified *CD1B* and *CD1D* encoding MHC-like lipid-presenting molecules [54] as distinguishing molecules of this subgroup. Overall, the results identify fundamental differences in cellular and molecular mechanisms attributed to the sphingolipid and lysophosphatidate/LPA machinery and propose the specific checkpoints being characteristic of either *immune high* or *immune low* tumor subtypes.

Previous reports suggested that altered LPA metabolism affects the pathobiology of ovarian cancer. It is important to note that LPA is present in elevated concentrations in plasma and ascites of ovarian cancer patients and other malignant effusions and is considered as a potential diagnostic marker for this disease ([71], reviewed in [72,73]). Although the precise mechanisms linking LPA and proliferation of tumor cells are not fully understood, a recent study has implicated the LPA/LPAR2 signaling in promotion of glucose metabolism in cancer cells, which in turn triggers ovarian cancer cell proliferation [74]. Interestingly, in our study *LPAR2* was shown to be a marker of the *immune low* tumor subtype. Another study showed that autotaxin, an LPA-producing enzyme, is highly secreted from ovarian cancer stem cells, and pharmacological inhibition of autotaxin decreases LPA-driven pro-tumorigenic potential of cancer stem cells and drug resistance [75]. In our study enhanced expression of *ENPP2* encoding autotaxin was detected in the *immune high* subgroup. Considering that *LPAR5* (and, to a lesser extent, *LPAR6*) in our study showed a positive association with leukocyte marker *CD45*, the ENPP2/LPA/LPAR5-6 axis is likely involved in trafficking, survival and/or communication of immune cell subpopulations. This is compatible with the effects of ATX inhibition, which decreases the expression of multiple cytokines and chemokines and thus the recruitment of leukocytes into the environment of the tumor [76,77]. In another study, using mouse models, increasing the expression of lipid phosphate phosphatase-1, which degrades extracellular LPA and decreases signaling downstream of LPAR1/2 receptors, decreases the abilities of aggressive cancer cells to produce tumors and metastases [78]. This is an interesting link to our findings where we show a close association between *PPAP2A* and *LPAR1* (found outside the immune markers-based cluster) in the *immune high* subgroup. This suggests an important role for the autotaxin/LPA/LPAR1 axis in cancer cell reprogramming.

The clustering-based patient stratification strategy clearly differentiates the *immune low/high* tumors and thereby could have clinical relevance by defining patient subgroups that might be more susceptible to the immune-based strategies. From a different perspective, the findings furthermore demonstrate that there are very special properties of tumor and/or tumor microenvironment, which are characteristic for *immune high* tumors. These properties support, attract, and keep on-site immune cell infiltrates. Enhancing immune cell recruitment to *immune low* tumors, e.g. by vaccines or modulation of S1P/S1PR and/or LPA/LPARs axes, might be beneficial for patients with a follow-up possibility to strengthen anti-tumoral immune responses. The limitation is the current inability to classify and characterize in more detail those patients, who do not clearly show either *immune low* or *immune high* phenotypes.

We found that the sphingolipid/lysophosphatidate/immune-based stratification into *immune low* and *immune high* subgroups is associated with the therapy response for the cohort of patients that we examined with significantly more responders found in *immune high* subgroup. Despite this, we detected responders in both *immune low* and *immune high* tumor subtypes. The mechanisms supporting the response to chemotherapy, however, could be different because of the activities of different “chemosensors”. Although speculative, the CERS/ceramide and/or SMPD/ceramide axes might contribute to apoptosis and chemotherapy response in *immune low* patients. By contrast, in *immune high* patients, the mechanisms are even more multifaceted and increased immunogenic cell death and reprogramming of the immunosuppressive microenvironment could be dominant.

We applied gene-clustering algorithms to evaluate functional connectivity among sphingolipid- and immune-related genes and found functional relationships characteristic for a particular cell type. Important additive information regarding potential sphingolipid-related mechanisms is based on identification of a close association between expression levels of *S1PR4* and *CD3E*. This supports the hypothesis of their co-expression and functional connectivity. Thus, among all potential S1P/S1PR axes, the S1P/S1PR4 axis appears particularly relevant for T-cell infiltrates in *immune high* ovarian tumors and supports the role of S1P signaling in T-cell trafficking and/or survival. This discovery acts as starting point to clarify the functionality of S1PR4 and the contribution of S1P/S1PR4 axis to the pathobiology of ovarian cancer. It is also important to note that S1PR4 is highly and specifically expressed on immune cells. Our meta-analysis corroborates that S1PR4 expression is restricted to leukocytes and that the T-cell subsets are within the top 10 cell types showing the strongest expression. S1PR4 is the least studied among all S1PRs. Its impact on T cells and generally on immune cell biology remains largely unknown. Thus far, the impact of S1PR4 was shown for plasmacytoid dendritic cells in the context of their differentiation and activation [79] and for neutrophils recruitment [80]. Of note, S1PR4 small molecule agonists with selectivity over the other S1PR family members are available [81,82]. The agonists could be used in cellular (T-cell-based) functional assays to elucidate the receptor functions.

Within the Module II of MuSiCO, we show the prognostic relevance of the self-designed sphingolipid/lysophosphatidate/immune-associated 38/8-gene signature for patients with primary serous ovarian carcinoma. From this, we propose a novel survival model for patient risk assessment as well as potential biomarkers/targeting strategies. At model validation, we identified that the combined model (clinical and profiling-derived variables) had the highest predictive accuracy with respect to OS. This once again emphasizes the interconnection between the sphingolipid and immune system in ovarian cancer. The applied modeling algorithm serves as novel stratification strategy and it is characterized by high performance in respect of prognostic accuracy and discriminative ability [49]. By the integrated model validation stage we can compare individual models within one study as well as between independent studies and laboratories based on the defined parameter set (PEV, c-index, and *p*-value) and not on a *p*-value alone. This validation approach is robust and guards against falsely identifying any relevance of expression data sets derived from randomly selected gene sets. It is intentionally based on the “leave-one-out strategy” of cross-validation instead of an arbitrary single split of the data set in to a training and test set. In the latter situation using a relatively small number of events, much of the data needed to obtain more stable estimates would be wasted, and the results would be sensitive to the specific split. Our strategy enables the ranking of variables/genes within the model by their impact to the prognostic effect. Among the covariates within the survival model, *CD68*, *LPAR3*, *SMPD1*, *PPAP2B*, and *SMPD2* emerged as the most important gene variables for prognosis of overall survival. Dissection of the signature-associated pathways, upstream regulators and reconstruction of the sphingolipid/lysophosphatidate/immune-associated gene network emphasizes the complementary impact of each top gene to the overall prognostic effect of the survival model (Fig. 12).

Ovarian tumor tissue provides a heterogeneous environment composed of a variety of cell types in addition to cancer cells. The transcriptional level of a particular gene is the sum of expression levels from various cell types composing the tissue. To dissect this complexity, we implemented the usage of digital pathology. Within the exploratory study, we analyzed the staining patterns attributed to the profiling-derived top candidate markers within whole paraffin-embedded histological slides. This approach is well characterized and it is routinely used by clinical institutions for diagnostic estimations. The retrieved information could provide additional knowledge for understanding the pathobiology, keeping in mind the lack of information about the expression patterns of the vast majority of lipid-modifying/related molecules within diseased tissues. Herein, we focused and explored predominantly the observational findings highlighting similarities in expression patterns for CD68 and SMPD1 (also known as acid sphingomyelinase, ASMase, ASM) in macrophage/monocyte lineage cells within the ovarian cancer tissue. In this respect, it is interesting to note that the similarity in expression was observed for various subpopulations including elongated intra-tumoral macrophages and round-shaped phagocytes within the phagocytic islands. This in turn might suggest that CD68 and SMPD1 could have functional similarity in cells with different (patho)physiological tasks. These two molecules were among the top genes that contributed positively to the survival model. They are the markers of *immune high* tumor subtype and both belong to the Lysosome Pathway by PathCards database. Whereas *CD68* and *SMPD1* were separated by hierarchical clustering of the-whole-tissue expression data sets, we found an overlap in co-expressed genes in cells of monocyte/macrophage lineage. CD68 belongs to the family of lysosomal/endosomal-associated membrane proteins (LAMP) and is also known as LAMP4. Although CD68 is a well-known routinely used immunohistochemical marker of cells of monocyte/macrophage lineage, the function of CD68 in immunity, inflammation, and tumor biology is surprisingly poorly understood (reviewed in [83]). From its side, SMPD1 could have the potential to modulate various responses in several immune cell sub-populations; it is involved in multiple aspects of macrophage biology to accomplish their diverse functions (reviewed in [84]). SMPD1 facilitates the formation of endocytic vesicles through ceramide generation [85]. It also modulates the structure of lipid rafts and formation of ceramide-rich platforms within plasma membrane, which, in turn, modulates the functionality of cellular receptors [[86], [87], [88]]. A further linkage to macrophage functionality is based on SMPD1-controled secretion of pro-inflammatory cytokines. These play a role in both macrophage survival and apoptosis in response to microbial agents, as well as in host defense through SPMD1-mediated phagosome-lysosome fusion [89]. Also linked to immunological activities are secretory granules/secretory lysosomes, which contain perforin and granzymes. Significantly, a single *SMPD1* gene is responsible for generation of both secretory and lysosomal forms of SMPD1 [90]. Our results provide a rationale for testing the hypothesis that secretory SMPD1 could be used as a potential biomarker in ovarian cancer patients.

A prominent presence of immune infiltrates and/or an occurrence of certain breakpoints in the cellular sphingolipid system are common features of many cancer types. Is there a specificity regarding the sphingolipid/lysophosphatidate/immune signature-associated mechanisms identified herein? Strikingly, an alignment of the sphingolipid/lysophosphatidate/immune-associated signature-based profile with microarray data sets attributed to great variety of cancer types (over 600 neoplasm categories; over 21,000 arrays) revealed high specificity for ovarian carcinoma. The approach that we described could provide a novel strategy for estimating the signature specificity and assessing the singularity or commonality of disease mechanisms. Interestingly, there is a difference in specificity when low risk and high risk patient subgroups are assessed separately. Presumably, a high risk group might be associated with more aggressive pathological features, with aberrations at multiple levels and “undifferentiated” tumor mass, which lack the specific structure and functions of the original cell type. With such loss of cell identity, the signature-associated profile becomes more common within various tumor types, independent of their origin. Given the novelty of our approach, the question regarding the most critical genes within the signature, which are necessary and sufficient to achieve the specificity is still ambitious and challenging. This needs to be resolved in a separate study.

## 5 Conclusion

We implemented a systems biology approach to uncover the complexity and dynamic interactions within the sphingolipid and LPA signaling machinery as a biological system. This was shown to be integrated with the components of immune system and clinical data in the context of ovarian cancer. We next implemented the systems biology-derived knowledge for understanding the personalized aspects of ovarian cancer connected to disease progression, treatment responses, and disease phenotypes. Such knowledge provides better patient stratification, therapeutic target discovery, and potentially leads to improvements in personalized medicine and clinical outcomes. This systems biology-based concept, thereby, contributes to systems medicine. It is universal in the sense that it can be applied to any biologically relevant gene signature and any type of complex multifactorial disorder. The results that we report on the integration of the sphingolipid/lysophosphatidate axis and the on-site immune system can be used to accelerate research in diverse disciplines and provide personalized approaches to patient care.

## Author Contributions

Conception and design: DM, AM, MS, PZ, and GH. Experimental part, analysis, and interpretation of data: AM, DM, MS, FM, MZ, GH, MJ, PB, DB, and PZ. Writing – Original Draft: AM, GH and DM; Writing – Review & Editing: MS, FM, MJ, PZ, RZ, DCCT, DP, PB, DB, and SM. Sample collection and maintaining patient database: RZ, DCCT, AW, DP, GuH, EIB, JS, SL, IV, SM, and PB. All authors read and approved the final manuscript.

## Conflict of Interest

The authors declare that they have no conflict of interest.

## Appendix A Supplementary Data

Supplementary material Image 1
