# Supplementary material for: Interrelations of Sphingolipid and Lysophosphatidate Signaling with Immune System in Ovarian Cancer
Source: Comput Struct Biotechnol J. 2019 Apr 10;17:537–60. doi: 10.1016/j.csbj.2019.04.004 (PMC6479272; doi:10.1016/j.csbj.2019.04.004)
Supplement: Supplementary file 1 — Supplementary material [file mmc1.pdf]

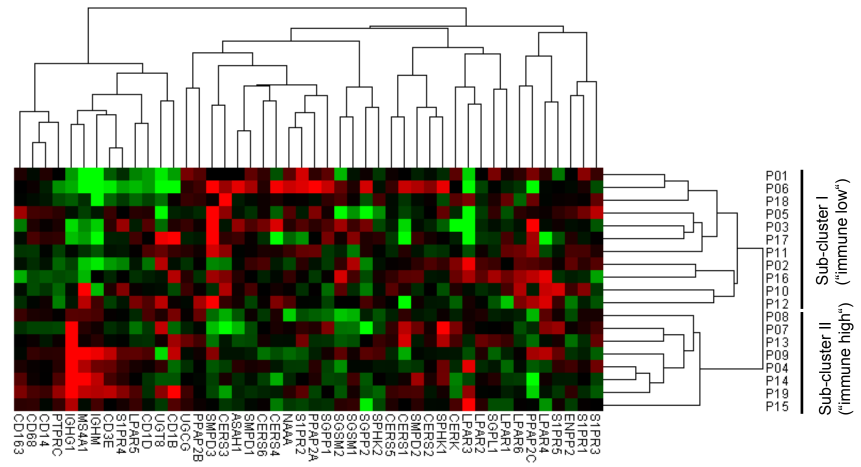

CD163
CD68
CD14
PTPRC
IGHG1
MS4A1
IGHM
CD3E
S1PR4
LPAR5
CD1D
UGT8
CD1B
UGCG
PPAP2B
SMPD3
CERS3
ASAH1
SMPD1
CERS6
CERS4
NAAA
S1PR2
PPAP2A
SGPP1
SGSM2
SGSM1
SGPP2
SPHK2
CERS5
CERS1
SMPD2
CERS2
SPHK1
CERK
LPAR3
LPAR2
SGPL1
LPAR1
LPAR6
PPAP2C
LPAR4
S1PR5
ENPP2
S1PR1
S1PR3
P01
P06
P18
P05
P03
P17
P11
P02
P16
P10
P12
P08
P07
P13
P09
P04
P14
P19
P15
Sub-cluster I
(“immune low“)
Sub-cluster II
(“immune high“)

Figure S1

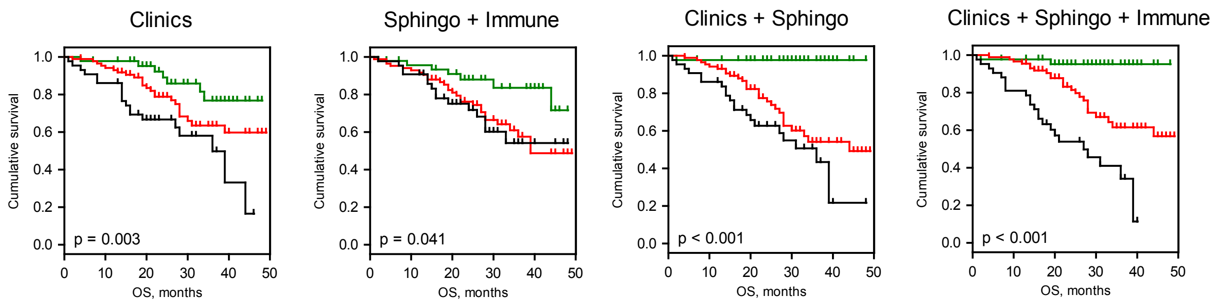
Clinics
Sphingo + Immune
Clinics + Sphingo
Clinics + Sphingo + Immune
Cumulative survival
1.0
0.8
0.6
0.4
0.2
0.0
0
10
20
30
40
50
OS, months
p = 0.003
p = 0.041
p < 0.001
p < 0.001

Figure S2

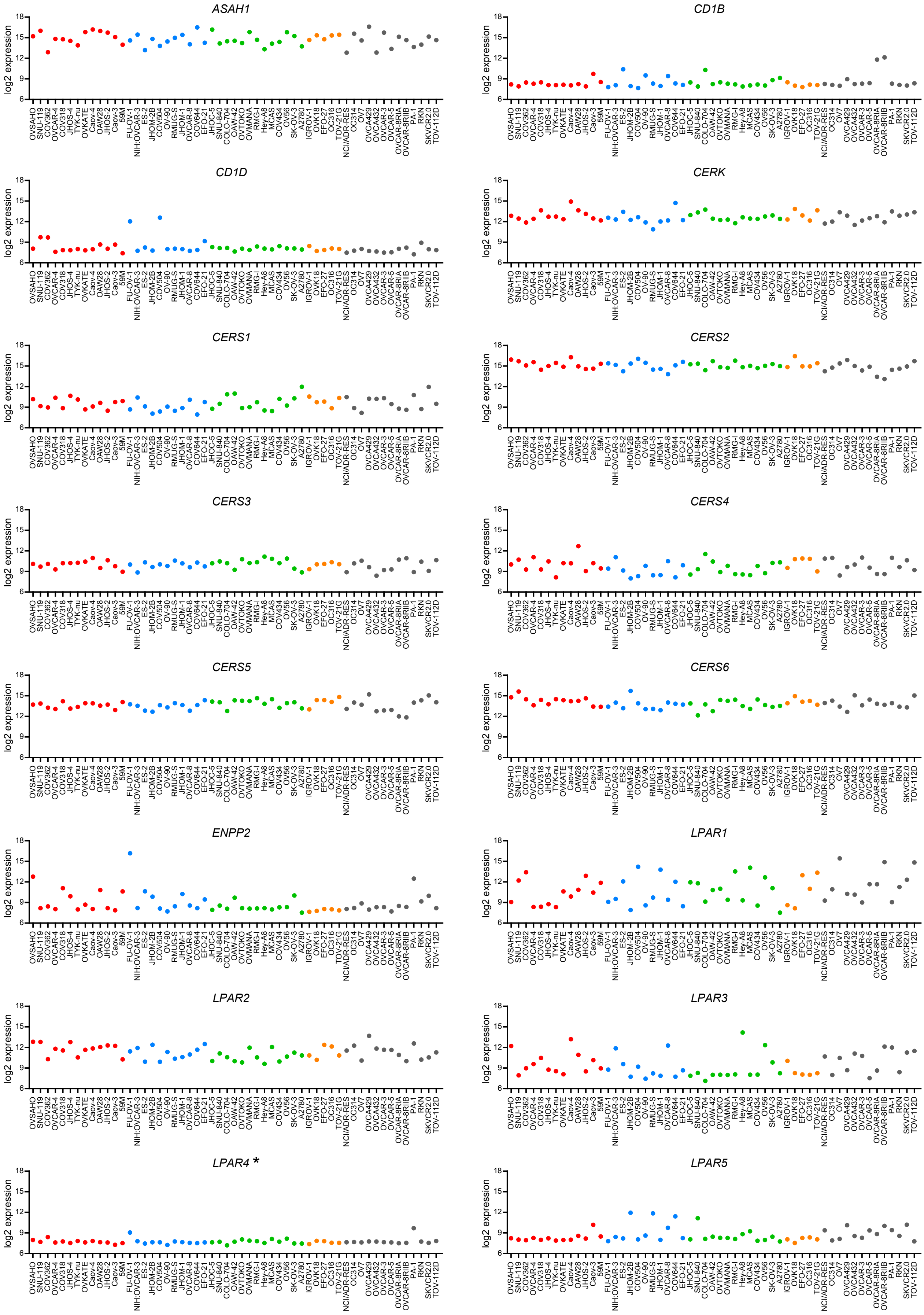

Figure S3_part1

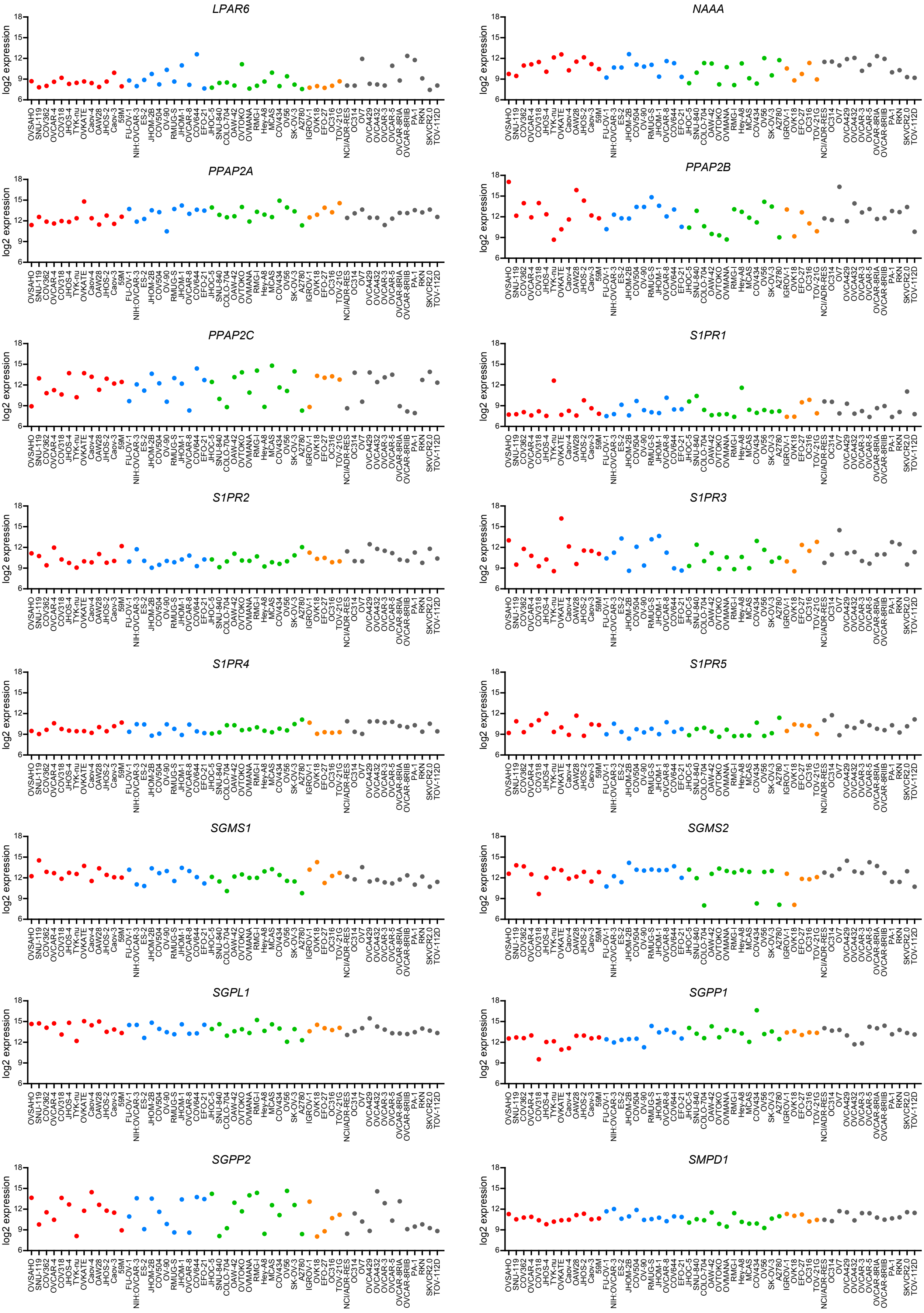

Figure S3_part 2

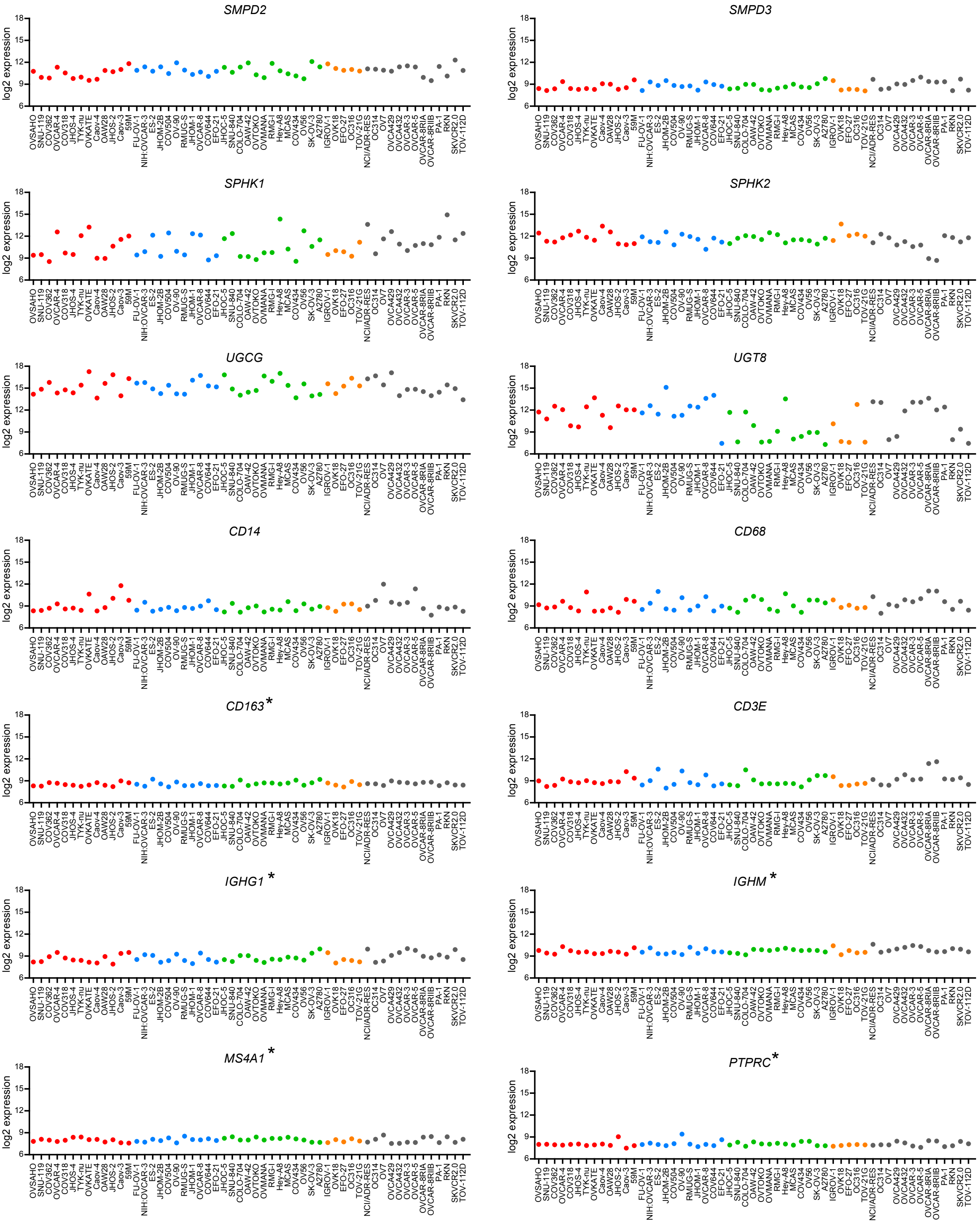

Figure S3_part 3

Table S1. Genes composing the sphingolipid/lysophosphatidate/immune-associated 38/8-gene signature. Gene symbol, synonyms, gene name, NCBI accession number, and short functional description are provided.

Symbol	Synonym	Name	NCBI accession number	Short functional description
**ASAH1**	**AC, ACDase, ASAH, PHP, PHP32, SMAPME**	N-acylsphingosine amidohydrolase (acid ceramidase) 1	NM_001127505.2	This gene encodes a member of the acid ceramidase family of proteins. Alternative splicing results in multiple transcript variants, at least one of which encodes a preproprotein that is proteolytically processed. Processing of this preproprotein generates alpha and beta subunits that heterodimerize to form the mature lysosomal enzyme, which catalyzes the degradation of ceramide into sphingosine and free fatty acid. This enzyme is overexpressed in multiple human cancers and may play a role in cancer progression. Mutations in this gene are associated with the lysosomal storage disorder, Farber lipogranulomatosis, and a neuromuscular disorder, spinal muscular atrophy with progressive myoclonic epilepsy. [provided by RefSeq, Oct 2015]
**CD1B**	**CD1, CD1A, R1**	CD1b molecule	NM_001764.2	This gene encodes a member of the CD1 family of transmembrane glycoproteins, which are structurally related to the major histocompatibility complex (MHC) proteins and form heterodimers with beta-2-microglobulin. The CD1 proteins mediate the presentation of primarily lipid and glycolipid antigens of self or microbial origin to T cells. The human genome contains five CD1 family genes organized in a cluster on chromosome 1. The CD1 family members are thought to differ in their cellular localization and specificity for particular lipid ligands. The protein encoded by this gene localizes to late endosomes and lysosomes via a tyrosine-based motif in the cytoplasmic tail, and requires vesicular acidification to bind lipid antigens. [provided by RefSeq, Jul 2008]
**CD1D**	**CD1A, R3, R3G1**	CD1d molecule	NM_001319145.1	This gene encodes a divergent member of the CD1 family of transmembrane glycoproteins, which are structurally related to the major histocompatibility complex (MHC) proteins and form heterodimers with beta-2-microglobulin. The CD1 proteins mediate the presentation of primarily lipid and glycolipid antigens of self or microbial origin to T cells. The human genome contains five CD1 family genes organized in a cluster on chromosome 1. The CD1 family members are thought to differ in their cellular localization and specificity for particular lipid ligands. The protein encoded by this gene localizes to late endosomes and lysosomes via a tyrosine-based motif in the cytoplasmic tail. Two transcript variants encoding different isoforms have been found for this gene. [provided by RefSeq, Jan 2016]
**CERK**	**LK4, dA59H18.2, dA59H18.3, hCERK**	ceramide kinase	NM_022766.5	CERK converts ceramide to ceramide 1-phosphate (C1P), a sphingolipid metabolite. Both CERK and C1P have been implicated in various cellular processes, including proliferation, apoptosis, phagocytosis, and inflammation (Kim et al., 2006 [PubMed 16488390]).[supplied by OMIM, Mar 2008]
**CERS1**	**EPM8, LAG1, LASS1, UOG1**	ceramide synthase 1	NM_001290265.1	This gene encodes a ceramide synthase enzyme, which catalyzes the synthesis of ceramide, the hydrophobic moiety of sphingolipids. The encoded enzyme synthesizes 18-carbon (C18) ceramide in brain neurons. Elevated expression of this gene may be associated with increased longevity, while decreased expression of this gene may be associated with myoclonus epilepsy with dementia in human patients. This protein is transcribed from a monocistronic mRNA as well as a bicistronic mRNA, which also encodes growth differentiation factor 1. [provided by RefSeq, Jul 2016]
**CERS2**	**L3, LASS2, SP260, TMSG1**	ceramide synthase 2	NM_022075.4	This gene encodes a protein that has sequence similarity to yeast longevity assurance gene 1. Mutation or overexpression of the related gene in yeast has been shown to alter yeast lifespan. The human protein may play a role in the regulation of cell growth. Alternatively spliced transcript variants encoding the same protein have been described. [provided by RefSeq, Jul 2008]
**CERS3**	**ARCI9, LASS3**	ceramide synthase 3	NM_001290341.2	This gene is a member of the ceramide synthase family of genes. The ceramide synthase enzymes regulate sphingolipid synthesis by catalyzing the formation of ceramides from sphingoid base and acyl-coA substrates. This family member is involved in the synthesis of ceramides with ultra-long-chain acyl moieties (ULC-Cers), important to the epidermis in its role in creating a protective barrier from the environment. The protein encoded by this gene has also been implicated in modification of the lipid structures required for spermatogenesis. Mutations in this gene have been associated with male fertility defects, and epidermal defects, including ichthyosis. Alternative splicing results in multiple transcript variants encoding different isoforms. [provided by RefSeq, Aug 2015]
**CERS4**	**LASS4, Trh1**	ceramide synthase 4	NM_024552.2	RefSeq status: VALIDATED
**CERS5**	**LASS5, Trh4**	ceramide synthase 5	NM_001281731.1	This gene encodes a protein that belongs to the TLC (TRAM, LAG1 and CLN8 homology domains) family of proteins. The encoded protein functions in the synthesis of ceramide, a lipid molecule that is involved in a several cellular signaling pathways. Alternate splicing results in multiple transcript variants. [provided by RefSeq, Aug 2013]
**CERS6**	**CERS5, LASS6**	ceramide synthase 6	NM_001256126.1	RefSeq status: VALIDATED
**ENPP2**	**ATX, ATX-X, AUTOTAXIN, LysoPLD, NPP2, PD-IALPHA, PDNP2**	ectonucleotide pyrophosphatase/phosphodiesterase 2	NM_001040092.2	The protein encoded by this gene functions as both a phosphodiesterase, which cleaves phosphodiester bonds at the 5' end of oligonucleotides, and a phospholipase, which catalyzes production of lysophosphatidic acid (LPA) in extracellular fluids. LPA evokes growth factor-like responses including stimulation of cell proliferation and chemotaxis. This gene product stimulates the motility of tumor cells and has angiogenic properties, and its expression is upregulated in several kinds of carcinomas. The gene product is secreted and further processed to make the biologically active form. Several alternatively spliced transcript variants encoding different isoforms have been identified. [provided by RefSeq, Aug 2008]

**LPAR1**	**EDG2, GPR26, Gpcr26, LPA1, Mrec1.3, VZG1, edg-2, rec.1.3, vzg-1**	lysophosphatidic acid receptor 1	NM_001401.3	The integral membrane protein encoded by this gene is a lysophosphatidic acid (LPA) receptor from a group known as EDG receptors. These receptors are members of the G protein-coupled receptor superfamily. Utilized by LPA for cell signaling, EDG receptors mediate diverse biologic functions, including proliferation, platelet aggregation, smooth muscle contraction, inhibition of neuroblastoma cell differentiation, chemotaxis, and tumor cell invasion. Two transcript variants encoding the same protein have been identified for this gene [provided by RefSeq, Jul 2008]
**LPAR2**	**EDG-4, EDG4, LPA-2, LPA2**	lysophosphatidic acid receptor 2	NM_004720.5	This gene encodes a member of family I of the G protein-coupled receptors, as well as the EDG family of proteins. This protein functions as a lysophosphatidic acid (LPA) receptor and contributes to Ca2+ mobilization, a critical cellular response to LPA in cells, through association with Gi and Gq proteins. An alternative splice variant has been described but its full length sequence has not been determined. [provided by RefSeq, Jul 2008]
**LPAR3**	**EDG7, Edg-7, GPCR, HOFNH30, LP-A3, LPA3, RP4-678I3**	lysophosphatidic acid receptor 3	NM_012152.2	This gene encodes a member of the G protein-coupled receptor family, as well as the EDG family of proteins. This protein functions as a cellular receptor for lysophosphatidic acid and mediates lysophosphatidic acid-evoked calcium mobilization. This receptor couples predominantly to G(q/11) alpha proteins. [provided by RefSeq, Jul 2008]
**LPAR4**	**GPR23, LPA4, P2RY9, P2Y5-LIKE, P2Y9**	lysophosphatidic acid receptor 4	NM_001278000.1	This gene encodes a member of the lysophosphatidic acid receptor family. It may also be related to the P2Y receptors, a family of receptors that bind purine and pyrimidine nucleotides and are coupled to G proteins. The encoded protein may play a role in monocytic differentiation. [provided by RefSeq, Feb 2009]
**LPAR5**	**GPR92, GPR93, KPG_010, LPA5**	lysophosphatidic acid receptor 5	NM_001142961.1	This gene encodes a member of the rhodopsin class of G protein-coupled transmembrane receptors. This protein transmits extracellular signals from lysophosphatidic acid to cells through heterotrimeric G proteins and mediates numerous cellular processes. Many G protein receptors serve as targets for pharmaceutical drugs. Transcript variants of this gene have been described.[provided by RefSeq, Dec 2008]
**LPAR6**	**ARWH1, HYPT8, LAH3, P2RY5, P2Y5**	lysophosphatidic acid receptor 6	NM_001162497.1	The protein encoded by this gene belongs to the family of G-protein coupled receptors, that are preferentially activated by adenosine and uridine nucleotides. This gene aligns with an internal intron of the retinoblastoma susceptibility gene in the reverse orientation. Alternative splicing results in multiple transcript variants. [provided by RefSeq, Jun 2009]
**NAAA**	**ASAHL, PLT**	N-acylethanolamine acid amidase	NM_001042402.1	This gene encodes an N-acylethanolamine-hydrolyzingenzyme which is highly similar to acid ceramidase. Multiple transcript variants encoding different isoforms have been found for this gene. [provided by RefSeq, Jul 2008
**PLPP1 (PPAP2A)**	**LLP1a, LPP1, PAP-2a, PAP2, PPAP2A**	phospholipid phosphatase 1	NM_003711.3	The protein encoded by this gene is a member of the phosphatidic acid phosphatase (PAP) family. PAPs convert phosphatidic acid to diacylglycerol, and function in synthesis of glycerolipids and in phospholipase D-mediated signal transduction. This enzyme is an integral membrane glycoprotein that plays a role in the hydrolysis and uptake of lipids from extracellular space. Alternate splicing results in multiple transcript variants of this gene. [provided by RefSeq, May 2013]
**PLPP3 (PPAP2B)**	**Dri42, LPP3, PAP2B, PPAP2B, VCIP**	phospholipid phosphatase 3	NM_003713.4	The protein encoded by this gene is a member of the phosphatidic acid phosphatase (PAP) family. PAPs convert phosphatidic acid to diacylglycerol, and function in de novo synthesis of glycerolipids as well as in receptor-activated signal transduction mediated by phospholipase D. This protein is a membrane glycoprotein localized at the cell plasma membrane. It has been shown to actively hydrolyze extracellular lysophosphatidic acid and short-chain phosphatidic acid. The expression of this gene is found to be enhanced by epidermal growth factor in Hela cells. [provided by RefSeq, Mar 2010]
**PLPP2 (PPAP2C)**	**LPP2, PAP-2c, PAP2-g, PPAP2C**	phospholipid phosphatase 2	NM_003712.3	The protein encoded by this gene is a member of the phosphatidic acid phosphatase (PAP) family. PAPs convert phosphatidic acid to diacylglycerol, and function in de novo synthesis of glycerolipids as well as in receptor-activated signal transduction mediated by phospholipase D. This protein is similar to phosphatidic acid phosphatase type 2A (PPAP2A) and type 2B (PPAP2B). All three proteins contain 6 transmembrane regions, and a consensus N-glycosylation site. This protein has been shown to possess membrane associated PAP activity. Three alternatively spliced transcript variants encoding distinct isoforms have been reported. [provided by RefSeq, Jul 2008
**S1PR1**	**CD363, CHEDG1, D1S3362, ECGF1, EDG-1, EDG1, S1P1**	sphingosine-1-phosphate receptor 1	NM_001320730.1	The protein encoded by this gene is structurally similar to G protein-coupled receptors and is highly expressed in endothelial cells. It binds the ligand sphingosine-1-phosphate with high affinity and high specificity, and suggested to be involved in the processes that regulate the differentiation of endothelial cells. Activation of this receptor induces cell-cell adhesion. Alternative splicing results in multiple transcript variants. [provided by RefSeq, Mar 2016]
**S1PR2**	**AGR16, DFNB68, EDG-5, EDG5, Gpcr13, H218, LPB2, S1P2**	sphingosine-1-phosphate receptor 2	NM_004230.3	This gene encodes a member of the G protein-coupled receptors, as well as the EDG family of proteins. The encoded protein is a receptor for sphingosine 1-phosphate, which participates in cell proliferation, survival, and transcriptional activation. Defects in this gene have been associated with congenital profound deafness. [provided by RefSeq, Mar 2016]
**S1PR3**	**EDG-3, EDG3, LPB3, S1P3**	sphingosine-1-phosphate receptor 3	NM_005226.3	This gene encodes a member of the EDG family of receptors, which are G protein-coupled receptors. This protein has been identified as a functional receptor for sphingosine 1-phosphate and likely contributes to the regulation of angiogenesis and vascular endothelial cell function. [provided by RefSeq, Jul 2008]
**S1PR4**	**EDG6, LPC1, S1P4, SLP4**	sphingosine-1-phosphate receptor 4	NM_003775.3	This gene is a member of the endothelial differentiation, G-protein-coupled (EDG)) receptor gene family. EDG receptors bind lysophospholipids or lysosphingolipids as ligands, and are involved in cell signalling in many different cell types. This EDG receptor gene is intronless and is specifically expressed in the lymphoid tissue. [provided by RefSeq, Jul 2008]

**S1PR5**	**EDG8, Edg-8, S1P5, SPPR-1, SPPR-2**	sphingosine-1-phosphate receptor 5	NM_001166215.1	The lysosphingolipid sphingosine 1-phosphate (S1P) regulates cell proliferation, apoptosis, motility, and neurite retraction. Its actions may be both intracellular as a second messenger and extracellular as a receptor ligand. S1P and the structurally related lysolipid mediator lysophosphatidic acid (LPA) signal cells through a set of G protein-coupled receptors known as EDG receptors. Some EDG receptors (e.g., EDG1; MIM 601974) are S1P receptors; others (e.g., EDG2; MIM 602282) are LPA receptors.[supplied by OMIM, Mar 2008]
**SGMS1**	**MOB, MOB1, SMS1, TMEM23, hmob33**	sphingomyelin synthase 1	NM_147156.3	The protein encoded by this gene is predicted to be a five-pass transmembrane protein. This gene may be predominately expressed in brain. [provided by RefSeq, Jul 2008]
**SGMS2**	**SMS2**	sphingomyelin synthase 2	NM_001136257.1	Sphingomyelin, a major component of cell and Golgi membranes, is made by the transfer of phosphocholine from phosphatidylcholine onto ceramide, with diacylglycerol as a side product. The protein encoded by this gene is an enzyme that catalyzes this reaction primarily at the cell membrane. The synthesis is reversible, and this enzyme can catalyze the reaction in either direction. The encoded protein is required for cell growth. Three transcript variants encoding the same protein have been found for this gene. There is evidence for more variants, but the full-length nature of their transcripts has not been determined.[provided by RefSeq, Oct 2008]
**SGPL1**	**S1PL, SPL**	sphingosine-1-phosphate lyase 1	NM_003901.3	RefSeq status: VALIDATED
**SGPP1**	**SPPase1**	sphingosine-1-phosphate phosphatase 1	NM_030791.3	Sphingosine-1-phosphate (S1P) is a bioactive sphingolipid metabolite that regulates diverse biologic processes. SGPP1 catalyzes the degradation of S1P via salvage and recycling of sphingosine into long-chain ceramides (Mandala et al., 2000 [PubMed 10859351]; Le Stunff et al., 2007 [PubMed 17895250]).[supplied by OMIM, Jun 2009]
**SGPP2**	**SPP2, SPPase2**	sphingosine-1-phosphate phosphatase 2	NM_001320833.1	The protein encoded by this gene is a transmembrane protein that degrades the bioactive signaling molecule sphingosine 1-phosphate. The encoded protein is induced during inflammatory responses and has been shown to be downregulated by the microRNA-31 tumor suppressor. Alternative splice variants encoding different isoforms have been found for this gene. [provided by RefSeq, Mar 2016]
**SMPD1**	**ASM, ASMASE, NPD**	sphingomyelin phosphodiesterase 1	NM_000543.4	The protein encoded by this gene is a lysosomal acid sphingomyelinase that converts sphingomyelin to ceramide. The encoded protein also has phospholipase C activity. Defects in this gene are a cause of Niemann-Pick disease type A (NPA) and Niemann-Pick disease type B (NPB). Multiple transcript variants encoding different isoforms have been identified. [provided by RefSeq, Jul 2010]
**SMPD2**	**ISC1, NSMASE, NSMASE1**	sphingomyelin phosphodiesterase 2	NM_003080.2	This gene encodes a protein which was initially identified as a sphingomyelinase based on sequence similarity between bacterial sphingomyelinases and a yeast protein. Subsequent studies showed that its biological function is less likely to be as a sphingomyelinase and instead as a lysophospholipase. [provided by RefSeq, Oct 2009]
**SMPD3**	**NSMASE2**	sphingomyelin phosphodiesterase 3	NM_018667.3	RefSeq status: VALIDATED
**SPHK1**	**SPHK**	sphingosine kinase 1	NM_001142601.1	The protein encoded by this gene catalyzes the phosphorylation of sphingosine to form sphingosine-1-phosphate (S1P), a lipid mediator with both intra- and extracellular functions. Intracellularly, S1P regulates proliferation and survival, and extracellularly, it i[illegible] a ligand for cell surface G protein-coupled receptors. This protein, and its product S1P, play a key role in TNF-alpha signaling and the NF-kappa-B activation pathway important in inflammatory, antiapoptotic, and immune processes. Alternatively spliced transcript variants encoding different isoforms have been found for this gene. [provided by RefSeq, Sep 2011]
**SPHK2**	**SK 2, SK-2, SPK 2, SPK-2**	sphingosine kinase 2	NM_001204158.2	This gene encodes one of two sphingosine kinase isozymes that catalyze the phosphorylation of sphingosine into sphingosine 1-phosphate. Sphingosine 1-phosphate mediates many cellular processes including migration, proliferation and apoptosis, and also plays a role in several types of cancer by promoting angiogenesis and tumorigenesis. The encoded protein may play a role in breast cancer proliferation and chemoresistance. Alternatively spliced transcript variants encoding multiple isoforms have been observed for this gene. [provided by RefSeq, Aug 2011]
**UGCG**	**GCS, GLCT1**	UDP-glucose ceramide glucosyltransferase	NM_003358.2	This gene encodes an enzyme that catalyzes the first glycosylation step in the biosynthesis of glycosphingolipids, which are membrane components containing lipid and sugar moieties. The product of this reaction is glucosylceramide, which is the core structure of many glycosphingolipids. [provided by RefSeq, Dec 2014]
**UGT8**	**CGT, UGT4**	UDP glycosyltransferase 8	NM_001128174.2	The protein encoded by this gene belongs to the UDP-glycosyltransferase family. It catalyzes the transfer of galactose to ceramide, a key enzymatic step in the biosynthesis of galactocerebrosides, which are abundant sphingolipids of the myelin membrane of the central and peripheral nervous systems. Alternatively spliced transcript variants have been found for this gene. [provided by RefSeq, Sep 2011]
**CD14**		CD14 molecule	NM_000591.3	The protein encoded by this gene is a surface antigen that is preferentially expressed on monocytes/macrophages. It cooperates with other proteins to mediate the innate immune response to bacterial lipopolysaccharide. Alternative splicing results in multiple transcript variants encoding the same protein. [provided by RefSeq, Mar 2010]

**CD68**	**GP110, LAMP4, SCARD1**	CD68 molecule	NM_001040059.1	This gene encodes a 110-kD transmembrane glycoprotein that is highly expressed by human monocytes and tissue macrophages. It is a member of the lysosomal/endosomal-associated membrane glycoprotein (LAMP) family. The protein primarily localizes to lysosomes and endosomes with a smaller fraction circulating to the cell surface. It is a type I integral membrane protein with a heavily glycosylated extracellular domain and binds to tissue- and organ-specific lectins or selectins. The protein is also a member of the scavenger receptor family. Scavenger receptors typically function to clear cellular debris, promote phagocytosis, and mediate the recruitment and activation of macrophages. Alternative splicing results in multiple transcripts encoding different isoforms. [provided by RefSeq, Jul 2008]
**CD163**	**M130, MM130, SCARI1**	CD163 molecule	NM_004244.5	The protein encoded by this gene is a member of the scavenger receptor cysteine-rich (SRCR) superfamily, and is exclusively expressed in monocytes and macrophages. It functions as an acute phase-regulated receptor involved in the clearance and endocytosis of hemoglobin/haptoglobin complexes by macrophages, and may thereby protect tissues from free hemoglobin-mediated oxidative damage. This protein may also function as an innate immune sensor for bacteria and inducer of local inflammation. Alternatively spliced transcript variants encoding different isoforms have been described for this gene. [provided by RefSeq, Aug 2011]
**CD3E**	**IMD18, T3E, TCRE**	CD3e molecule	NM_000733.3	The protein encoded by this gene is the CD3-epsilon polypeptide, which together with CD3-gamma, -delta and -zeta, and the T-cell receptor alpha/beta and gamma/delta heterodimers, forms the T-cell receptor-CD3 complex. This complex plays an important role in coupling antigen recognition to several intracellular signal-transduction pathways. The genes encoding the epsilon, gamma and delta polypeptides are located in the same cluster on chromosome 11. The epsilon polypeptide plays an essential role in T-cell development. Defects in this gene cause immunodeficiency. This gene has also been linked to a susceptibility to type I diabetes in women. [provided by RefSeq, Jul 2008]
**IGHG1**		immunoglobulin heavy constant gamma 1		RefSeq status: VALIDATED
**IGHM**	**AGM1, MU, VH**	immunoglobulin heavy constant mu		Immunoglobulins (Ig) are the antigen recognition molecules of B cells. An Ig molecule is made up of 2 identical heavy chains and 2 identical light chains (see MIM 147200) joined by disulfide bonds so that each heavy chain is linked to a light chain and the 2 heavy chains are linked together. Each Ig heavy chain has an N-terminal variable (V) region containing the antigen-binding site and a C-terminal constant (C) region, encoded by an individual C region gene, that determines the isotype of the antibody and provides effector or signaling functions. The heavy chain V region is encoded by 1 each of 3 types of genes: V genes (see MIM 147070), joining (J) genes (see MIM 147010), and diversity (D) genes (see MIM 146910). The C region genes are clustered downstream of the V region genes within the heavy chain locus on chromosome 14. The IGHM gene encodes the C region of the mu heavy chain, which defines the IgM isotype. Naive B cells express the transmembrane forms of IgM and IgD (see IGHD; MIM 1471770) on their surface. During an antibody response, activated B cells can switch to the expression of individual downstream heavy chain C region genes by a process of somatic recombination known as isotype switching. In addition, secreted Ig forms that act as antibodies can be produced by alternative RNA processing of the heavy chain C region sequences. Although the membrane forms of all Ig isotypes are monomeric, secreted IgM forms pentamers, and occasionally hexamers, in plasma (summary by Janeway et al., 2005).[supplied by OMIM, Aug 2010]
**MS4A1**	**B1, Bp35, CD20, CVID5, LEU-16, MS4A2, S7**	membrane spanning 4-domains A1	NM_021950.3	This gene encodes a member of the membrane-spanning 4A gene family. Members of this nascent protein family are characterized by common structural features and similar intron/exon splice boundaries and display unique expression patterns among hematopoietic cells and nonlymphoid tissues. This gene encodes a B-lymphocyte surface molecule which plays a role in the development and differentiation of B-cells into plasma cells. This family member is localized to 11q12, among a cluster of family members. Alternative splicing of this gene results in two transcript variants which encode the same protein. [provided by RefSeq, Jul 2008]
**PTPRC**	**B220, CD45, CD45R, GP180, L-CA, LCA, LY5, T200**	protein tyrosine phosphatase, receptor type C	NM_001267798.1	The protein encoded by this gene is a member of the protein tyrosine phosphatase (PTP) family. PTPs are known to be signaling molecules that regulate a variety of cellular processes including cell growth, differentiation, mitosis, and oncogenic transformation. This PTP contains an extracellular domain, a single transmembrane segment and two tandem intracytoplasmic catalytic domains, and thus is classified as a receptor type PTP. This PTP has been shown to be an essential regulator of T- and B-cell antigen receptor signaling. It functions through either direct interaction with components of the antigen receptor complexes, or by activating various Src family kinases required for the antigen receptor signaling. This PTP also suppresses JAK kinases, and thus functions as a regulator of cytokine receptor signaling. Alternatively spliced transcripts variants of this gene, which encode distinct isoforms, have been reported. [provided by RefSeq, Jun 2012]

Table S2. Correlation matrix. Correlation matrix for expression data sets includes Pearson's correlation coefficients, p-values and Bonferroni Holm-adjusted p-values. Statistically significant Bonferroni Holm-adjusted p-values are highlighted in bold. Color code for statistically significant co-reg*red*, correlation coefficient >0.5; *blue*, correlation coefficient ≤0.5.

	ASAH1	CD1B	CD1D	CERK	CERS1	CERS2	CERS3	CERS4	CERS5	CERS6	ENPP2	LPAR1	LPAR2	LPAR3	LPAR4
CD1B	0.218<br>0.004<br>1.000														
CD1D	0.181<br>0.017<br>1.000	0.290<br>< 0.001<br>0.086													
CERK	0.281<br>< 0.001<br>0.143	-0.039<br>0.612<br>1.000	0.188<br>0.013<br>1.000												
CERS1	-0.125<br>0.102<br>1.000	-0.210<br>0.006<br>1.000	0.004<br>0.962<br>1.000	0.132<br>0.083<br>1.000											
CERS2	0.131<br>0.085<br>1.000	-0.018<br>0.814<br>1.000	0.040<br>0.599<br>1.000	**0.416**<br>< 0.001<br>**< 0.001**	0.047<br>0.535<br>1.000										
CERS3	0.085<br>0.263<br>1.000	0.066<br>0.387<br>1.000	0.149<br>0.050<br>1.000	0.240<br>0.002<br>1.000	0.000<br>0.999<br>1.000	0.162<br>0.033<br>1.000									
CERS4	0.069<br>0.364<br>1.000	-0.119<br>0.118<br>1.000	0.006<br>0.937<br>1.000	0.177<br>0.020<br>1.000	**0.331**<br>< 0.001<br>**0.007**	**0.314**<br>< 0.001<br>**0.021**	0.104<br>0.174<br>1.000								
CERS5	0.183<br>0.016<br>1.000	-0.052<br>0.497<br>1.000	0.047<br>0.535<br>1.000	**0.475**<br>< 0.001<br>**< 0.001**	0.138<br>0.069<br>1.000	**0.524**<br>< 0.001<br>**< 0.001**	0.115<br>0.133<br>1.000	**0.405**<br>< 0.001<br>**< 0.001**							
CERS6	0.090<br>0.241<br>1.000	-0.223<br>0.003<br>1.000	-0.083<br>0.277<br>1.000	**0.319**<br>< 0.001<br>**0.016**	0.096<br>0.210<br>1.000	**0.364**<br>< 0.001<br>**< 0.001**	0.145<br>0.057<br>1.000	0.272<br>< 0.001<br>0.221	**0.558**<br>< 0.001<br>**< 0.001**						
ENPP2	0.265<br>< 0.001<br>0.320	**0.307**<br>< 0.001<br>**0.032**	**0.393**<br>< 0.001<br>**< 0.001**	0.172<br>0.023<br>1.000	-0.070<br>0.362<br>1.000	0.037<br>0.626<br>1.000	0.115<br>0.131<br>1.000	0.109<br>0.152<br>1.000	0.060<br>0.432<br>1.000	-0.237<br>0.002<br>1.000					
LPAR1	0.289<br>< 0.001<br>0.092	0.272<br>< 0.001<br>0.226	0.066<br>0.390<br>1.000	0.151<br>0.048<br>1.000	-0.028<br>0.716<br>1.000	-0.043<br>0.578<br>1.000	0.034<br>0.660<br>1.000	-0.057<br>0.460<br>1.000	0.007<br>0.922<br>1.000	-0.036<br>0.634<br>1.000	0.279<br>< 0.001<br>0.156				
LPAR2	0.032<br>0.676<br>1.000	-0.051<br>0.508<br>1.000	0.091<br>0.235<br>1.000	**0.322**<br>< 0.001<br>**0.013**	0.292<br>< 0.001<br>0.080	**0.397**<br>< 0.001<br>**< 0.001**	0.120<br>0.115<br>1.000	0.281<br>< 0.001<br>0.141	**0.377**<br>< 0.001<br>**< 0.001**	**0.300**<br>< 0.001<br>**0.048**	-0.052<br>0.498<br>1.000	-0.063<br>0.407<br>1.000			
LPAR3	-0.255<br>< 0.001<br>0.524	-0.210<br>0.005<br>1.000	-0.197<br>0.009<br>1.000	0.104<br>0.174<br>1.000	0.203<br>0.007<br>1.000	0.103<br>0.179<br>1.000	0.109<br>0.154<br>1.000	0.120<br>0.117<br>1.000	0.091<br>0.236<br>1.000	0.203<br>0.007<br>1.000	-0.224<br>0.003<br>1.000	-0.202<br>0.008<br>1.000	**0.322**<br>< 0.001<br>**0.013**		
LPAR4	0.162<br>0.033<br>1.000	0.131<br>0.087<br>1.000	0.139<br>0.068<br>1.000	**0.323**<br>< 0.001<br>**0.013**	0.122<br>0.108<br>1.000	0.046<br>0.549<br>1.000	-0.043<br>0.575<br>1.000	0.145<br>0.057<br>1.000	0.149<br>0.051<br>1.000	0.049<br>0.519<br>1.000	0.174<br>0.022<br>1.000	**0.397**<br>< 0.001<br>**< 0.001**	-0.054<br>0.484<br>1.000	-0.120<br>0.117<br>1.000	
LPAR5	0.256<br>< 0.001<br>0.493	**0.424**<br>< 0.001<br>**< 0.001**	**0.444**<br>< 0.001<br>**< 0.001**	0.171<br>0.024<br>1.000	-0.135<br>0.076<br>1.000	0.026<br>0.733<br>1.000	0.260<br>< 0.001<br>0.408	0.006<br>0.937<br>1.000	-0.002<br>0.983<br>1.000	-0.098<br>0.200<br>1.000	**0.317**<br>< 0.001<br>**0.018**	0.180<br>0.018<br>1.000	0.049<br>0.518<br>1.000	-0.095<br>0.211<br>1.000	0.289<br>< 0.001<br>0.094
LPAR6	**0.375**<br>< 0.001<br>**< 0.001**	0.204<br>0.007<br>1.000	**0.434**<br>< 0.001<br>**< 0.001**	**0.423**<br>< 0.001<br>**< 0.001**	-0.066<br>0.390<br>1.000	0.244<br>0.001<br>0.879	0.159<br>0.036<br>1.000	0.168<br>0.027<br>1.000	0.202<br>0.008<br>1.000	0.045<br>0.555<br>1.000	**0.391**<br>< 0.001<br>**< 0.001**	0.210<br>0.006<br>1.000	0.140<br>0.067<br>1.000	-0.088<br>0.248<br>1.000	**0.413**<br>< 0.001<br>**< 0.001**
NAAA	**0.335**<br>< 0.001<br>**0.006**	**0.453**<br>< 0.001<br>**< 0.001**	0.258<br>< 0.001<br>0.438	0.133<br>0.080<br>1.000	-0.142<br>0.063<br>1.000	0.049<br>0.520<br>1.000	0.059<br>0.437<br>1.000	0.034<br>0.659<br>1.000	0.068<br>0.371<br>1.000	-0.050<br>0.512<br>1.000	0.198<br>0.009<br>1.000	0.253<br>< 0.001<br>0.573	-0.041<br>0.594<br>1.000	-0.180<br>0.018<br>1.000	**0.319**<br>< 0.001<br>**0.016**
PPAP2A	**0.360**<br>< 0.001<br>**< 0.001**	0.199<br>0.009<br>1.000	0.228<br>0.003<br>1.000	0.164<br>0.031<br>1.000	-0.026<br>0.738<br>1.000	0.065<br>0.398<br>1.000	0.038<br>0.620<br>1.000	0.077<br>0.311<br>1.000	0.142<br>0.062<br>1.000	-0.074<br>0.333<br>1.000	**0.538**<br>< 0.001<br>**< 0.001**	**0.534**<br>< 0.001<br>**< 0.001**	-0.215<br>0.005<br>1.000	**-0.351**<br>< 0.001<br>**0.002**	**0.494**<br>< 0.001<br>**< 0.001**
PPAP2B	0.101<br>0.185<br>1.000	0.025<br>0.745<br>1.000	0.050<br>0.515<br>1.000	0.188<br>0.013<br>1.000	0.260<br>< 0.001<br>0.411	0.160<br>0.035<br>1.000	0.049<br>0.521<br>1.000	0.199<br>0.009<br>1.000	0.228<br>0.003<br>1.000	0.064<br>0.401<br>1.000	0.289<br>< 0.001<br>0.090	0.277<br>< 0.001<br>0.178	0.049<br>0.519<br>1.000	-0.019<br>0.802<br>1.000	**0.320**<br>< 0.001<br>**0.015**
PPAP2C	0.218<br>0.004<br>1.000	-0.031<br>0.688<br>1.000	-0.055<br>0.469<br>1.000	0.033<br>0.669<br>1.000	-0.034<br>0.658<br>1.000	0.075<br>0.326<br>1.000	-0.012<br>0.878<br>1.000	-0.107<br>0.159<br>1.000	-0.095<br>0.215<br>1.000	-0.118<br>0.123<br>1.000	-0.044<br>0.562<br>1.000	-0.048<br>0.529<br>1.000	0.028<br>0.710<br>1.000	0.033<br>0.663<br>1.000	-0.215<br>0.005<br>1.000
S1PR1	**0.342**<br>< 0.001<br>**0.004**	0.184<br>0.015<br>1.000	**0.321**<br>< 0.001<br>**0.014**	0.214<br>0.005<br>1.000	0.066<br>0.390<br>1.000	0.011<br>0.889<br>1.000	0.055<br>0.472<br>1.000	0.116<br>0.129<br>1.000	0.108<br>0.158<br>1.000	-0.058<br>0.449<br>1.000	**0.593**<br>< 0.001<br>**< 0.001**	**0.500**<br>< 0.001<br>**< 0.001**	-0.016<br>0.833<br>1.000	-0.237<br>0.002<br>1.000	**0.443**<br>< 0.001<br>**< 0.001**
S1PR2	0.140<br>0.066<br>1.000	-0.023<br>0.768<br>1.000	-0.041<br>0.594<br>1.000	0.242<br>0.001<br>0.938	0.098<br>0.201<br>1.000	0.077<br>0.317<br>1.000	0.025<br>0.742<br>1.000	**0.352**<br>< 0.001<br>**0.002**	**0.400**<br>< 0.001<br>**< 0.001**	0.201<br>0.008<br>1.000	-0.002<br>0.977<br>1.000	0.199<br>0.009<br>1.000	0.223<br>0.003<br>1.000	-0.026<br>0.735<br>1.000	0.143<br>0.060<br>1.000
S1PR3	0.105<br>0.169<br>1.000	0.035<br>0.646<br>1.000	0.104<br>0.171<br>1.000	0.252<br>< 0.001<br>0.599	0.213<br>0.005<br>1.000	0.140<br>0.066<br>1.000	0.061<br>0.425<br>1.000	0.028<br>0.714<br>1.000	0.209<br>0.006<br>1.000	0.221<br>0.004<br>1.000	0.121<br>0.112<br>1.000	**0.334**<br>< 0.001<br>**0.006**	0.137<br>0.073<br>1.000	0.015<br>0.848<br>1.000	0.148<br>0.053<br>1.000
S1PR4	**0.352**<br>< 0.001<br>**0.002**	**0.583**<br>< 0.001<br>**< 0.001**	**0.501**<br>< 0.001<br>**< 0.001**	0.053<br>0.489<br>1.000	-0.224<br>0.003<br>1.000	-0.079<br>0.300<br>1.000	0.135<br>0.077<br>1.000	-0.104<br>0.174<br>1.000	-0.073<br>0.340<br>1.000	-0.164<br>0.031<br>1.000	**0.500**<br>< 0.001<br>**< 0.001**	0.235<br>0.002<br>1.000	-0.055<br>0.475<br>1.000	-0.255<br>< 0.001<br>0.527	0.115<br>0.130<br>1.000
S1PR5	0.238<br>0.002<br>1.000	0.087<br>0.253<br>1.000	0.162<br>0.033<br>1.000	0.137<br>0.072<br>1.000	0.098<br>0.198<br>1.000	0.056<br>0.461<br>1.000	0.090<br>0.237<br>1.000	0.167<br>0.028<br>1.000	0.208<br>0.006<br>1.000	0.136<br>0.074<br>1.000	0.184<br>0.015<br>1.000	**0.345**<br>< 0.001<br>**0.003**	0.095<br>0.215<br>1.000	-0.066<br>0.387<br>1.000	**0.413**<br>< 0.001<br>**< 0.001**
SGMS1	**0.330**<br>< 0.001<br>**0.008**	0.043<br>0.570<br>1.000	-0.007<br>0.931<br>1.000	0.191<br>0.012<br>1.000	-0.277<br>< 0.001<br>0.178	0.260<br>< 0.001<br>0.408	0.169<br>0.026<br>1.000	0.001<br>0.990<br>1.000	0.141<br>0.065<br>1.000	0.119<br>0.120<br>1.000	0.083<br>0.278<br>1.000	-0.056<br>0.466<br>1.000	0.162<br>0.034<br>1.000	0.137<br>0.071<br>1.000	-0.054<br>0.477<br>1.000
SGMS2	0.288<br>< 0.001<br>0.096	0.190<br>0.012<br>1.000	-0.029<br>0.709<br>1.000	0.035<br>0.644<br>1.000	-0.195<br>0.010<br>1.000	0.065<br>0.395<br>1.000	-0.043<br>0.578<br>1.000	-0.173<br>0.023<br>1.000	0.042<br>0.583<br>1.000	-0.037<br>0.633<br>1.000	-0.021<br>0.782<br>1.000	0.268<br>< 0.001<br>0.277	-0.168<br>0.027<br>1.000	0.024<br>0.755<br>1.000	0.176<br>0.020<br>1.000
SGPL1	0.176<br>0.021<br>1.000	-0.024<br>0.752<br>1.000	-0.023<br>0.759<br>1.000	**0.401**<br>< 0.001<br>**< 0.001**	0.017<br>0.823<br>1.000	**0.522**<br>< 0.001<br>**< 0.001**	0.110<br>0.151<br>1.000	0.207<br>0.006<br>1.000	**0.390**<br>< 0.001<br>**< 0.001**	0.261<br>< 0.001<br>0.390	-0.069<br>0.366<br>1.000	-0.108<br>0.157<br>1.000	**0.351**<br>< 0.001<br>**0.002**	**0.304**<br>< 0.001<br>**0.039**	0.040<br>0.598<br>1.000
SGPP1	**0.441**<br>< 0.001<br>**< 0.001**	0.176<br>0.020<br>1.000	0.246<br>0.001<br>0.789	0.160<br>0.036<br>1.000	**-0.307**<br>< 0.001<br>**0.032**	0.048<br>0.533<br>1.000	0.204<br>0.007<br>1.000	-0.045<br>0.560<br>1.000	0.027<br>0.727<br>1.000	-0.016<br>0.830<br>1.000	0.284<br>< 0.001<br>0.119	**0.316**<br>< 0.001<br>**0.019**	-0.128<br>0.093<br>1.000	**-0.340**<br>< 0.001<br>**0.004**	0.168<br>0.027<br>1.000
SGPP2	0.062<br>0.420<br>1.000	0.139<br>0.068<br>1.000	-0.072<br>0.345<br>1.000	-0.145<br>0.058<br>1.000	-0.096<br>0.210<br>1.000	0.105<br>0.167<br>1.000	0.077<br>0.314<br>1.000	-0.001<br>0.986<br>1.000	-0.026<br>0.730<br>1.000	-0.001<br>0.992<br>1.000	-0.122<br>0.108<br>1.000	-0.169<br>0.027<br>1.000	0.148<br>0.052<br>1.000	0.071<br>0.356<br>1.000	-0.297<br>< 0.001<br>0.058
SMPD1	**0.362**<br>< 0.001<br>**< 0.001**	0.241<br>0.001<br>1.000	0.194<br>0.011<br>1.000	**0.304**<br>< 0.001<br>**0.039**	0.001<br>0.986<br>1.000	0.125<br>0.100<br>1.000	0.071<br>0.354<br>1.000	0.121<br>0.111<br>1.000	**0.304**<br>< 0.001<br>**0.039**	0.075<br>0.329<br>1.000	**0.302**<br>< 0.001<br>**0.045**	**0.518**<br>< 0.001<br>**< 0.001**	-0.039<br>0.608<br>1.000	-0.161<br>0.035<br>1.000	**0.483**<br>< 0.001<br>**< 0.001**
SMPD2	0.162<br>0.034<br>1.000	0.041<br>0.593<br>1.000	0.143<br>0.060<br>1.000	0.221<br>0.003<br>1.000	-0.111<br>0.144<br>1.000	0.233<br>0.002<br>1.000	0.204<br>0.007<br>1.000	-0.021<br>0.783<br>1.000	0.237<br>0.002<br>1.000	0.119<br>0.119<br>1.000	0.059<br>0.441<br>1.000	-0.086<br>0.263<br>1.000	0.280<br>< 0.001<br>0.151	0.188<br>0.013<br>1.000	-0.119<br>0.119<br>1.000
SMPD3	0.237<br>0.002<br>1.000	0.054<br>0.477<br>1.000	0.224<br>0.003<br>1.000	0.078<br>0.308<br>1.000	-0.152<br>0.045<br>1.000	0.066<br>0.387<br>1.000	0.089<br>0.246<br>1.000	0.061<br>0.427<br>1.000	0.027<br>0.720<br>1.000	0.034<br>0.661<br>1.000	0.253<br>< 0.001<br>0.558	-0.070<br>0.358<br>1.000	0.044<br>0.565<br>1.000	-0.146<br>0.055<br>1.000	-0.031<br>0.684<br>1.000
SPHK1	0.151<br>0.047<br>1.000	**0.333**<br>< 0.001<br>**0.007**	0.131<br>0.086<br>1.000	-0.019<br>0.805<br>1.000	0.030<br>0.695<br>1.000	-0.223<br>0.003<br>1.000	-0.088<br>0.250<br>1.000	-0.100<br>0.191<br>1.000	-0.065<br>0.393<br>1.000	-0.044<br>0.568<br>1.000	0.072<br>0.348<br>1.000	**0.449**<br>< 0.001<br>**< 0.001**	-0.213<br>0.005<br>1.000	-0.260<br>< 0.001<br>0.408	**0.401**<br>< 0.001<br>**< 0.001**
SPHK2	0.066<br>0.386<br>1.000	-0.040<br>0.605<br>1.000	0.150<br>0.048<br>1.000	**0.420**<br>< 0.001<br>**< 0.001**	**0.423**<br>< 0.001<br>**< 0.001**	0.283<br>< 0.001<br>0.130	0.063<br>0.413<br>1.000	0.265<br>< 0.001<br>0.323	0.266<br>< 0.001<br>0.312	0.054<br>0.478<br>1.000	0.239<br>0.002<br>1.000	0.081<br>0.292<br>1.000	**0.309**<br>< 0.001<br>**0.030**	0.215<br>0.004<br>1.000	0.278<br>< 0.001<br>0.162
UGCG	**0.365**<br>< 0.001<br>**< 0.001**	0.271<br>< 0.001<br>0.239	0.123<br>0.106<br>1.000	0.189<br>0.013<br>1.000	-0.207<br>0.006<br>1.000	0.185<br>0.015<br>1.000	0.014<br>0.851<br>1.000	-0.009<br>0.906<br>1.000	0.269<br>< 0.001<br>0.263	0.210<br>0.006<br>1.000	0.085<br>0.264<br>1.000	**0.326**<br>< 0.001<br>**0.010**	0.054<br>0.480<br>1.000	-0.203<br>0.007<br>1.000	0.101<br>0.185<br>1.000
UGT8	-0.175<br>0.021<br>1.000	-0.022<br>0.769<br>1.000	-0.039<br>0.609<br>1.000	0.075<br>0.327<br>1.000	0.072<br>0.345<br>1.000	0.154<br>0.043<br>1.000	0.076<br>0.317<br>1.000	0.015<br>0.848<br>1.000	0.154<br>0.043<br>1.000	0.212<br>0.005<br>1.000	-0.122<br>0.111<br>1.000	-0.058<br>0.445<br>1.000	**0.312**<br>< 0.001<br>**0.024**	**0.396**<br>< 0.001<br>**< 0.001**	-0.154<br>0.043<br>1.000
CD14	**0.363**<br>< 0.001<br>**< 0.001**	**0.490**<br>< 0.001<br>**< 0.001**	**0.443**<br>< 0.001<br>**< 0.001**	0.061<br>0.425<br>1.000	-0.220<br>0.004<br>1.000	-0.017<br>0.821<br>1.000	0.052<br>0.494<br>1.000	-0.135<br>0.076<br>1.000	-0.113<br>0.139<br>1.000	-0.164<br>0.031<br>1.000	**0.360**<br>< 0.001<br>**0.001**	0.270<br>< 0.001<br>0.245	-0.111<br>0.148<br>1.000	-0.228<br>0.003<br>1.000	0.231<br>0.002<br>1.000
CD68	**0.373**<br>< 0.001<br>**< 0.001**	**0.416**<br>< 0.001<br>**< 0.001**	0.230<br>0.002<br>1.000	0.157<br>0.039<br>1.000	-0.234<br>0.002<br>1.000	0.122<br>0.111<br>1.000	0.103<br>0.177<br>1.000	-0.139<br>0.068<br>1.000	-0.106<br>0.166<br>1.000	-0.055<br>0.470<br>1.000	0.189<br>0.013<br>1.000	0.267<br>< 0.001<br>0.288	0.041<br>0.596<br>1.000	-0.204<br>0.007<br>1.000	0.263<br>< 0.001<br>0.353
CD163	**0.391**<br>< 0.001<br>**< 0.001**	**0.360**<br>< 0.001<br>**0.001**	**0.456**<br>< 0.001<br>**< 0.001**	0.083<br>0.279<br>1.000	-0.228<br>0.003<br>1.000	0.026<br>0.739<br>1.000	0.035<br>0.644<br>1.000	-0.078<br>0.308<br>1.000	-0.023<br>0.765<br>1.000	-0.054<br>0.477<br>1.000	**0.344**<br>< 0.001<br>**0.003**	0.239<br>0.002<br>1.000	-0.085<br>0.267<br>1.000	-0.247<br>0.001<br>0.746	0.237<br>0.002<br>1.000
CD3E	0.258<br>< 0.001<br>0.447	**0.601**<br>< 0.001<br>**< 0.001**	**0.439**<br>< 0.001<br>**< 0.001**	-0.044<br>0.564<br>1.000	-0.217<br>0.004<br>1.000	-0.129<br>0.091<br>1.000	0.179<br>0.018<br>1.000	-0.066<br>0.388<br>1.000	-0.158<br>0.038<br>1.000	-0.217<br>0.004<br>1.000	**0.449**<br>< 0.001<br>**< 0.001**	0.281<br>< 0.001<br>0.138	-0.152<br>0.045<br>1.000	-0.185<br>0.015<br>1.000	0.186<br>0.014<br>1.000
IGHG1	0.066<br>0.385<br>1.000	**0.414**<br>< 0.001<br>**< 0.001**	**0.359**<br>< 0.001<br>**0.001**	-0.098<br>0.202<br>1.000	-0.102<br>0.180<br>1.000	-0.130<br>0.089<br>1.000	0.083<br>0.279<br>1.000	-0.151<br>0.048<br>1.000	-0.150<br>0.048<br>1.000	-0.123<br>0.106<br>1.000	**0.307**<br>< 0.001<br>**0.033**	0.151<br>0.048<br>1.000	-0.087<br>0.253<br>1.000	-0.102<br>0.180<br>1.000	0.025<br>0.741<br>1.000
IGHM	0.069<br>0.365<br>1.000	**0.456**<br>< 0.001<br>**< 0.001**	**0.359**<br>< 0.001<br>**0.001**	-0.139<br>0.068<br>1.000	-0.113<br>0.137<br>1.000	-0.110<br>0.150<br>1.000	0.128<br>0.093<br>1.000	-0.103<br>0.177<br>1.000	-0.180<br>0.018<br>1.000	-0.132<br>0.084<br>1.000	**0.353**<br>< 0.001<br>**0.002**	0.129<br>0.090<br>1.000	-0.081<br>0.289<br>1.000	-0.010<br>0.896<br>1.000	0.015<br>0.844<br>1.000
MS4A1	0.222<br>0.003<br>1.000	**0.466**<br>< 0.001<br>**< 0.001**	**0.382**<br>< 0.001<br>**< 0.001**	0.033<br>0.667<br>1.000	-0.077<br>0.312<br>1.000	-0.090<br>0.241<br>1.000	0.173<br>0.023<br>1.000	-0.084<br>0.274<br>1.000	-0.096<br>0.210<br>1.000	-0.095<br>0.215<br>1.000	**0.395**<br>< 0.001<br>**< 0.001**	0.240<br>0.001<br>1.000	-0.046<br>0.545<br>1.000	-0.167<br>0.028<br>1.000	0.122<br>0.109<br>1.000
PTPRC	**0.383**<br>< 0.001<br>**< 0.001**	**0.608**<br>< 0.001<br>**< 0.001**	**0.486**<br>< 0.001<br>**< 0.001**	0.083<br>0.279<br>1.000	-0.285<br>< 0.001<br>0.113	-0.036<br>0.636<br>1.000	0.177<br>0.020<br>1.000	-0.133<br>0.081<br>1.000	-0.034<br>0.654<br>1.000	-0.053<br>0.489<br>1.000	**0.335**<br>< 0.001<br>**0.006**	0.238<br>0.002<br>1.000	-0.032<br>0.677<br>1.000	-0.241<br>0.001<br>1.000	0.162<br>0.034<br>1.000

	LPAR5	LPAR6	NAAA	PPAP2A	PPAP2B	PPAP2C	S1PR1	S1PR2	S1PR3	S1PR4	S1PR5	SGMS1	SGMS2
LPAR6	**0.613**<br>< 0.001<br>**< 0.001**												
NAAA	**0.448**<br>< 0.001<br>**< 0.001**	**0.321**<br>< 0.001<br>**0.014**											
PPAP2A	0.275<br>< 0.001<br>0.190	**0.375**<br>< 0.001<br>**< 0.001**	**0.382**<br>< 0.001<br>**< 0.001**										
PPAP2B	0.068<br>0.374<br>1.000	0.110<br>0.151<br>1.000	0.032<br>0.677<br>1.000	**0.397**<br>< 0.001<br>**< 0.001**									
PPAP2C	0.084<br>0.269<br>1.000	0.115<br>0.132<br>1.000	-0.043<br>0.571<br>1.000	-0.139<br>0.068<br>1.000	-0.074<br>0.335<br>1.000								
S1PR1	0.291<br>< 0.001<br>0.084	**0.433**<br>< 0.001<br>**< 0.001**	**0.322**<br>< 0.001<br>**0.013**	**0.716**<br>< 0.001<br>**< 0.001**	**0.316**<br>< 0.001<br>**0.018**	-0.192<br>0.011<br>1.000							
S1PR2	-0.005<br>0.944<br>1.000	0.091<br>0.235<br>1.000	0.017<br>0.820<br>1.000	0.181<br>0.017<br>1.000	0.184<br>0.015<br>1.000	0.012<br>0.877<br>1.000	0.184<br>0.016<br>1.000						
S1PR3	0.003<br>0.966<br>1.000	0.081<br>0.291<br>1.000	0.019<br>0.809<br>1.000	**0.321**<br>< 0.001<br>**0.014**	**0.312**<br>< 0.001<br>**0.025**	-0.140<br>0.066<br>1.000	**0.342**<br>< 0.001<br>**0.004**	0.267<br>< 0.001<br>0.289					
S1PR4	**0.496**<br>< 0.001<br>**< 0.001**	**0.367**<br>< 0.001<br>**< 0.001**	**0.441**<br>< 0.001<br>**< 0.001**	**0.360**<br>< 0.001<br>**0.001**	-0.012<br>0.874<br>1.000	-0.035<br>0.648<br>1.000	**0.431**<br>< 0.001<br>**< 0.001**	0.004<br>0.963<br>1.000	0.030<br>0.698<br>1.000				
S1PR5	**0.354**<br>< 0.001<br>**0.002**	0.285<br>< 0.001<br>0.113	0.223<br>0.003<br>1.000	**0.450**<br>< 0.001<br>**< 0.001**	0.221<br>0.003<br>1.000	-0.209<br>0.006<br>1.000	**0.474**<br>< 0.001<br>**< 0.001**	0.262<br>< 0.001<br>0.367	**0.319**<br>< 0.001<br>**0.016**	0.236<br>0.002<br>1.000			
SGMS1	0.044<br>0.568<br>1.000	**0.307**<br>< 0.001<br>**0.034**	0.118<br>0.123<br>1.000	-0.131<br>0.086<br>1.000	-0.135<br>0.076<br>1.000	0.106<br>0.164<br>1.000	-0.033<br>0.662<br>1.000	-0.031<br>0.688<br>1.000	-0.125<br>0.101<br>1.000	0.063<br>0.414<br>1.000	-0.093<br>0.225<br>1.000		
SGMS2	0.206<br>0.007<br>1.000	0.276<br>< 0.001<br>0.187	0.256<br>< 0.001<br>0.489	0.215<br>0.004<br>1.000	-0.161<br>0.035<br>1.000	**0.311**<br>< 0.001<br>**0.025**	0.014<br>0.860<br>1.000	-0.077<br>0.315<br>1.000	-0.078<br>0.307<br>1.000	0.110<br>0.148<br>1.000	0.048<br>0.534<br>1.000	0.228<br>0.003<br>1.000	
SGPL1	0.046<br>0.547<br>1.000	0.197<br>0.010<br>1.000	-0.005<br>0.949<br>1.000	-0.140<br>0.066<br>1.000	0.011<br>0.884<br>1.000	0.218<br>0.004<br>1.000	-0.156<br>0.041<br>1.000	0.190<br>0.012<br>1.000	0.161<br>0.034<br>1.000	-0.021<br>0.784<br>1.000	-0.019<br>0.803<br>1.000	**0.317**<br>< 0.001<br>**0.018**	0.085<br>0.268<br>1.000
SGPP1	**0.340**<br>< 0.001<br>**0.004**	**0.364**<br>< 0.001<br>**< 0.001**	**0.365**<br>< 0.001<br>**< 0.001**	**0.398**<br>< 0.001<br>**< 0.001**	0.089<br>0.245<br>1.000	0.010<br>0.893<br>1.000	0.341<br>0.000<br>0.004	0.033<br>0.663<br>1.000	0.124<br>0.105<br>1.000	**0.304**<br>< 0.001<br>**0.038**	0.132<br>0.084<br>1.000	0.256<br>< 0.001<br>0.497	0.149<br>0.051<br>1.000
SGPP2	-0.001<br>0.985<br>1.000	-0.164<br>0.031<br>1.000	-0.038<br>0.619<br>1.000	-0.262<br>< 0.001<br>0.363	-0.064<br>0.401<br>1.000	0.203<br>0.007<br>1.000	-0.288<br>< 0.001<br>0.098	-0.064<br>0.401<br>1.000	0.019<br>0.809<br>1.000	-0.011<br>0.883<br>1.000	-0.152<br>0.045<br>1.000	0.246<br>0.001<br>0.803	0.122<br>0.109<br>1.000
SMPD1	**0.309**<br>< 0.001<br>**0.029**	**0.324**<br>< 0.001<br>**0.011**	**0.476**<br>< 0.001<br>**< 0.001**	**0.629**<br>< 0.001<br>**< 0.001**	0.236<br>0.002<br>1.000	-0.060<br>0.436<br>1.000	**0.528**<br>< 0.001<br>**< 0.001**	0.289<br>< 0.001<br>0.094	0.226<br>0.003<br>1.000	**0.326**<br>< 0.001<br>**0.010**	**0.413**<br>< 0.001<br>**< 0.001**	-0.079<br>0.302<br>1.000	**0.335**<br>< 0.001<br>**0.006**
SMPD2	0.117<br>0.125<br>1.000	0.196<br>0.010<br>1.000	0.065<br>0.399<br>1.000	-0.104<br>0.172<br>1.000	-0.040<br>0.598<br>1.000	0.107<br>0.163<br>1.000	0.040<br>0.601<br>1.000	0.130<br>0.088<br>1.000	0.105<br>0.171<br>1.000	0.016<br>0.832<br>1.000	-0.018<br>0.815<br>1.000	**0.476**<br>< 0.001<br>**< 0.001**	0.112<br>0.144<br>1.000
SMPD3	0.115<br>0.132<br>1.000	0.149<br>0.050<br>1.000	0.023<br>0.762<br>1.000	0.013<br>0.862<br>1.000	-0.029<br>0.703<br>1.000	0.149<br>0.051<br>1.000	0.057<br>0.454<br>1.000	0.044<br>0.563<br>1.000	-0.054<br>0.482<br>1.000	0.190<br>0.012<br>1.000	-0.065<br>0.398<br>1.000	0.237<br>0.002<br>1.000	-0.018<br>0.811<br>1.000
SPHK1	0.267<br>< 0.001<br>0.292	0.153<br>0.044<br>1.000	**0.316**<br>< 0.001<br>**0.019**	**0.361**<br>< 0.001<br>**< 0.001**	0.071<br>0.356<br>1.000	-0.130<br>0.088<br>1.000	0.266<br>< 0.001<br>0.303	0.226<br>0.003<br>1.000	0.171<br>0.024<br>1.000	**0.386**<br>< 0.001<br>**< 0.001**	**0.402**<br>< 0.001<br>**< 0.001**	-0.262<br>< 0.001<br>0.381	0.236<br>0.002<br>1.000
SPHK2	0.157<br>0.039<br>1.000	0.284<br>< 0.001<br>0.123	-0.034<br>0.655<br>1.000	0.160<br>0.036<br>1.000	**0.355**<br>< 0.001<br>**0.001**	-0.045<br>0.558<br>1.000	0.244<br>0.001<br>0.882	0.102<br>0.184<br>1.000	0.266<br>< 0.001<br>0.309	0.036<br>0.635<br>1.000	0.225<br>0.003<br>1.000	0.001<br>0.992<br>1.000	-0.107<br>0.163<br>1.000
UGCG	0.182<br>0.017<br>1.000	0.204<br>0.007<br>1.000	0.291<br>< 0.001<br>0.082	0.260<br>< 0.001<br>0.414	0.103<br>0.179<br>1.000	0.078<br>0.307<br>1.000	0.181<br>0.017<br>1.000	0.223<br>0.003<br>1.000	0.240<br>0.001<br>1.000	0.297<br>< 0.001<br>0.059	0.261<br>< 0.001<br>0.397	0.186<br>0.014<br>1.000	0.219<br>0.004<br>1.000
UGT8	-0.021<br>0.782<br>1.000	-0.037<br>0.633<br>1.000	-0.004<br>0.957<br>1.000	-0.246<br>0.001<br>0.799	-0.085<br>0.268<br>1.000	0.052<br>0.493<br>1.000	-0.159<br>0.036<br>1.000	-0.026<br>0.734<br>1.000	0.132<br>0.084<br>1.000	-0.085<br>0.269<br>1.000	-0.040<br>0.602<br>1.000	0.165<br>0.030<br>1.000	0.034<br>0.655<br>1.000
CD14	**0.653**<br>< 0.001<br>**< 0.001**	**0.466**<br>< 0.001<br>**< 0.001**	**0.419**<br>< 0.001<br>**< 0.001**	0.303<br>< 0.001<br>0.041	0.071<br>0.353<br>1.000	0.121<br>0.113<br>1.000	0.290<br>< 0.001<br>0.088	0.022<br>0.771<br>1.000	0.063<br>0.409<br>1.000	**0.578**<br>< 0.001<br>**< 0.001**	0.150<br>0.049<br>1.000	0.102<br>0.183<br>1.000	0.286<br>< 0.001<br>0.109
CD68	**0.383**<br>< 0.001<br>**< 0.001**	0.264<br>< 0.001<br>0.333	0.256<br>< 0.001<br>0.487	0.182<br>0.017<br>1.000	0.077<br>0.316<br>1.000	0.075<br>0.328<br>1.000	0.143<br>0.061<br>1.000	0.079<br>0.299<br>1.000	0.057<br>0.457<br>1.000	**0.443**<br>< 0.001<br>**< 0.001**	0.092<br>0.227<br>1.000	0.144<br>0.059<br>1.000	0.125<br>0.101<br>1.000
CD163	**0.463**<br>< 0.001<br>**< 0.001**	**0.413**<br>< 0.001<br>**< 0.001**	**0.377**<br>< 0.001<br>**< 0.001**	**0.320**<br>< 0.001<br>**0.015**	0.028<br>0.711<br>1.000	-0.006<br>0.943<br>1.000	**0.304**<br>< 0.001<br>**0.038**	0.115<br>0.132<br>1.000	0.117<br>0.124<br>1.000	**0.575**<br>< 0.001<br>**< 0.001**	0.146<br>0.055<br>1.000	0.158<br>0.038<br>1.000	0.137<br>0.073<br>1.000
CD3E	**0.560**<br>< 0.001<br>**< 0.001**	**0.371**<br>< 0.001<br>**< 0.001**	**0.517**<br>< 0.001<br>**< 0.001**	**0.395**<br>< 0.001<br>**< 0.001**	0.020<br>0.797<br>1.000	-0.058<br>0.446<br>1.000	**0.435**<br>< 0.001<br>**< 0.001**	-0.044<br>0.566<br>1.000	0.040<br>0.597<br>1.000	**0.827**<br>< 0.001<br>**< 0.001**	0.233<br>0.002<br>1.000	0.030<br>0.698<br>1.000	0.198<br>0.009<br>1.000
IGHG1	**0.311**<br>< 0.001<br>**0.025**	0.175<br>0.022<br>1.000	0.245<br>0.001<br>0.838	0.237<br>0.002<br>1.000	0.045<br>0.559<br>1.000	-0.114<br>0.137<br>1.000	0.184<br>0.015<br>1.000	-0.067<br>0.385<br>1.000	0.069<br>0.365<br>1.000	**0.652**<br>< 0.001<br>**< 0.001**	0.125<br>0.102<br>1.000	-0.105<br>0.168<br>1.000	0.024<br>0.750<br>1.000
IGHM	**0.382**<br>< 0.001<br>**< 0.001**	0.199<br>0.009<br>1.000	0.260<br>< 0.001<br>0.406	0.235<br>0.002<br>1.000	0.068<br>0.372<br>1.000	-0.048<br>0.530<br>1.000	0.260<br>< 0.001<br>0.401	-0.102<br>0.182<br>1.000	0.040<br>0.599<br>1.000	**0.704**<br>< 0.001<br>**< 0.001**	0.173<br>0.023<br>1.000	-0.056<br>0.460<br>1.000	0.078<br>0.309<br>1.000
MS4A1	**0.398**<br>< 0.001<br>**< 0.001**	**0.304**<br>< 0.001<br>**0.040**	**0.377**<br>< 0.001<br>**< 0.001**	**0.358**<br>< 0.001<br>0.001	0.005<br>0.951<br>1.000	-0.147<br>0.054<br>1.000	**0.454**<br>< 0.001<br>**< 0.001**	0.007<br>0.930<br>1.000	0.083<br>0.276<br>1.000	**0.785**<br>< 0.001<br>**< 0.001**	0.270<br>< 0.001<br>0.254	0.011<br>0.882<br>1.000	0.052<br>0.498<br>1.000
PTPRC	**0.655**<br>< 0.001<br>**< 0.001**	**0.462**<br>< 0.001<br>**< 0.001**	**0.515**<br>< 0.001<br>**< 0.001**	**0.309**<br>< 0.001<br>**0.029**	-0.036<br>0.636<br>1.000	-0.021<br>0.784<br>1.000	**0.329**<br>< 0.001<br>0.009	0.030<br>0.692<br>1.000	0.094<br>0.221<br>1.000	**0.811**<br>< 0.001<br>**< 0.001**	0.233<br>0.002<br>1.000	0.163<br>0.032<br>1.000	0.187<br>0.014<br>1.000

	SGPL1	SGPP1	SGPP2	SMPD1	SMPD2	SMPD3	SPHK1	SPHK2	UGCG	UGT8	CD14	CD68	CD163	CD3E	IGHG1	IGHM	MS4A1
SGPP1	-0.016<br>0.834<br>1.000																
SGPP2	0.218<br>0.004<br>1.000	0.038<br>0.620<br>1.000															
SMPD1	-0.003<br>0.968<br>1.000	**0.337**<br>< 0.001<br>**0.005**	-0.138<br>0.071<br>1.000														
SMPD2	0.263<br>< 0.001<br>0.355	0.166<br>0.029<br>1.000	0.279<br>< 0.001<br>0.158	0.045<br>0.561<br>1.000													
SMPD3	0.003<br>0.973<br>1.000	0.283<br>< 0.001<br>0.126	0.107<br>0.163<br>1.000	0.001<br>0.986<br>1.000	0.248<br>< 0.001<br>0.715												
SPHK1	-0.160<br>0.036<br>1.000	0.113<br>0.138<br>1.000	-0.191<br>0.012<br>1.000	**0.373**<br>< 0.001<br>**< 0.001**	-0.283<br>< 0.001<br>0.127	-0.243<br>0.001<br>0.928											
SPHK2	**0.303**<br>< 0.001<br>**0.040**	-0.103<br>0.177<br>1.000	0.030<br>0.692<br>1.000	0.108<br>0.156<br>1.000	0.072<br>0.349<br>1.000	-0.075<br>0.329<br>1.000	-0.041<br>0.595<br>1.000										
UGCG	0.133<br>0.081<br>1.000	**0.364**<br>< 0.001<br>**< 0.001**	0.099<br>0.194<br>1.000	0.315<br>< 0.001<br>0.021	0.292<br>< 0.001<br>0.078	0.278<br>< 0.001<br>0.165	0.254<br>< 0.001<br>0.553	-0.154<br>0.043<br>1.000									
UGT8	0.233<br>0.002<br>1.000	-0.087<br>0.254<br>1.000	0.222<br>0.003<br>1.000	-0.085<br>0.267<br>1.000	0.219<br>0.004<br>1.000	0.041<br>0.588<br>1.000	-0.234<br>0.002<br>1.000	0.159<br>0.036<br>1.000	0.023<br>0.766<br>1.000								
CD14	0.037<br>0.630<br>1.000	**0.396**<br>< 0.001<br>**< 0.001**	0.129<br>0.092<br>1.000	**0.325**<br>< 0.001<br>**0.011**	0.042<br>0.580<br>1.000	0.136<br>0.075<br>1.000	**0.469**<br>< 0.001<br>**< 0.001**	0.017<br>0.826<br>1.000	0.264<br>< 0.001<br>0.332	-0.202<br>0.008<br>1.000							
CD68	0.152<br>0.046<br>1.000	0.244<br>0.001<br>0.887	0.067<br>0.381<br>1.000	0.250<br>< 0.001<br>0.647	-0.016<br>0.837<br>1.000	0.093<br>0.221<br>1.000	**0.385**<br>< 0.001<br>**< 0.001**	0.017<br>0.825<br>1.000	**0.312**<br>< 0.001<br>**0.024**	-0.036<br>0.639<br>1.000	**0.548**<br>< 0.001<br>**< 0.001**						
CD163	0.110<br>0.149<br>1.000	**0.433**<br>< 0.001<br>**< 0.001**	0.061<br>0.428<br>1.000	**0.332**<br>< 0.001<br>**0.007**	0.083<br>0.279<br>1.000	0.141<br>0.065<br>1.000	**0.469**<br>< 0.001<br>**< 0.001**	0.045<br>0.557<br>1.000	**0.306**<br>< 0.001<br>**0.034**	-0.175<br>0.021<br>1.000	**0.800**<br>< 0.001<br>**< 0.001**	**0.581**<br>< 0.001<br>**< 0.001**					
CD3E	-0.071<br>0.354<br>1.000	0.272<br>< 0.001<br>0.225	0.035<br>0.649<br>1.000	**0.331**<br>< 0.001<br>**0.007**	0.040<br>0.604<br>1.000	0.043<br>0.578<br>1.000	**0.420**<br>< 0.001<br>**< 0.001**	0.047<br>0.537<br>1.000	0.190<br>0.012<br>1.000	-0.071<br>0.356<br>1.000	**0.582**<br>< 0.001<br>**< 0.001**	**0.393**<br>< 0.001<br>**< 0.001**	**0.533**<br>< 0.001<br>**< 0.001**				
IGHG1	-0.025<br>0.748<br>1.000	0.034<br>0.658<br>1.000	0.026<br>0.735<br>1.000	0.223<br>0.003<br>1.000	-0.094<br>0.221<br>1.000	-0.047<br>0.539<br>1.000	**0.324**<br>< 0.001<br>**0.011**	0.065<br>0.395<br>1.000	0.083<br>0.279<br>1.000	-0.122<br>0.111<br>1.000	**0.390**<br>< 0.001<br>**< 0.001**	0.240<br>0.001<br>1.000	**0.395**<br>< 0.001<br>**< 0.001**	**0.723**<br>< 0.001<br>**< 0.001**			
IGHM	-0.016<br>0.832<br>1.000	0.060<br>0.435<br>1.000	0.080<br>0.294<br>1.000	0.226<br>0.003<br>1.000	-0.031<br>0.687<br>1.000	0.006<br>0.937<br>1.000	0.296<br>< 0.001<br>0.064	0.126<br>0.099<br>1.000	0.077<br>0.317<br>1.000	-0.016<br>0.836<br>1.000	**0.402**<br>< 0.001<br>**< 0.001**	0.288<br>< 0.001<br>0.096	**0.386**<br>< 0.001<br>**< 0.001**	**0.767**<br>< 0.001<br>**< 0.001**	**0.839**<br>< 0.001<br>**< 0.001**		
MS4A1	-0.068<br>0.373<br>1.000	0.156<br>0.041<br>1.000	-0.042<br>0.587<br>1.000	0.278<br>< 0.001<br>0.165	-0.018<br>0.818<br>1.000	0.011<br>0.885<br>1.000	**0.357**<br>< 0.001<br>**0.001**	0.038<br>0.616<br>1.000	0.173<br>0.023<br>1.000	-0.020<br>0.798<br>1.000	**0.382**<br>< 0.001<br>**< 0.001**	0.287<br>< 0.001<br>0.100	**0.353**<br>< 0.001<br>0.002	**0.788**<br>< 0.001<br>**< 0.001**	**0.718**<br>< 0.001<br>**< 0.001**	**0.700**<br>< 0.001<br>**< 0.001**	
PTPRC	0.046<br>0.544<br>1.000	**0.448**<br>< 0.001<br>**< 0.001**	0.066<br>0.385<br>1.000	**0.339**<br>< 0.001<br>**0.004**	0.136<br>0.075<br>1.000	0.184<br>0.015<br>1.000	**0.426**<br>< 0.001<br>**< 0.001**	-0.039<br>0.609<br>1.000	**0.382**<br>< 0.001<br>**< 0.001**	-0.015<br>0.841<br>1.000	**0.710**<br>< 0.001<br>**< 0.001**	**0.507**<br>< 0.001<br>**< 0.001**	**0.683**<br>< 0.001<br>**< 0.001**	**0.821**<br>< 0.001<br>**< 0.001**	**0.623**<br>< 0.001<br>**< 0.001**	**0.619**<br>< 0.001<br>**< 0.001**	**0.692**<br>< 0.001<br>**< 0.001**

Table S3. Clinicopathological characteristics of independent patient cohort (n = 19) used for gene expression profiling

**Patients (%)**	**19 (100.0)**
Progression-free survival	
Number of recurrencies (%)	11 (57.9)
Overall survival	
Number of deaths (%)	8 (42.1)
Histology (%)	
Serous	12 (63.2)
Endometrioid	5 (26.3)
Mucinous	1 (5.3)
Clear cell carcinoma	1 (5.3)
FIGO (%)	
I	6 (31.6)
II	2 (10.6)
III	10 (52.6)
IV	1 (5.3)
Histological Grading (%)	
1	4 (21.1)
2	6 (31.6)
3	9 (47.4)
Response to first-line chemotherapy (%, 5 missing [26.3 %])	
Responder	6 (31.6)
Non-Responder	8 (42.1)

Table S4. Univariate Cox regression analysis of clinicopathological variables and gene profiling-derived data sets for OS and PFS.

	OS			PFS		
	HR	95% CI	p	HR	95% CI	p
**Age**	**1.037**	**1.012 - 1.064**	**0.004**	1.008	0.991 - 1.026	0.352
**Histology**	1.396	0.653 - 2.984	0.389	0.918	0.489 - 1.725	0.79
**FIGO stage**	1.838	0.989 - 3.415	0.054	**2.464**	**1.579 - 3.844**	**< 0.001**
**Grading**	**2.798**	**1.258 - 6.227**	**0.012**	**1.743**	**1.082 - 2.806**	**0.022**
**Peritoneal carcinomatosis**	**3.427**	**1.526 - 7.643**	**0.003**	**3.196**	**1.911 - 5.345**	**< 0.001**
**Residual disease**	**2.250**	**1.268 - 3.990**	**0.006**	**1.970**	**1.266 - 3.066**	**0.003**
ASAH1	1.081	0.683 - 1.710	0.741	1.037	0.737 - 1.458	0.835
CD1B	0.963	0.878 - 1.056	0.418	**0.936**	**0.880 - 0.996**	**0.037**
CD1D	1.050	0.849 - 1.298	0.653	0.922	0.804 - 1.059	0.25
CERK	0.988	0.694 - 1.407	0.948	1.178	0.932 - 1.489	0.17
CERS1	1.105	0.939 - 1.299	0.228	0.949	0.860 - 1.047	0.294
CERS2	0.661	0.399 - 1.094	0.107	0.718	0.505 - 1.022	0.066
CERS3	1.019	0.914 - 1.135	0.736	1.040	0.966 - 1.120	0.296
CERS4	0.954	0.732 - 1.242	0.725	0.924	0.767 - 1.114	0.409
CERS5	0.927	0.564 - 1.523	0.764	0.847	0.608 - 1.180	0.326
CERS6	0.775	0.518 - 1.160	0.216	0.940	0.714 - 1.238	0.661
ENPP2	0.926	0.714 - 1.200	0.56	0.918	0.768 - 1.099	0.352
LPAR1	1.000	0.772 - 1.295	0.999	1.000	0.836 - 1.195	0.997
LPAR2	0.848	0.579 - 1.242	0.398	0.803	0.612 - 1.055	0.115
LPAR3	**0.883**	**0.804 - 0.969**	**0.009**	1.040	0.961 - 1.125	0.33
LPAR4	1.098	0.909 - 1.326	0.332	1.122	0.982 - 1.281	0.09
LPAR5	1.075	0.817 - 1.416	0.605	0.950	0.785 - 1.148	0.593
LPAR6	1.179	0.873 - 1.591	0.283	1.084	0.877 - 1.339	0.457
NAAA	1.056	0.771 - 1.446	0.735	0.888	0.715 - 1.103	0.282
PPAP2A	1.029	0.760 - 1.393	0.854	0.927	0.736 - 1.167	0.517
PPAP2B	0.745	0.552 - 1.005	0.054	0.891	0.727 - 1.092	0.267
PPAP2C	1.048	0.879 - 1.249	0.602	0.974	0.864 - 1.098	0.668
S1PR1	1.035	0.768 - 1.395	0.82	0.945	0.770 - 1.160	0.589
S1PR2	0.834	0.607 - 1.146	0.263	0.944	0.758 - 1.176	0.608
S1PR3	0.849	0.635 - 1.135	0.27	1.013	0.830 - 1.238	0.897
S1PR4	1.054	0.869 - 1.279	0.591	0.934	0.818 - 1.068	0.319
S1PR5	1.191	0.950 - 1.494	0.13	1.039	0.886 - 1.219	0.634
SGMS1	0.818	0.622 - 1.074	0.147	1.117	0.952 - 1.456	0.132
SGMS2	1.012	0.767 - 1.334	0.935	1.059	0.874 - 1.284	0.555
SGPL1	0.747	0.502 - 1.113	0.151	0.945	0.706 - 1.264	0.702
SGPP1	1.150	0.734 - 1.802	0.541	1.206	0.895 - 1.625	0.219
SGPP2	0.812	0.657 - 1.002	0.053	0.860	0.733 - 1.010	0.065
SMPD1	0.690	0.440 - 1.081	0.105	0.882	0.645 - 1.208	0.435
SMPD2	**0.619**	**0.433 - 0.884**	**0.008**	0.982	0.756 - 1.274	0.889
SMPD3	0.997	0.851 - 1.168	0.971	0.950	0.848 - 1.064	0.375
SPHK1	1.219	0.981 - 1.515	0.074	1.020	0.888 - 1.171	0.783
SPHK2	0.839	0.556 - 1.266	0.404	0.899	0.680 - 1.189	0.457
UGCG	0.936	0.628 - 1.395	0.746	0.999	0.749 - 1.334	0.997
UGT8	0.901	0.766 - 1.061	0.211	1.035	0.914 - 1.172	0.589
CD14	0.970	0.728 - 1.293	0.837	0.970	0.795 - 1.184	0.764
CD68	**0.824**	**0.717 - 0.947**	**0.006**	0.933	0.771 - 1.129	0.478
CD163	0.990	0.745 - 1.315	0.945	0.997	0.819 - 1.213	0.975
CD3E	0.965	0.817 - 1.140	0.673	0.941	0.842 - 1.052	0.284
IGHG1	0.987	0.919 - 1.060	0.716	0.972	0.926 - 1.021	0.259
IGHM	0.964	0.885 - 1.050	0.397	0.968	0.911 - 1.028	0.285
MS4A1	1.031	0.942 - 1.127	0.508	0.978	0.917 - 1.043	0.495
PTPRC	1.011	0.802 - 1.275	0.923	0.980	0.836 - 1.149	0.801

Histology (serous vs. non-serous) was encoded as “0” for serous and “1” for non-serous; FIGO stage (II vs. III vs. IV) was encoded as “2” for FIGO stage II, “3” for FIGO stage III and “4” for FIGO stage IV; Grading (Grade 1 and 2 vs. 3) was encoded as “0” for Grade 1 and 2 and “1” for Grade 3; Peritoneal carcinomatosis (no vs. yes) was encoded as “0” for no and “1” for yes; Residual disease (no vs. yes) was encoded as “0” for no and “1” for yes. HR, hazard ratio (given per doubling of gene expression where log2 transformed values were used); CI, confidence interval; bold, statistically significant ($p < 0.05$). Given the exploratory nature of the analysis no adjustment for multiple testing has been performed.

Table S5. Comparative analysis of multivariable models (LASSO) for prognostication of OS and PFS. Cross-validated performance assessment of Cox regression models (LASSO) by proportion of explained variation (PEV), concordance index (c-index) and p-value.

	OS			PFS		
	**PEV %**	**c-index**	**P**	**PEV %**	**c-index**	**P**
**Clinics**	**9.07**	**0.700**	**< 0.001**	**14.89**	**0.613**	**< 0.001**
**Sphingo**	1.86	0.587	0.060	-	-	-
**Immune**	-	-	-	-	-	-
**Sphingo + Immune**	**2.82**	**0.604**	**0.025**	-	-	-
**Clinics + Sphingo**	**12.30**	**0.728**	**< 0.001; < 0.001***	-	-	-
**Clinics + Immune**	2.63	0.564	0.051; 0.221*	**14.22**	**0.607**	**< 0.001; 0.019***
**Clinics + Sphingo + Immune**	**16.90**	**0.755**	**< 0.001; < 0.001***	-	-	-

* p-value for added value of Sphingo or Immune or Sphingo + Immune on top of Clinics in bivariable models with cross-validated predictors.

Table S6. Relative importance of individual variables in multivariable models (LASSO) for OS. Within each model, variables are ranked by descending importance as expressed by their absolute standardized regression coefficients. * and ** indicate models that were not statistically significant or could not be built, respectively (Table S5).

**Clinics**				
**Pos.**	**Variables**	**beta**	**HR**	**STDBETA**
**1.**	Peritoneal carcinomatosis	0.970	2.638	0.446
**2.**	Age	0.034	1.034	0.402
**3.**	Histology	0.768	2.155	0.246
**4.**	Grading	0.523	1.687	0.235
**5.**	Residual disease	0.466	1.594	0.207
**6.**	FIGO stage	0.499	1.647	0.179

**Sphingo ***				
**Pos.**	**Variables**	**beta**	**HR**	**STDBETA**
**1.**	LPAR3	-0.076	0.926	-0.203
**2.**	SMPD2	-0.222	0.801	-0.177
**3.**	S1PR5	0.135	1.145	0.164
**4.**	PPAP2B	-0.157	0.854	-0.160
**5.**	SMPD1	-0.228	0.796	-0.145
**6.**	SGPP2	-0.058	0.944	-0.070
**7.**	CERS1	0.032	1.032	0.057
**8.**	LPAR6	0.004	1.004	0.004

**Immune ****				
**Pos.**	**Variables**	**beta**	**HR**	**STDBETA**
**1.**	CD68	-0.032	0.969	-0.047

**Sphingo + Immune**				
**Pos.**	**Variables**	**beta**	**HR**	**STDBETA**
**1.**	CD68	-0.192	0.826	-0.285
**2.**	LPAR3	-0.100	0.904	-0.267
**3.**	SMPD2	-0.285	0.752	-0.227
**4.**	SMPD1	-0.347	0.707	-0.220
**5.**	PPAP2B	-0.202	0.817	-0.206
**6.**	S1PR5	0.160	1.174	0.195
**7.**	SPHK1	0.079	1.082	0.110
**8.**	LPAR6	0.097	1.102	0.091
**9.**	LPAR4	0.045	1.046	0.069
**10.**	PPAP2C	0.038	1.039	0.060
**11.**	SGPP2	-0.039	0.962	-0.047
**12.**	CERS1	0.025	1.025	0.045
**13.**	CERS3	0.017	1.017	0.041
**14.**	CERS2	-0.009	0.991	-0.005

Clinics + Sphingo				
**Pos.**	**Variables**	**beta**	**HR**	**STDBETA**
**1.**	Peritoneal carcinomatosis	1.142	3.132	0.525
**2.**	Age	0.040	1.041	0.476
**3.**	SMPD1	-0.668	0.513	-0.424
**4.**	LPAR3	-0.149	0.862	-0.396
**5.**	PPAP2B	-0.355	0.701	-0.362
**6.**	Residual disease	0.788	2.199	0.349
**7.**	CERS1	0.166	1.181	0.298
**8.**	Histology	0.775	2.171	0.249
**9.**	Grading	0.540	1.715	0.242
**10.**	SMPD2	-0.295	0.745	-0.235
**11.**	PPAP2C	0.147	1.158	0.232
**12.**	CERS6	-0.327	0.721	-0.213
**13.**	CERS5	0.378	1.460	0.212
**14.**	LPAR1	0.173	1.189	0.188
**15.**	LPAR6	0.203	1.225	0.188
**16.**	SGPP1	0.264	1.302	0.175
**17.**	SPHK2	-0.244	0.784	-0.169
**18.**	FIGO stage	0.452	1.572	0.162
**19.**	CD1B	-0.052	0.950	-0.154
**20.**	CERS3	0.044	1.045	0.105
**21.**	S1PR5	0.082	1.085	0.099
**22.**	LPAR2	-0.113	0.893	-0.084
**23.**	SGMS1	-0.062	0.940	-0.066
**24.**	S1PR2	-0.059	0.943	-0.054
**25.**	S1PR3	-0.027	0.973	-0.026
**26.**	SGMS2	0.006	1.006	0.006
**27.**	LPAR5	-0.005	0.995	-0.005
**28.**	CERS2	0.000	1.000	0.000

Clinics + Immune*				
**Pos.**	**Variables**	**beta**	**HR**	**STDBETA**
**1.**	Peritoneal carcinomatosis	1.284	3.612	0.591
**2.**	IGHM	-0.153	0.858	-0.503
**3.**	Age	0.041	1.041	0.484
**4.**	CD68	-0.286	0.752	-0.425
**5.**	MS4A1	0.123	1.131	0.366
**6.**	Histology	1.014	2.755	0.325
**7.**	Grading	0.659	1.933	0.296
**8.**	FIGO stage	0.805	2.237	0.289
**9.**	Residual disease	0.598	1.819	0.265
**10.**	CD163	0.108	1.114	0.111
**11.**	CD14	0.067	1.069	0.065
**12.**	IGHG1	-0.006	0.994	-0.023

Clinics + Sphingo + Immune				
**Pos.**	**Variables**	**beta**	**HR**	**STDBETA**
**1.**	Peritoneal carcinomatosis	1.065	2.901	0.490

**2.**	LPAR3	-0.152	0.859	-0.404
**3.**	CD68	-0.260	0.771	-0.387
**4.**	Age	0.031	1.031	0.366
**5.**	Residual disease	0.619	1.857	0.274
**6.**	SMPD2	-0.308	0.735	-0.245
**7.**	Grading	0.533	1.703	0.239
**8.**	PPAP2B	-0.226	0.798	-0.231
**9.**	Histology	0.690	1.993	0.221
**10.**	PPAP2C	0.119	1.126	0.188
**11.**	SMPD1	-0.284	0.753	-0.180
**12.**	SPHK1	0.118	1.125	0.165
**13.**	FIGO stage	0.449	1.567	0.161
**14.**	LPAR6	0.162	1.176	0.150
**15.**	CERS6	-0.152	0.859	-0.099
**16.**	CERS1	0.053	1.054	0.095
**17.**	CERS3	0.033	1.034	0.080
**18.**	IGHM	-0.022	0.979	-0.071
**19.**	SGPP1	0.106	1.112	0.070
**20.**	LPAR1	0.041	1.042	0.045
**21.**	S1PR2	-0.013	0.987	-0.012
**22.**	S1PR3	-0.010	0.990	-0.010
**23.**	S1PR5	0.000	1.000	0.000

Histology (serous vs. non-serous) was encoded as “0” for serous and “1” for non-serous; FIGO stage (II vs. III vs. IV) was encoded as “2” for FIGO stage II, “3” for FIGO stage III and “4” for FIGO stage IV; Grading (Grade 1 and 2 vs. 3) was encoded as “0” for Grade 1 and 2 and “1” for Grade 3; Peritoneal carcinomatosis (no vs. yes) was encoded as “0” for no and “1” for yes; Residual disease (no vs. yes) was encoded as “0” for no and “1” for yes; beta, regression coefficient (log hazard ratio); HR, hazard ratio; STDBETA, standardize regression coefficients. By multivariate modeling with penalized likelihood, the multivariate-adjusted HR shows the direction and magnitude of prognostic effect if adjusted for other variables. Ridge regression shifts the HR towards the value of 1.0 to avoid overestimation bias and to decrease variance in models with many variables.

Table S7. Relative importance of individual variables in multivariable models (ridge) for OS. Within each model, variables are ranked by descending importance as expressed by their absolute standardized regression coefficients. * indicate models that were statistically not significant (Table 2).

**Clinics**				
**Pos.**	**Variables**	**beta**	**HR**	**STDBETA**
1.	Peritoneal carcinomatosis	0.688	1.990	0.317
2.	Age	0.025	1.026	0.302
3.	Grading	0.471	1.602	0.212
4.	Residual disease	0.467	1.596	0.207
5.	Histology	0.565	1.760	0.181
6.	FIGO stage	0.468	1.596	0.168

**Sphingo**				
**Pos.**	**Variables**	**beta**	**HR**	**STDBETA**
1.	LPAR3	-0.043	0.957	-0.116
2.	SMPD2	-0.141	0.868	-0.113
3.	SMPD1	-0.177	0.838	-0.112
4.	PPAP2B	-0.097	0.907	-0.099
5.	S1PR5	0.078	1.082	0.095
6.	SGPP2	-0.060	0.942	-0.072
7.	CERS1	0.040	1.040	0.071
8.	SPHK1	0.046	1.047	0.064
9.	CD1B	-0.020	0.980	-0.060
10.	CERS2	-0.108	0.898	-0.060
11.	LPAR6	0.063	1.065	0.059
12.	S1PR2	-0.060	0.942	-0.055
13.	ENPP2	-0.050	0.952	-0.053
14.	PPAP2C	0.031	1.031	0.049
15.	SGMS1	-0.047	0.955	-0.049
16.	CERS5	0.079	1.083	0.045
17.	LPAR4	0.029	1.029	0.045
18.	CERS6	-0.067	0.935	-0.044
19.	CERS3	0.017	1.017	0.041
20.	S1PR3	-0.036	0.965	-0.035
21.	SPHK2	-0.048	0.953	-0.033
22.	SGPP1	0.048	1.049	0.032
23.	SGPL1	-0.042	0.959	-0.031
24.	CERK	0.029	1.030	0.024
25.	UGT8	-0.013	0.988	-0.022
26.	ASAH1	0.034	1.035	0.021
27.	NAAA	0.019	1.019	0.017
28.	LPAR5	0.016	1.016	0.016
29.	CERS4	-0.014	0.986	-0.015
30.	S1PR4	0.010	1.010	0.014
31.	SMPD3	0.007	1.007	0.012
32.	CD1D	0.005	1.005	0.007
33.	PPAP2A	-0.006	0.994	-0.006

34.	S1PR1	-0.006	0.994	-0.006
35.	SGMS2	0.005	1.005	0.005
36.	UGCG	-0.002	0.998	-0.002
37.	LPAR2	0.002	1.002	0.001
38.	LPAR1	0.000	1.000	0.000

**Immune**				
**Pos.**	**Variables**	**beta**	**HR**	**STDBETA**
1.	CD68	-0.218	0.804	-0.325
2.	MS4A1	0.080	1.083	0.238
3.	IGHM	-0.048	0.953	-0.158
4.	CD3E	-0.065	0.937	-0.106
5.	CD163	0.102	1.107	0.105
6.	PTPRC	0.081	1.084	0.096
7.	IGHG1	-0.012	0.988	-0.046
8.	CD14	0.028	1.028	0.027

**Sphingo + Immune**				
**Pos.**	**Variables**	**beta**	**HR**	**STDBETA**
1.	CD68	-0.125	0.883	-0.186
2.	SMPD2	-0.200	0.819	-0.159
3.	SMPD1	-0.248	0.780	-0.157
4.	LPAR3	-0.058	0.944	-0.154
5.	S1PR5	0.109	1.116	0.133
6.	PPAP2B	-0.123	0.884	-0.125
7.	SPHK1	0.071	1.073	0.099
8.	LPAR6	0.093	1.097	0.086
9.	CERS1	0.046	1.047	0.082
10.	CERS2	-0.142	0.868	-0.079
11.	S1PR2	-0.083	0.921	-0.076
12.	SGPP2	-0.061	0.941	-0.073
13.	PPAP2C	0.046	1.047	0.072
14.	LPAR4	0.043	1.044	0.067
15.	SGMS1	-0.062	0.940	-0.066
16.	CERS3	0.027	1.028	0.065
17.	ENPP2	-0.061	0.941	-0.065
18.	CD3E	-0.038	0.963	-0.062
19.	CERS5	0.108	1.114	0.061
20.	CERS6	-0.093	0.911	-0.061
21.	CD1B	-0.020	0.980	-0.060
22.	IGHM	-0.017	0.983	-0.057
23.	MS4A1	0.018	1.018	0.053
24.	S1PR4	0.036	1.036	0.051
25.	SGPP1	0.069	1.071	0.046
26.	SPHK2	-0.059	0.943	-0.041
27.	LPAR5	0.040	1.041	0.039
28.	S1PR3	-0.041	0.960	-0.039
29.	CERK	0.042	1.043	0.034
30.	ASAH1	0.053	1.055	0.033

31.	CERS4	-0.031	0.969	-0.033
32.	NAAA	0.037	1.037	0.033
33.	SGPL1	-0.034	0.967	-0.025
34.	CD14	-0.023	0.977	-0.023
35.	UGT8	-0.012	0.988	-0.021
36.	LPAR2	0.025	1.026	0.019
37.	SMPD3	0.010	1.011	0.018
38.	IGHG1	-0.005	0.995	-0.018
39.	PTPRC	0.015	1.015	0.018
40.	CD1D	0.012	1.012	0.016
41.	LPAR1	0.012	1.012	0.013
42.	S1PR1	-0.013	0.987	-0.012
43.	PPAP2A	-0.007	0.993	-0.006
44.	UGCG	0.009	1.009	0.006
45.	CD163	0.005	1.005	0.005
46.	SGMS2	0.001	1.001	0.001

**Clinics + Sphingo**				
**Pos.**	**Variables**	**beta**	**HR**	**STDBETA**
1.	Peritoneal carcinomatosis	0.775	2.171	0.357
2.	Age	0.030	1.030	0.355
3.	SMPD1	-0.486	0.615	-0.308
4.	Residual disease	0.657	1.929	0.291
5.	LPAR3	-0.107	0.899	-0.284
6.	PPAP2B	-0.264	0.768	-0.270
7.	Grading	0.558	1.748	0.251
8.	Histology	0.695	2.003	0.223
9.	CERS5	0.380	1.462	0.213
10.	PPAP2C	0.131	1.140	0.207
11.	CERS1	0.112	1.118	0.200
12.	LPAR6	0.202	1.224	0.188
13.	SMPD2	-0.234	0.791	-0.186
14.	CERS6	-0.279	0.756	-0.182
15.	FIGO stage	0.465	1.592	0.167
16.	SGPP1	0.222	1.249	0.147
17.	S1PR5	0.117	1.124	0.142
18.	SGMS1	-0.128	0.880	-0.135
19.	SPHK2	-0.192	0.826	-0.133
20.	LPAR1	0.118	1.126	0.129
21.	S1PR2	-0.139	0.870	-0.128
22.	CD1B	-0.039	0.962	-0.117
23.	CERS3	0.047	1.048	0.112
24.	CERS2	-0.175	0.840	-0.097
25.	LPAR5	-0.095	0.910	-0.094
26.	SGMS2	0.076	1.079	0.080
27.	S1PR3	-0.077	0.926	-0.075
28.	ENPP2	-0.066	0.936	-0.070
29.	SPHK1	0.049	1.050	0.069
30.	SMPD3	0.039	1.039	0.068
31.	CERK	0.071	1.074	0.058

32.	UGCG	-0.075	0.927	-0.053
33.	UGT8	-0.030	0.971	-0.051
34.	S1PR1	0.048	1.049	0.044
35.	PPAP2A	-0.049	0.952	-0.043
36.	LPAR4	0.027	1.028	0.042
37.	CERS4	0.037	1.037	0.039
38.	SGPL1	-0.054	0.948	-0.039
39.	LPAR2	-0.045	0.956	-0.034
40.	NAAA	0.027	1.028	0.025
41.	ASAH1	0.029	1.030	0.018
42.	CD1D	-0.011	0.989	-0.015
43.	SGPP2	-0.010	0.990	-0.012
44.	S1PR4	0.007	1.007	0.010

**Clinics + Immune**				
**Pos.**	**Variables**	**beta**	**HR**	**STDBETA**
1.	Peritoneal carcinomatosis	0.967	2.629	0.444
2.	Age	0.034	1.035	0.405
3.	CD68	-0.255	0.775	-0.379
4.	IGHM	-0.098	0.906	-0.325
5.	Grading	0.615	1.849	0.276
6.	MS4A1	0.090	1.094	0.267
7.	Histology	0.818	2.266	0.262
8.	Residual disease	0.579	1.785	0.257
9.	FIGO stage	0.711	2.036	0.255
10.	IGHG1	-0.024	0.976	-0.092
11.	CD163	0.085	1.089	0.088
12.	CD14	0.082	1.085	0.080
13.	PTPRC	0.011	1.011	0.013
14.	CD3E	0.003	1.003	0.005

**Clinics + Sphingo + Immune**				
**Pos.**	**Variables**	**beta**	**HR**	**STDBETA**
1.	Peritoneal carcinomatosis	0.834	2.302	0.383
2.	Age	0.029	1.030	0.349
3.	CD68	-0.233	0.792	-0.347
4.	Residual disease	0.669	1.953	0.297
5.	LPAR3	-0.109	0.897	-0.290
6.	SMPD1	-0.428	0.652	-0.272
7.	Grading	0.594	1.812	0.267
8.	Histology	0.779	2.179	0.250
9.	PPAP2B	-0.240	0.786	-0.245
10.	SMPD2	-0.291	0.748	-0.232
11.	PPAP2C	0.137	1.147	0.216
12.	LPAR6	0.221	1.248	0.206
13.	CERS6	-0.296	0.744	-0.193
14.	FIGO stage	0.501	1.650	0.180
15.	SPHK1	0.117	1.124	0.163
16.	LPAR1	0.149	1.161	0.162

17.	CERS1	0.089	1.093	0.159
18.	SGPP1	0.226	1.254	0.150
19.	IGHM	-0.045	0.956	-0.147
20.	CD3E	-0.084	0.920	-0.137
21.	CERS3	0.055	1.056	0.131
22.	SGMS1	-0.122	0.885	-0.128
23.	S1PR2	-0.137	0.872	-0.126
24.	S1PR5	0.102	1.107	0.123
25.	CERS5	0.217	1.242	0.122
26.	S1PR4	0.084	1.087	0.120
27.	MS4A1	0.040	1.041	0.119
28.	SPHK2	-0.148	0.863	-0.102
29.	LPAR4	0.058	1.059	0.089
30.	S1PR3	-0.088	0.915	-0.086
31.	CD14	-0.077	0.926	-0.076
32.	CERS2	-0.126	0.881	-0.070
33.	SMPD3	0.037	1.038	0.065
34.	CD1B	-0.021	0.979	-0.063
35.	UGT8	-0.031	0.969	-0.054
36.	SGMS2	0.051	1.052	0.053
37.	PPAP2A	-0.060	0.942	-0.052
38.	ENPP2	-0.046	0.955	-0.049
39.	ASAH1	0.064	1.067	0.040
40.	NAAA	0.043	1.044	0.038
41.	PTPRC	-0.032	0.968	-0.038
42.	CERK	0.045	1.046	0.037
43.	SGPP2	0.031	1.031	0.037
44.	IGHG1	-0.009	0.991	-0.035
45.	CD163	0.033	1.034	0.034
46.	LPAR5	-0.028	0.973	-0.027
47.	S1PR1	0.023	1.023	0.021
48.	UGCG	-0.026	0.975	-0.018
49.	CD1D	0.010	1.011	0.014
50.	SGPL1	-0.012	0.988	-0.009
51.	CERS4	-0.006	0.994	-0.007
52.	LPAR2	0.000	1.000	0.000

Histology (serous vs. non-serous) was encoded as “0” for serous and “1” for non-serous; FIGO stage (II vs. III vs. IV) was encoded as “2” for FIGO stage II, “3” for FIGO stage III and “4” for FIGO stage IV; Grading (Grade 1 and 2 vs. 3) was encoded as “0” for Grade 1 and 2 and “1” for Grade 3; Peritoneal carcinomatosis (no vs. yes) was encoded as “0” for no and “1” for yes; Residual disease (no vs. yes) was encoded as “0” for no and “1” for yes; beta, regression coefficient (log hazard ratio); HR, hazard ratio; STDBETA, standardize regression coefficients. By multivariate modeling with penalized likelihood, the multivariate-adjusted HR shows the direction and magnitude of prognostic effect if adjusted for other variables. Ridge regression shifts the HR towards the value of 1.0 to avoid overestimation bias and to decrease variance in models with many variables.

Table S8. Comparative analysis of multivariable models (ridge and LASSO) for prediction of response to chemotherapy. Cross-validated performance assessment of logistic ridge regression models by proportion of explained variation (PEV), concordance index (c-index) and p-value.

	ridge			LASSO		
	**PEV %**	**c-index**	**P**	**PEV %**	**c-index**	**P**
**Clinics**	7.26	0.657	<0.001	5.60	0.638	< 0.001
**Sphingo**	-	-	-	4.20	0.601	0.152
**Immune**	1.40	0.550	0.644	1.29	0.538	0.888
**Sphingo + Immune**	-	-	-	3.76	0.598	0.194
**Clinics + Sphingo**	2.83	0.596	0.107; 0.951*	4.55	0.621	0.032; 0.176*
**Clinics + Immune**	8.10	0.667	< 0.001; 0.162*	5.68	0.639	0.006; 0.339*
**Clinics + Sphingo + Immune**	3.15	0.609	0.074; 0.688*	4.58	0.622	0.033; 0.145*

* p-value for added value of Sphingo or Immune or Sphingo + Immune on top of Clinics in bivariable models with cross-validated predictors.

Table S9. Clinicopathological characteristics of patient cohort (n = 67) used for immunohistochemical staining

**Patients (%)**	**67 (100.0)**
Age at diagnosis [years]	
Median (range)	62 (28 - 92)
Progression-free survival [days] (6 missing [9 %])	
Median (range)	394 (0 - 3578)
Number of recurrencies (%)	19 (28.4)
Overall survival [days]	
Median (range)	568 (34 - 3892)
Number of deaths (%)	31 (46.3)
Histology (%)	
Serous and serous-papillary	52 (77.6)
Endometrioid	15 (22.4)
FIGO (%)	
I	12 (17.9)
II	13 (19.4)
III	38 (56.7)
IV	4 (6)
Histological Grading (%)	
1	15 (22.4)
2	21 (31.3)
3	31 (46.3)
Residual disease after initial surgery (%)	
None	26 (38.8)
< 2 cm^3	13 (19.4)
≥ 2 cm^3	28 (41.8)
Type of chemotherapy (%, 1 missing [1.5 %])	
None	13 (19.4)
Platinum-based	49 (73.1)
Other	4 (6.0)
Response to first-line chemotherapy (%, 12 missing [17.9 %])	
No evidense of disease	29 (43.3)
Partial response	12 (17.9)
Stable disease	8 (11.9)
Progressive disease	6 (9.0)

Table S10. The top 200 Affymetrix probe sets co-expressed with *CD68* in monocytes/macrophages. The probe sets were identified using GENEVESTIGATOR and ranked according to the Pearson correlation coefficient (Score).

Rank	Score	Probe set	Gene Symbol	Description
0	0.815	203507_at		
1	0.800	225114_at	AGPS	alkylglycerone phosphate synthase
2	0.797	209649_at	STAM2	signal transducing adaptor molecule 2
3	0.793	222726_s_at	EXOC5	exocyst complex component 5
4	0.792	215548_s_at	SCFD1	sec1 family domain containing 1
5	0.788	223391_at	SGPP1	sphingosine-1-phosphate phosphatase 1
6	0.787	205684_s_at	DENND4C	DENN domain containing 4C
7	0.782	218470_at	YARS2	tyrosyl-tRNA synthetase 2
8	0.778	210980_s_at	ASAH1	N-acylsphingosine amidohydrolase (acid ceramidase) 1
9	0.778	225674_at	BCAP29	B-cell receptor-associated protein 29
10	0.777	224723_x_at	LINC00998	long intergenic non-protein coding RNA 998
11	0.777	218212_s_at	MOCS2	molybdenum cofactor synthesis 2
12	0.777	204342_at	SLC25A24	solute carrier family 25 (mitochondrial carrier; phosphate carrier), member 24
13	0.776	228220_at	FCHO2	FCH domain only 2
14	0.775	213117_at	KLHL9	kelch like family member 9
15	0.775	228707_at	CLDN23	claudin 23
16	0.774	219074_at	TMEM184C	transmembrane protein 184C
17	0.772	205084_at	BCAP29	B-cell receptor-associated protein 29
18	0.771	212698_s_at	SEPT10	septin 10
19	0.771	218694_at	ARMCX1	armadillo repeat containing, X-linked 1
20	0.769	214155_s_at	LARP4	La ribonucleoprotein domain family member 4
21	0.769	214830_at	SLC38A6	solute carrier family 38 member 6
22	0.768	229173_at	KIAA1715	KIAA1715
23	0.768	226510_at	HEATR5A	HEAT repeat containing 5A
24	0.768	204565_at	ACOT13	acyl-CoA thioesterase 13
25	0.765	201403_s_at	MGST3	microsomal glutathione S-transferase 3
26	0.765	204085_s_at	CLN5	ceroid-lipofuscinosis, neuronal 5
27	0.765	222500_at	PPIL1	peptidylprolyl isomerase like 1
28	0.764	201780_s_at	RNF13	ring finger protein 13
29	0.762	226837_at	SPRED1	sprouty related, EVH1 domain containing 1
30	0.762	214934_at	ATP9B	ATPase phospholipid transporting 9B (putative)
31	0.762	218196_at	OSTM1	osteopetrosis associated transmembrane protein 1
32	0.761	223240_at	FBXO8	F-box protein 8
33	0.761	207855_s_at	CLCC1	chloride channel CLIC like 1
34	0.761	202346_at	UBE2K	ubiquitin conjugating enzyme E2K
35	0.760	225847_at	NCEH1	neutral cholesterol ester hydrolase 1
36	0.760	220985_s_at	RNF170	ring finger protein 170
37	0.760	204544_at	HPS5	HPS5, biogenesis of lysosomal organelles complex 2 subunit 2
38	0.760	224392_s_at	OPN3	opsin 3
39	0.760	225580_at	MRPL50	mitochondrial ribosomal protein L50
40	0.759	210540_s_at	B4GALT4	UDP-Gal:betaGlcNAc beta 1,4- galactosyltransferase, polypeptide 4
41	0.758	227268_at	RNFT1	ring finger protein, transmembrane 1
42	0.757	208898_at	ATP6V1D	ATPase H+ transporting V1 subunit D
43	0.757	228418_at	EXOC5	exocyst complex component 5
44	0.757	204700_x_at	DIEXF	digestive organ expansion factor homolog (zebrafish)
45	0.755	228706_s_at	CLDN23	claudin 23
46	0.755	201824_at	RNF14	ring finger protein 14
47	0.755	219037_at	RRP15	ribosomal RNA processing 15 homolog
48	0.754	206584_at	LY96	lymphocyte antigen 96
49	0.753	212568_s_at	DLAT	dihydrolipoamide S-acetyltransferase
50	0.753	224786_at	SCOC	short coiled-coil protein
51	0.753	203211_s_at	MTMR2	myotubularin related protein 2
52	0.753	215726_s_at	CYB5A	cytochrome b5 type A (microsomal)
53	0.751	222388_s_at	RP11-93O14.2,VPS35	VPS35:VPS35 retromer complex component
54	0.751	202823_at	TCEB1	transcription elongation factor B (SIII), polypeptide 1 (15kDa, elongin C)
55	0.751	241734_at	SRFBP1	serum response factor binding protein 1
56	0.751	225045_at	CCDC88A	coiled-coil domain containing 88A
57	0.750	201900_s_at	AKR1A1	aldo-keto reductase family 1, member A1 (aldehyde reductase)
58	0.750	211063_s_at	NCK1	NCK adaptor protein 1
59	0.750	225585_at	RAP2A	RAP2A, member of RAS oncogene family
60	0.749	234982_at	UBR3	ubiquitin protein ligase E3 component n-recognin 3 (putative)
61	0.749	226686_at	AC127391.3,CISD2,LA16c-17H1.3,RP11-453N3.7,RP11-626K17.2	CISD2:CDGSH iron sulfur domain 2
62	0.749	224858_at	ZDHHC5	zinc finger DHHC-type containing 5
63	0.748	219023_at	AP1AR	adaptor related protein complex 1 associated regulatory protein
64	0.748	203646_at	FDX1	ferredoxin 1
65	0.748	218046_s_at	MRPS16	mitochondrial ribosomal protein S16
66	0.747	208667_s_at	ST13	suppression of tumorigenicity 13 (colon carcinoma) (Hsp70 interacting protein)
67	0.747	218172_s_at	DERL1	derlin 1
68	0.747	226184_at	FMNL2	formin like 2
69	0.746	224819_at	TCEAL8	transcription elongation factor A like 8
70	0.746	221079_s_at	METTL2A,METTL2B	METTL2A:methyltransferase like 2A, METTL2B:methyltransferase like 2B
71	0.746	218989_x_at	SLC30A5	solute carrier family 30 (zinc transporter), member 5
72	0.745	224791_at	ASAP1	ArfGAP with SH3 domain, ankyrin repeat and PH domain 1
73	0.745	218823_s_at	KCTD9	potassium channel tetramerization domain containing 9
74	0.745	218073_s_at	NDC1	NDC1 transmembrane nucleoporin
75	0.745	226152_at	TTC7B	tetratricopeptide repeat domain 7B
76	0.744	225406_at	TWSG1	twisted gastrulation BMP signaling modulator 1
77	0.744	203775_at	SLC25A13	solute carrier family 25 (aspartate/glutamate carrier), member 13
78	0.744	208626_s_at	VAT1	vesicle amine transport 1
79	0.744	213061_s_at	NTAN1	N-terminal asparagine amidase
80	0.744	214708_at	SNTB1	syntrophin beta 1
81	0.744	218890_x_at	MRPL35	mitochondrial ribosomal protein L35
82	0.744	225447_at	GPD2	glycerol-3-phosphate dehydrogenase 2

83	0.743	221045_s_at	PER3	period circadian clock 3
84	0.743	214876_s_at	TUBGCP5	tubulin gamma complex associated protein 5
85	0.742	201657_at	ARL1	ADP ribosylation factor like GTPase 1
86	0.742	227940_at	AC016747.3	
87	0.742	214751_at	ZNF468	zinc finger protein 468
88	0.742	224881_at	VKORC1L1	vitamin K epoxide reductase complex subunit 1 like 1
89	0.742	225378_at	VPS37A	vacuolar protein sorting 37 homolog A (S. cerevisiae)
90	0.742	229851_s_at	C11orf54	chromosome 11 open reading frame 54
91	0.741	225943_at	NLN	neurolysin
92	0.741	222735_at	TMEM38B	transmembrane protein 38B
93	0.741	202381_at	ADAM9	ADAM metallopeptidase domain 9
94	0.741	224983_at	SCARB2	scavenger receptor class B member 2
95	0.740	212887_at	SEC23A	Sec23 homolog A, COPII coat complex component
96	0.739	219362_at	NAA35	N(alpha)-acetyltransferase 35, NatC auxiliary subunit
97	0.739	244661_at	SOAT1	sterol O-acyltransferase 1
98	0.739	232349_x_at	DCAF6	DDB1 and CUL4 associated factor 6
99	0.739	218049_s_at	MRPL13	mitochondrial ribosomal protein L13
100	0.738	212453_at	KIF1BP	KIF1 binding protein
101	0.738	203039_s_at	NDUFS1	NADH:ubiquinone oxidoreductase core subunit S1
102	0.738	225084_at	EXOC5	exocyst complex component 5
103	0.738	223404_s_at	TRMT1L	tRNA methyltransferase 1 like
104	0.738	213530_at	RAB3GAP1	RAB3 GTPase activating protein catalytic subunit 1
105	0.738	207233_s_at	MITF	microphthalmia-associated transcription factor
106	0.737	229810_at	RSPH3	radial spoke 3 homolog (Chlamydomonas)
107	0.737	222603_at	ERMP1	endoplasmic reticulum metallopeptidase 1
108	0.737	208666_s_at	ST13	suppression of tumorigenicity 13 (colon carcinoma) (Hsp70 interacting protein)
109	0.737	214193_s_at	DIEXF	digestive organ expansion factor homolog (zebrafish)
110	0.737	203584_at	EMC2	ER membrane protein complex subunit 2
111	0.737	223071_at	IER3IP1	immediate early response 3 interacting protein 1
112	0.736	224896_s_at	TTL	tubulin tyrosine ligase
113	0.736	235394_at	PLAA	phospholipase A2 activating protein
114	0.736	235387_at	GSTCD	glutathione S-transferase C-terminal domain containing
115	0.736	212500_at	ADO	2-aminoethanethiol (cysteamine) dioxygenase
116	0.736	202359_s_at	SNX19	sorting nexin 19
117	0.735	225039_at	RPE	ribulose-5-phosphate-3-epimerase
118	0.735	215438_x_at	GSPT1	G1 to S phase transition 1
119	0.735	204142_at	ENOSF1	enolase superfamily member 1
120	0.734	203920_at	NR1H3	nuclear receptor subfamily 1 group H member 3
121	0.734	221561_at	SOAT1	sterol O-acyltransferase 1
122	0.734	227813_at	THAP6	THAP domain containing 6
123	0.734	224908_s_at	TTL	tubulin tyrosine ligase
124	0.734	202893_at	UNC13B	unc-13 homolog B (C. elegans)
125	0.733	214293_at	SEPT11	septin 11
126	0.733	218538_s_at	MRS2	MRS2, magnesium transporter
127	0.733	223294_at	PBDC1	polysaccharide biosynthesis domain containing 1
128	0.733	217777_s_at	HACD3	3-hydroxyacyl-CoA dehydratase 3
129	0.733	226021_at	RDH10	retinol dehydrogenase 10 (all-trans)
130	0.733	205133_s_at	HSPE1,HSPE1-MOB4	HSPE1:heat shock protein family E (Hsp10) member 1, HSPE1-MOB4:HSPE1-MOB4 readthrough
131	0.733	217908_s_at	DCAF6	DDB1 and CUL4 associated factor 6
132	0.732	226438_at	SNTB1	syntrophin beta 1
133	0.732	223433_at	YAE1D1	Yae1 domain containing 1
134	0.732	1554018_at	GPNMB	glycoprotein nmb
135	0.732	218185_s_at	ARMC1	armadillo repeat containing 1
136	0.731	202246_s_at	CDK4	cyclin-dependent kinase 4
137	0.731	222654_at	IMPAD1	inositol monophosphatase domain containing 1
138	0.731	219006_at	NDUFAF4	NADH:ubiquinone oxidoreductase complex assembly factor 4
139	0.731	213838_at	NOL7	nucleolar protein 7
140	0.731	37943_at	ZFYVE26	zinc finger FYVE-type containing 26
141	0.730	212709_at	NUP160	nucleoporin 160kDa
142	0.730	226909_at	ZNF518B	zinc finger protein 518B
143	0.730	234295_at	DBR1	debranching RNA lariats 1
144	0.730	203042_at	LAMP2	lysosomal associated membrane protein 2
145	0.730	211630_s_at	GSS	glutathione synthetase
146	0.729	212038_s_at	VDAC1	voltage dependent anion channel 1
147	0.729	235074_at	SPRED1	sprouty related, EVH1 domain containing 1
148	0.729	218547_at	DHDDS	dehydrodolichyl diphosphate synthase subunit
149	0.729	219386_s_at	SLAMF8	SLAM family member 8
150	0.729	213149_at	DLAT	dihydrolipoamide S-acetyltransferase
151	0.729	222613_at	C12orf4	chromosome 12 open reading frame 4
152	0.729	218098_at	ARFGEF2	ADP ribosylation factor guanine nucleotide exchange factor 2
153	0.728	212959_s_at	GNPTAB	N-acetylglucosamine-1-phosphate transferase, alpha and beta subunits
154	0.728	223465_at	COL4A3BP	collagen type IV alpha 3 binding protein
155	0.728	1555905_a_at	TCAIM	T-cell activation inhibitor, mitochondrial
156	0.728	201825_s_at	SCCPDH	saccharopine dehydrogenase (putative)
157	0.728	219032_x_at	OPN3	opsin 3
158	0.728	212522_at	PDE8A	phosphodiesterase 8A
159	0.727	228073_at	NANP	N-acetylneuraminic acid phosphatase
160	0.727	211569_s_at	HADH	hydroxyacyl-CoA dehydrogenase
161	0.727	222466_s_at	MRPL42	mitochondrial ribosomal protein L42
162	0.727	235019_at	CPM	carboxypeptidase M
163	0.726	218254_s_at	SAR1B	secretion associated Ras related GTPase 1B
164	0.726	243252_at		
165	0.726	212981_s_at	TCAF1	TRPM8 channel-associated factor 1
166	0.726	221524_s_at	RRAGD	Ras related GTP binding D
167	0.726	234304_s_at	IPO11	importin 11
168	0.726	201873_s_at	ABCE1	ATP binding cassette subfamily E member 1
169	0.726	223040_at	NAA20	N(alpha)-acetyltransferase 20, NatB catalytic subunit
170	0.726	209448_at	HTATIP2	HIV-1 Tat interactive protein 2
171	0.726	222642_s_at	TMEM33	transmembrane protein 33

172	0.725	202297_s_at	RER1	retention in endoplasmic reticulum sorting receptor 1
173	0.725	212247_at	NUP205	nucleoporin 205kDa
174	0.725	239346_at	GTF2H3	general transcription factor IIH subunit 3
175	0.725	204262_s_at	ADCK3,PSEN2	ADCK3:aarF domain containing kinase 3, PSEN2:presenilin 2
176	0.725	226066_at	MITF	microphthalmia-associated transcription factor
177	0.725	202345_s_at	FABP5,FABP5P1,FABP5P11,FABP5P2,FABP5P7	FABP5:fatty acid binding protein 5, FABP5P1:fatty acid binding protein 5 pseudogene 1, FABP5P11:fatty acid binding protein 5 pseudogene 11, FABP5P2:fatty acid binding protein 5 pseudogene 2, FABP5P7:fatty acid binding protein 5 pseudogene 7
178	0.725	1558292_s_at	PIGW	phosphatidylinositol glycan anchor biosynthesis class W
179	0.725	210825_s_at	PEBP1	phosphatidylethanolamine binding protein 1
180	0.725	201307_at	SEPT11	septin 11
181	0.725	212333_at	FAM98A	family with sequence similarity 98 member A
182	0.725	203100_s_at	CDYL	chromodomain protein, Y-like
183	0.724	218118_s_at	TIMM23	translocase of inner mitochondrial membrane 23 homolog (yeast)
184	0.724	203008_x_at	TXNDC9	thioredoxin domain containing 9
185	0.724	220643_s_at	FAIM	Fas apoptotic inhibitory molecule
186	0.724	238465_at	SETD9	SET domain containing 9
187	0.724	231839_at	PDE12	phosphodiesterase 12
188	0.724	219933_at	GLRX2	glutaredoxin 2
189	0.724	203970_s_at	PEX3	peroxisomal biogenesis factor 3
190	0.724	207843_x_at	CYB5A	cytochrome b5 type A (microsomal)
191	0.723	202706_s_at	UMPS	uridine monophosphate synthetase
192	0.723	203791_at	DMXL1	Dmx like 1
193	0.723	208858_s_at	ESYT1	extended synaptotagmin like protein 1
194	0.723	225384_at	DOCK7	dedicator of cytokinesis 7
195	0.723	202428_x_at	DBI	diazepam binding inhibitor (GABA receptor modulator, acyl-CoA binding protein)
196	0.723	223675_s_at	VEZT	vezatin, adherens junctions transmembrane protein
197	0.723	201659_s_at	ARL1	ADP ribosylation factor like GTPase 1
198	0.723	219048_at	PIGN	phosphatidylinositol glycan anchor biosynthesis class N
199	0.723	201624_at	DARS	aspartyl-tRNA synthetase
200	0.722	208121_s_at	PTPRO	protein tyrosine phosphatase, receptor type O

Table S11. The top 200 Affymetrix probe sets co-expressed with *SMPD1* in monocytes/macrophages. The probe sets were identified using GENEVESTIGATOR and ranked according to the Pearson correlation coefficient (Score).

Rank	Score	Probe set	Gene Symbol	Description
0	0.867	209420_s_at	SMPD1	sphingomyelin phosphodiesterase 1
1	0.846	222817_at	HSD3B7	hydroxy-delta-5-steroid dehydrogenase, 3 beta- and steroid delta-isomerase 7
2	0.843	210589_s_at	GBA	glucosidase, beta, acid
3	0.839	239287_at		
4	0.835	217757_at	A2M	alpha-2-macroglobulin
5	0.833	205738_s_at	FABP3	fatty acid binding protein 3
6	0.832	228559_at		
7	0.832	208626_s_at	VAT1	vesicle amine transport 1
8	0.829	202862_at	FAH	fumarylacetoacetate hydrolase (fumarylacetoacetase)
9	0.827	201656_at	ITGA6	integrin subunit alpha 6
10	0.826	201415_at	GSS	glutathione synthetase
11	0.826	221872_at	RARRES1	retinoic acid receptor responder (tazarotene induced) 1
12	0.825	225185_at	MRAS	muscle RAS oncogene homolog
13	0.825	204027_s_at	METTL1	methyltransferase like 1
14	0.825	238440_at	CLYBL	citrate lyase beta like
15	0.824	213553_x_at		
16	0.824	223249_at	CLDN12	claudin 12
17	0.823	227036_at	RASAL2	RAS protein activator like 2
18	0.820	205379_at	CBR3	carbonyl reductase 3
19	0.820	209321_s_at	ADCY3	adenylate cyclase 3
20	0.819	219506_at	C1orf54	chromosome 1 open reading frame 54
21	0.819	238654_at	VSIG10L	V-set and immunoglobulin domain containing 10 like
22	0.817	221245_s_at	FZD5	frizzled class receptor 5
23	0.817	218547_at	DHDDS	dehydrodolichyl diphosphate synthase subunit
24	0.816	224707_at	CYSTM1	cysteine rich transmembrane module containing 1
25	0.816	210825_s_at	PEBP1	phosphatidylethanolamine binding protein 1
26	0.815	200766_at	CTSD	cathepsin D
27	0.815	200862_at	DHCR24	24-dehydrocholesterol reductase
28	0.813	227100_at	B3GLCT	beta 3-glucosyltransferase
29	0.813	228707_at	CLDN23	claudin 23
30	0.812	209365_s_at	ECM1	extracellular matrix protein 1
31	0.811	203608_at	ALDH5A1	aldehyde dehydrogenase 5 family member A1
32	0.811	204416_x_at	APOC1	apolipoprotein C-I
33	0.811	221620_s_at	APOO	apolipoprotein O
34	0.811	213062_at	NTAN1	N-terminal asparagine amidase
35	0.810	1558685_a_at	CH17-340M24.3	uncharacterized protein BC009467
36	0.810	212737_at	GM2A	GM2 ganglioside activator
37	0.808	230151_at	SPRYD7	SPRY domain containing 7
38	0.808	205225_at	ESR1	estrogen receptor 1
39	0.807	217772_s_at	MTCH2	mitochondrial carrier 2
40	0.807	205353_s_at	PEBP1P2	phosphatidylethanolamine binding protein 1 pseudogene 2
41	0.807	231835_at	FAM213B	family with sequence similarity 213 member B
42	0.806	1553787_at	C11orf45	chromosome 11 open reading frame 45
43	0.806	203072_at	MYO1E	myosin IE
44	0.806	204142_at	ENOSF1	enolase superfamily member 1
45	0.806	213861_s_at	METTL21B	methyltransferase like 21B
46	0.805	226066_at	MITF	microphthalmia-associated transcription factor
47	0.805	221266_s_at	DCSTAMP	dendrocyte expressed seven transmembrane protein
48	0.805	201387_s_at	UCHL1	ubiquitin C-terminal hydrolase L1
49	0.804	226129_at	FAM83H	family with sequence similarity 83 member H
50	0.804	205194_at	PSPH	phosphoserine phosphatase
51	0.804	206102_at	GINS1	GINS complex subunit 1 (Psf1 homolog)
52	0.803	223748_at	SLC4A11	solute carrier family 4, sodium borate transporter, member 11
53	0.803	218542_at	CEP55	centrosomal protein 55kDa
54	0.803	220974_x_at	SFXN3	sideroflexin 3
55	0.803	206298_at	ARHGAP22	Rho GTPase activating protein 22
56	0.802	209395_at	CHI3L1	chitinase 3 like 1
57	0.802	202847_at	PCK2	phosphoenolpyruvate carboxykinase 2, mitochondrial
58	0.802	223120_at	FUCA2	fucosidase, alpha-L- 2, plasma
59	0.801	218763_at	STX18	syntaxin 18
60	0.801	204984_at	GPC4	glypican 4
61	0.801	225554_s_at	ANAPC7	anaphase promoting complex subunit 7
62	0.801	208969_at	NDUFA9	NADH:ubiquinone oxidoreductase subunit A9
63	0.801	227556_at	NME7	NME/NM23 family member 7
64	0.801	224689_at	MANBAL	mannosidase beta like
65	0.801	228557_at	L3MBTL4	l(3)mbt-like 4 (Drosophila)
66	0.799	200832_s_at	SCD	stearoyl-CoA desaturase (delta-9-desaturase)
67	0.797	41660_at	CELSR1	cadherin EGF LAG seven-pass G-type receptor 1
68	0.797	226722_at	FAM20C	family with sequence similarity 20 member C
69	0.797	225545_at	EEF2K	eukaryotic elongation factor 2 kinase
70	0.797	212923_s_at	PXDC1	PX domain containing 1
71	0.796	226089_at	RABL3	RAB, member of RAS oncogene family-like 3
72	0.795	212333_at	FAM98A	family with sequence similarity 98 member A
73	0.795	1553153_at	ATP6V0D2	ATPase H+ transporting V0 subunit d2
74	0.794	226152_at	TTC7B	tetratricopeptide repeat domain 7B
75	0.794	235068_at	ZDHHC21	zinc finger DHHC-type containing 21
76	0.794	203920_at	NR1H3	nuclear receptor subfamily 1 group H member 3
77	0.794	1569403_at		
78	0.794	203211_s_at	MTMR2	myotubularin related protein 2
79	0.793	230676_s_at	RP11-293I14.2	
80	0.793	217869_at	HSD17B12	hydroxysteroid (17-beta) dehydrogenase 12
81	0.793	226435_at	PAPLN	papilin, proteoglycan-like sulfated glycoprotein
82	0.793	222868_s_at	IL18BP	interleukin 18 binding protein
83	0.792	225074_at	RAB2B	RAB2B, member RAS oncogene family
84	0.792	223253_at	EPDR1	ependymin related 1
85	0.792	205339_at	STIL	SCL/TAL1 interrupting locus

86	0.791	206554_x_at	SETMAR	SET domain and mariner transposase fusion gene
87	0.791	219648_at	MREG	melanoregulin
88	0.791	211470_s_at	SULT1C2	sulfotransferase family 1C member 2
89	0.790	224663_s_at	CFL2	cofilin 2
90	0.789	200665_s_at	SPARC	secreted protein acidic and cysteine rich
91	0.789	222870_s_at	B3GNT2	UDP-GlcNAc:betaGal beta-1,3-N-acetylglucosaminyltransferase 2
92	0.789	225783_at	UBE2F	ubiquitin conjugating enzyme E2F (putative)
93	0.789	205273_s_at	PITRM1	pitrilysin metallopeptidase 1
94	0.788	202893_at	UNC13B	unc-13 homolog B (C. elegans)
95	0.788	217225_x_at	NOMO1	NODAL modulator 1
96	0.788	202428_x_at	DBI	diazepam binding inhibitor (GABA receptor modulator, acyl-CoA binding protein)
97	0.787	226855_at	PDP2	pyruvate dehyrogenase phosphatase catalytic subunit 2
98	0.787	200837_at	BCAP31	B-cell receptor-associated protein 31
99	0.786	200757_s_at	CALU	calumenin
100	0.786	225478_at	MFHAS1	malignant fibrous histiocytoma amplified sequence 1
101	0.786	227853_at	PLBD2	phospholipase B domain containing 2
102	0.786	208898_at	ATP6V1D	ATPase H+ transporting V1 subunit D
103	0.785	1569157_s_at	ZNF846	zinc finger protein 846
104	0.785	214170_x_at	FH	fumarate hydratase
105	0.784	208906_at	HNRNPUL2-BSCL2,BSCL2	HNRNPUL2-BSCL2:HNRNPUL2-BSCL2 readthrough (NMD candidate), BSCL2:Berardinelli-Seip congenital lipodystrophy 2 (seipin)
106	0.784	220744_s_at	IFT122	intraflagellar transport 122
107	0.784	204173_at	MYL6B	myosin light chain 6B
108	0.784	214710_s_at	CCNB1	cyclin B1
109	0.784	219386_s_at	SLAMF8	SLAM family member 8
110	0.784	223594_at	TMEM117	transmembrane protein 117
111	0.784	208799_at	PSMB5	proteasome subunit beta 5
112	0.784	223061_at	CHID1	chitinase domain containing 1
113	0.784	204360_s_at	NAGLU	N-acetylglucosaminidase, alpha
114	0.783	224160_s_at	ACAD9	acyl-CoA dehydrogenase family member 9
115	0.783	225128_at	KDELC2	KDEL motif containing 2
116	0.782	200661_at	CTSA	cathepsin A
117	0.782	203454_s_at	ATOX1	antioxidant 1 copper chaperone
118	0.782	218823_s_at	KCTD9	potassium channel tetramerization domain containing 9
119	0.782	208079_s_at	AURKA	aurora kinase A
120	0.782	206352_s_at	PEX10	peroxisomal biogenesis factor 10
121	0.782	219431_at	ARHGAP10	Rho GTPase activating protein 10
122	0.781	208813_at	GOT1	glutamic-oxaloacetic transaminase 1, soluble
123	0.781	1555781_at	PQLC2	PQ loop repeat containing 2
124	0.781	213010_at	PRKCDBP	protein kinase C delta binding protein
125	0.781	232101_s_at	PIGN	phosphatidylinositol glycan anchor biosynthesis class N
126	0.781	218694_at	ARMCX1	armadillo repeat containing, X-linked 1
127	0.780	216574_s_at	RPEL1	ribulose-5-phosphate-3-epimerase-like 1
128	0.780	226795_at	LRCH1	leucine-rich repeats and calponin homology (CH) domain containing 1
129	0.780	229025_s_at	IMMP1L	inner mitochondrial membrane peptidase subunit 1
130	0.779	232065_x_at	CENPL	centromere protein L
131	0.779	208858_s_at	ESYT1	extended synaptotagmin like protein 1
132	0.779	202246_s_at	CDK4	cyclin-dependent kinase 4
133	0.779	202427_s_at	MPC2	mitochondrial pyruvate carrier 2
134	0.779	205801_s_at	RASGRP3	RAS guanyl releasing protein 3
135	0.778	208168_s_at	CHIT1	chitinase 1
136	0.778	48106_at	SLC48A1	solute carrier family 48 member 1
137	0.778	217913_at	VPS4A	vacuolar protein sorting 4 homolog A (S. cerevisiae)
138	0.778	216862_s_at	CMC4	C-x(9)-C motif containing 4
139	0.778	228198_s_at		
140	0.778	37152_at	PPARD	peroxisome proliferator activated receptor delta
141	0.778	1553706_at	HTRA4	HtrA serine peptidase 4
142	0.778	205618_at	PRRG1	proline rich Gla (G-carboxyglutamic acid) 1
143	0.778	201125_s_at	ITGB5	integrin subunit beta 5
144	0.777	202800_at	SLC1A3	solute carrier family 1 member 3
145	0.777	204986_s_at	TAOK2	TAO kinase 2
146	0.776	205352_at	SERPINI1	serpin peptidase inhibitor, clade I (neuroserpin), member 1
147	0.776	208950_s_at	ALDH7A1P1	aldehyde dehydrogenase 7 family member A1 pseudogene 1
148	0.776	202737_s_at	LSM4	LSM4 homolog, U6 small nuclear RNA and mRNA degradation associated
149	0.776	221069_s_at	TACO1	translational activator of mitochondrially encoded cytochrome c oxidase I
150	0.776	221504_s_at	ATP6V1H	ATPase H+ transporting V1 subunit H
151	0.776	227863_at	IFITM10	interferon induced transmembrane protein 10
152	0.775	218942_at	PIP4K2C	phosphatidylinositol-5-phosphate 4-kinase, type II, gamma
153	0.775	222877_at	NRP2	neuropilin 2
154	0.775	218246_at	MUL1	mitochondrial E3 ubiquitin protein ligase 1
155	0.774	201013_s_at	PAICS	phosphoribosylaminoimidazole carboxylase; phosphoribosylaminoimidazolesuccinocarboxamide synthase
156	0.774	212686_at	PPM1H	protein phosphatase, Mg2+/Mn2+ dependent 1H
157	0.774	202154_x_at	TUBB3,RP11-566K11.2	TUBB3:tubulin beta 3 class III
158	0.774	218729_at	LXN	latexin
159	0.774	201519_at	TOMM70A	
160	0.774	222354_at	F11R	F11 receptor
161	0.774	221584_s_at	KCNMA1	potassium calcium-activated channel subfamily M alpha 1
162	0.774	209608_s_at	ACAT2	acetyl-CoA acetyltransferase 2
163	0.774	230972_at	ANKRD9	ankyrin repeat domain 9
164	0.773	214934_at	ATP9B	ATPase phospholipid transporting 9B (putative)
165	0.773	220178_at	MFSD12	major facilitator superfamily domain containing 12
166	0.772	224735_at	CYB561A3	cytochrome b561 family member A3
167	0.772	217050_at		
168	0.772	203159_at	GLS	glutaminase
169	0.772	212296_at	PSMD14	proteasome 26S subunit, non-ATPase 14
170	0.772	218219_s_at	LANCL2	LanC like 2
171	0.771	218662_s_at	NCAPG	non-SMC condensin I complex subunit G
172	0.771	225325_at	MFSD6	major facilitator superfamily domain containing 6
173	0.771	202908_at	WFS1	wolframin ER transmembrane glycoprotein

174	0.771	218503_at	FOCAD	focadhesin
175	0.770	213454_at	APITD1	apoptosis-inducing, TAF9-like domain 1
176	0.770	202605_at	GUSB	glucuronidase, beta
177	0.770	210519_s_at	NQO1	NAD(P)H dehydrogenase, quinone 1
178	0.769	225081_s_at	CDCA7L	cell division cycle associated 7 like
179	0.769	201850_at	CAPG	capping actin protein, gelsolin like
180	0.769	201771_at	SCAMP3	secretory carrier membrane protein 3
181	0.769	228073_at	NANP	N-acetylneuraminic acid phosphatase
182	0.768	224462_s_at	CHCHD6	coiled-coil-helix-coiled-coil-helix domain containing 6
183	0.768	214138_at	ZNF79	zinc finger protein 79
184	0.768	220643_s_at	FAIM	Fas apoptotic inhibitory molecule
185	0.768	218632_at	HECTD3	HECT domain containing E3 ubiquitin protein ligase 3
186	0.768	218407_x_at	NENF	neudesin neurotrophic factor
187	0.768	217925_s_at	C6orf106	chromosome 6 open reading frame 106
188	0.768	230774_at	PTGR2	prostaglandin reductase 2
189	0.768	1557260_a_at	ZNF382	zinc finger protein 382
190	0.767	203775_at	SLC25A13	solute carrier family 25 (aspartate/glutamate carrier), member 13
191	0.767	204671_s_at	ANKRD6	ankyrin repeat domain 6
192	0.767	208837_at	TMED3	transmembrane p24 trafficking protein 3
193	0.767	200985_s_at	CD59	CD59 molecule
194	0.767	225578_at	MZT1	mitotic spindle organizing protein 1
195	0.767	1556314_a_at		
196	0.767	200903_s_at	AHCY	adenosylhomocysteinase
197	0.766	202461_at	EIF2B2	eukaryotic translation initiation factor 2B subunit beta
198	0.766	219765_at	ZNF329	zinc finger protein 329
199	0.766	224444_s_at		
200	0.766	225752_at	NIPA1	non imprinted in Prader-Willi/Angelman syndrome 1

Table S12. The top 10 sphingolipid/lysophosphatidate/immune signature-linked neoplasms identified by GENEVESTIGATOR for the independent patient cohort (n = 19). GENEVESTIGATOR nomenclature is used for malignant disorders. The top 10 results are shown; the ranking is based on the corresponding GENEVESTIGATOR-based relative similarity (Rel. Similarity). Tissue type, study number, and reference(s) annotating the corresponding studies are indicated.

Position	Study	Tissue type	Rel. Similarity	Study Number	Reference
1	Serous cystadenocarcinoma, NOS	Ovary	1.709	GSE2109,<br>GSE12172,<br>GSE20565,<br>GSE19352,<br>GSE36668,<br>GSE63885,<br>GSE32062	part in [1, 2]<br>[3]<br>[4]<br>[5]<br>[6]<br>[7, 8]<br>[9]
2	Papillary serous cystadenocarcinoma, borderline malignancy	Ovary	1.662	GSE9899	[10]
3	Endometrioid carcinoma	Ovary	1.656	GSE2109,<br>GSE9899,<br>GSE20565,<br>GSE63885	part in [1, 2]<br>[10]<br>[4]<br>[7, 8]
4	Serous cystadenocarcinoma, NOS, unstated behavior	Ovary	1.609	GSE26193	[11]
5	Endometrioid carcinoma, metastatic	Endometrium	1.571	GSE2109	part in [1, 2]
6	Serous cystadenoma, borderline malignancy	Ovary	1.537	GSE2109,<br>GSE12172,<br>GSE36668	part in [1, 2]<br>[3]<br>[6]
7	Papillary serous cystadenocarcinoma	Ovary	1.527	GSE2109<br>GSE9899,<br>GSE18521,<br>GSE14407	part in [1, 2]<br>[10]<br>[12]<br>[13]
8	Papillary serous cystadenocarcinoma, metastatic	Ovary	1.524	GSE2109	part in [1, 2]
9	Clear cell adenocarcinoma, NOS	Ovary	1.518	GSE2109,<br>GSE20565,<br>GSE63885	part in [1, 2]<br>[4]<br>[7, 8]
10	Papillary serous cystadenocarcinoma, metastatic	Peritoneum	1.506	GSE2109,<br>GSE9899	part in [1, 2]<br>[10]

1. Kharma, B., et al., *Utilization of genomic signatures to identify high-efficacy candidate drugs for chemorefractory endometrial cancers.* Int J Cancer, 2013. **133**(9): p. 2234-44.
2. Yamamura, S., et al., *The activated transforming growth factor-beta signaling pathway in peritoneal metastases is a potential therapeutic target in ovarian cancer.* Int J Cancer, 2012. **130**(1): p. 20-8.
3. Anglesio, M.S., et al., *Mutation of ERBB2 provides a novel alternative mechanism for the ubiquitous activation of RAS-MAPK in ovarian serous low malignant potential tumors.* Mol Cancer Res, 2008. **6**(11): p. 1678-90.
4. Meyniel, J.P., et al., *A genomic and transcriptomic approach for a differential diagnosis between primary and secondary ovarian carcinomas in patients with a previous history of breast cancer.* BMC Cancer, 2010. **10**: p. 222.
5. Iorio, E., et al., *Activation of phosphatidylcholine cycle enzymes in human epithelial ovarian cancer cells.* Cancer Res, 2010. **70**(5): p. 2126-35.
6. Elgaaen, B.V., et al., *ZNF385B and VEGFA are strongly differentially expressed in serous ovarian carcinomas and correlate with survival.* PLoS One, 2012. **7**(9): p. e46317.
7. Lisowska, K.M., et al., *Gene expression analysis in ovarian cancer - faults and hints from DNA microarray study.* Front Oncol, 2014. **4**: p. 6.
8. Lisowska, K.M., et al., *Unsupervised analysis reveals two molecular subgroups of serous ovarian cancer with distinct gene expression profiles and survival.* J Cancer Res Clin Oncol, 2016. **142**(6): p. 1239-52.
9. Yoshihara, K., et al., *High-risk ovarian cancer based on 126-gene expression signature is uniquely characterized by downregulation of antigen presentation pathway.* Clin Cancer Res, 2012. **18**(5): p. 1374-85.
10. Tothill, R.W., et al., *Novel molecular subtypes of serous and endometrioid ovarian cancer linked to clinical outcome.* Clin Cancer Res, 2008. **14**(16): p. 5198-208.
11. Mateescu, B., et al., *miR-141 and miR-200a act on ovarian tumorigenesis by controlling oxidative stress response.* Nat Med, 2011. **17**(12): p. 1627-35.
12. Mok, S.C., et al., *A gene signature predictive for outcome in advanced ovarian cancer identifies a survival factor: microfibril-associated glycoprotein 2.* Cancer Cell, 2009. **16**(6): p. 521-32.
13. Bowen, N.J., et al., *Gene expression profiling supports the hypothesis that human ovarian surface epithelia are multipotent and capable of serving as ovarian cancer initiating cells.* BMC Med Genomics, 2009. **2**: p. 71.

Table S13. The top 10 sphingolipid/lysophosphatidate/immune signature-linked neoplasms identified by GENEVESTIGATOR for the low risk group of the main patient cohort. GENEVESTIGATOR nomenclature is used for malignant disorders. The top 10 results are shown; the ranking is based on the corresponding GENEVESTIGATOR-based relative similarity (Rel. Similarity). Tissue type, study number, and reference(s) annotating the corresponding studies are indicated.

Position	Study	Tissue type	Rel. Similarity	Study Number	Reference
1	Serous cystadenocarcinoma, NOS	Ovary	1.502	GSE2109,<br>GSE12172,<br>GSE20565,<br>GSE19352,<br>GSE36668,<br>GSE63885,<br>GSE32062	part in [1, 2]<br>[3]<br>[4]<br>[5]<br>[6]<br>[7, 8]<br>[9]
2	Papillary serous cystadenocarcinoma, borderline malignancy	Ovary	1.453	GSE9899	[10]
3	Serous cystadenocarcinoma, NOS, unstated behavior	Ovary	1.429	GSE26193	[11]
4	Endometrioid carcinoma, metastatic	Endometrium	1.419	GSE2109	part in [1, 2]
5	Endometrioid carcinoma	Ovary	1.418	GSE2109,<br>GSE9899,<br>GSE20565,<br>GSE63885	part in [1, 2]<br>[10]<br>[4]<br>[7, 8]
6	Papillary serous cystadenocarcinoma, metastatic	Peritoneum	1.407	GSE2109,<br>GSE9899	part in [1, 2]<br>[10]
7	Papillary serous cystadenocarcinoma, metastatic	Ovary	1.383	GSE2109	part in [1, 2]
8	Papillary serous cystadenocarcinoma	Ovary	1.373	GSE2109,<br>GSE9899,<br>GSE18521,<br>GSE14407	part in [1, 2]<br>[10]<br>[12]<br>[13]
9	Serous cystadenocarcinoma, NOS, metastatic	Ovary	1.359	GSE2109,<br>GSE12172	part in [1, 2]<br>[3]
10	Endometrioid carcinoma	Endometrium	1.351	GSE2109	part in [1, 2]

1. Kharma, B., et al., *Utilization of genomic signatures to identify high-efficacy candidate drugs for chemorefractory endometrial cancers.* Int J Cancer, 2013. **133**(9): p. 2234-44.
2. Yamamura, S., et al., *The activated transforming growth factor-beta signaling pathway in peritoneal metastases is a potential therapeutic target in ovarian cancer.* Int J Cancer, 2012. **130**(1): p. 20-8.
3. Anglesio, M.S., et al., *Mutation of ERBB2 provides a novel alternative mechanism for the ubiquitous activation of RAS-MAPK in ovarian serous low malignant potential tumors.* Mol Cancer Res, 2008. **6**(11): p. 1678-90.
4. Meyniel, J.P., et al., *A genomic and transcriptomic approach for a differential diagnosis between primary and secondary ovarian carcinomas in patients with a previous history of breast cancer.* BMC Cancer, 2010. **10**: p. 222.
5. Iorio, E., et al., *Activation of phosphatidylcholine cycle enzymes in human epithelial ovarian cancer cells.* Cancer Res, 2010. **70**(5): p. 2126-35.
6. Elgaaen, B.V., et al., *ZNF385B and VEGFA are strongly differentially expressed in serous ovarian carcinomas and correlate with survival.* PLoS One, 2012. **7**(9): p. e46317.
7. Lisowska, K.M., et al., *Gene expression analysis in ovarian cancer - faults and hints from DNA microarray study.* Front Oncol, 2014. **4**: p. 6.
8. Lisowska, K.M., et al., *Unsupervised analysis reveals two molecular subgroups of serous ovarian cancer with distinct gene expression profiles and survival.* J Cancer Res Clin Oncol, 2016. **142**(6): p. 1239-52.
9. Yoshihara, K., et al., *High-risk ovarian cancer based on 126-gene expression signature is uniquely characterized by downregulation of antigen presentation pathway.* Clin Cancer Res, 2012. **18**(5): p. 1374-85.
10. Tothill, R.W., et al., *Novel molecular subtypes of serous and endometrioid ovarian cancer linked to clinical outcome.* Clin Cancer Res, 2008. **14**(16): p. 5198-208.
11. Mateescu, B., et al., *miR-141 and miR-200a act on ovarian tumorigenesis by controlling oxidative stress response.* Nat Med, 2011. **17**(12): p. 1627-35.
12. Mok, S.C., et al., *A gene signature predictive for outcome in advanced ovarian cancer identifies a survival factor: microfibril-associated glycoprotein 2.* Cancer Cell, 2009. **16**(6): p. 521-32.
13. Bowen, N.J., et al., *Gene expression profiling supports the hypothesis that human ovarian surface epithelia are multipotent and capable of serving as ovarian cancer initiating cells.* BMC Med Genomics, 2009. **2**: p. 71.

Table S14. The top 10 sphingolipid/lysophosphatidate/immune signature-linked neoplasms identified by GENEVESTIGATOR for the high risk group of the main patient cohort. GENEVESTIGATOR nomenclature is used for malignant disorders. The top 10 results are shown; the ranking is based on the corresponding GENEVESTIGATOR-based relative similarity (Rel. Similarity). Tissue type, study number, and reference(s) annotating the corresponding studies are indicated.

Position	Study	Tissue type	Rel. Similarity	Study Number	Reference
1	Endometrioid carcinoma, metastatic	Endometrium	1.322	GSE2109	part in [1, 2]
2	Meningioma, NOS	Meninges	1.320	GSE4780,<br>GSE9438,<br>GSE16584	[3]<br>[4]<br>[5]
3	Papillary serous cystadenocarcinoma, borderline malignancy	Ovary	1.317	GSE9899	[6]
4	Papillary serous cystadenocarcinoma, metastatic	Ovary	1.309	GSE2109	part in [1, 2]
5	Papillary serous cystadenocarcinoma, metastatic	Peritoneum	1.305	GSE2109,<br>GSE9899	part in [1, 2]<br>[6]
6	Clear cell adenocarcinoma, NOS, metastatic	Kidney	1.288	GSE2109,<br>GSE12606,<br>GSE14378,<br>GSE22541	part in [1, 2]<br>[7]<br>[8]<br>[9]
7	Serous cystadenocarcinoma, NOS	Ovary	1.278	GSE2109,<br>GSE12172,<br>GSE20565,<br>GSE19352,<br>GSE36668,<br>GSE63885,<br>GSE32062	part in [1, 2]<br>[10]<br>[11]<br>[12]<br>[13]<br>[14, 15]<br>[16]
8	Squamous cell carcinoma, NOS	Bronchus or Lung	1.272	GSE2109,<br>GSE12667,<br>GSE19188,<br>GSE18842,<br>GSE37745,<br>GSE50081,<br>GSE43580	part in [1, 2]<br>[17]<br>[18]<br>[19]<br>[20]<br>[21]<br>[22]
9	Endometrioid carcinoma	Endometrium	1.264	GSE2109	part in [1, 2]
10	Endometrioid carcinoma	Ovary	1.259	GSE2109,<br>GSE9899,<br>GSE20565,<br>GSE63885	part in [1, 2]<br>[6]<br>[11]<br>[14, 15]

1. Kharma, B., et al., *Utilization of genomic signatures to identify high-efficacy candidate drugs for chemorefractory endometrial cancers.* Int J Cancer, 2013. **133**(9): p. 2234-44.
2. Yamamura, S., et al., *The activated transforming growth factor-beta signaling pathway in peritoneal metastases is a potential therapeutic target in ovarian cancer.* Int J Cancer, 2012. **130**(1): p. 20-8.
3. Stuart, J.E., et al., *Identification of gene markers associated with aggressive meningioma by filtering across multiple sets of gene expression arrays.* J Neuropathol Exp Neurol, 2011. **70**(1): p. 1-12.
4. Claus, E.B., et al., *Specific genes expressed in association with progesterone receptors in meningioma.* Cancer Res, 2008. **68**(1): p. 314-22.
5. Lee, Y., et al., *Genomic landscape of meningiomas.* Brain Pathol, 2010. **20**(4): p. 751-62.
6. Tothill, R.W., et al., *Novel molecular subtypes of serous and endometrioid ovarian cancer linked to clinical outcome.* Clin Cancer Res, 2008. **14**(16): p. 5198-208.
7. Stickel, J.S., et al., *HLA ligand profiles of primary renal cell carcinoma maintained in metastases.* Cancer Immunol Immunother, 2009. **58**(9): p. 1407-17.
8. Wuttig, D., et al., *Gene signatures of pulmonary metastases of renal cell carcinoma reflect the disease-free interval and the number of metastases per patient.* Int J Cancer, 2009. **125**(2): p. 474-82.
9. Wuttig, D., et al., *CD31, EDNRB and TSPAN7 are promising prognostic markers in clear-cell renal cell carcinoma revealed by genome-wide expression analyses of primary tumors and metastases.* Int J Cancer, 2012. **131**(5): p. E693-704.
10. Anglesio, M.S., et al., *Mutation of ERBB2 provides a novel alternative mechanism for the ubiquitous activation of RAS-MAPK in ovarian serous low malignant potential tumors.* Mol Cancer Res, 2008. **6**(11): p. 1678-90.
11. Meyniel, J.P., et al., *A genomic and transcriptomic approach for a differential diagnosis between primary and secondary ovarian carcinomas in patients with a previous history of breast cancer.* BMC Cancer, 2010. **10**: p. 222.
12. Iorio, E., et al., *Activation of phosphatidylcholine cycle enzymes in human epithelial ovarian cancer cells.* Cancer Res, 2010. **70**(5): p. 2126-35.
13. Elgaaen, B.V., et al., *ZNF385B and VEGFA are strongly differentially expressed in serous ovarian carcinomas and correlate with survival.* PLoS One, 2012. **7**(9): p. e46317.
14. Lisowska, K.M., et al., *Gene expression analysis in ovarian cancer - faults and hints from DNA microarray study.* Front Oncol, 2014. **4**: p. 6.
15. Lisowska, K.M., et al., *Unsupervised analysis reveals two molecular subgroups of serous ovarian cancer with distinct gene expression profiles and survival.* J Cancer Res Clin Oncol, 2016. **142**(6): p. 1239-52.
16. Yoshihara, K., et al., *High-risk ovarian cancer based on 126-gene expression signature is uniquely characterized by downregulation of antigen presentation pathway.* Clin Cancer Res, 2012. **18**(5): p. 1374-85.
17. Ding, L., et al., *Somatic mutations affect key pathways in lung adenocarcinoma.* Nature, 2008. **455**(7216): p. 1069-75.
18. Hou, J., et al., *Gene expression-based classification of non-small cell lung carcinomas and survival prediction.* PLoS One, 2010. **5**(4): p. e10312.
19. Sanchez-Palencia, A., et al., *Gene expression profiling reveals novel biomarkers in nonsmall cell lung cancer.* Int J Cancer, 2011. **129**(2): p. 355-64.
20. Botling, J., et al., *Biomarker discovery in non-small cell lung cancer: integrating gene expression profiling, meta-analysis, and tissue microarray validation.* Clin Cancer Res, 2013. **19**(1): p. 194-204.

21. Der, S.D., et al., *Validation of a histology-independent prognostic gene signature for early-stage, non-small-cell lung cancer including stage IA patients.* J Thorac Oncol, 2014. **9**(1): p. 59-64.
22. Tarca, A.L., et al., *Strengths and limitations of microarray-based phenotype prediction: lessons learned from the IMPROVER Diagnostic Signature Challenge.* Bioinformatics, 2013. **29**(22): p. 2892-9.

Table S15. The sphingolipid/lysophosphatidate/immune signature-linked Canonical Pathways identified by IPA. The ranking is based on the corresponding IPA-based p-value; all significant results are shown ($p < 0.05$); the molecules associated with the corresponding Canonical Pathway are listed.

**Ingenuity Canonical Pathways**	**p-value**	**Molecules**
Ceramide Signaling	7.94E-17	S1PR2, S1PR4, SMPD2, SMPD3, S1PR3, SPHK2, CERK, SMPD1, SPHK1, S1PR5, S1PR1
Sphingosine-1-phosphate Signaling	1.58E-15	S1PR2, S1PR4, SMPD2, SMPD3, S1PR3, NAAA, SMPD1, ASAH1, SPHK1, S1PR5, S1PR1
Sphingomyelin Metabolism	2.00E-12	SMPD2, SMPD3, SGMS2, SMPD1, SGMS1
Sphingosine and Sphingosine-1-phosphate Metabolism	1.38E-09	SGPP1, NAAA, ASAH1, SGPP2
RhoA Signaling	2.57E-07	LPAR6, LPAR1, LPAR2, LPAR5, LPAR4, LPAR3
Gα12/13 Signaling	3.72E-07	LPAR6, LPAR1, LPAR2, LPAR5, LPAR4, LPAR3
Human Embryonic Stem Cell Pluripotency	6.46E-07	S1PR2, S1PR4, S1PR3, SPHK1, S1PR5, S1PR1
eNOS Signaling	1.05E-06	LPAR6, LPAR1, LPAR2, LPAR5, LPAR4, LPAR3
Primary Immunodeficiency Signaling	3.55E-06	CD3E, IGHM, IGHG1, PTPRC
Lipid Antigen Presentation by CD1	2.45E-05	CD3E, CD1D, CD1B
Ceramide Degradation	6.92E-05	NAAA, ASAH1
UVC-Induced MAPK Signaling	1.05E-04	SMPD2, SMPD3, SMPD1
Triacylglycerol Biosynthesis	1.20E-04	PLPP2, PLPP3, PLPP1
Hematopoiesis from Pluripotent Stem Cells	1.48E-04	CD3E, IGHM, IGHG1
UVA-Induced MAPK Signaling	1.45E-03	SMPD2, SMPD3, SMPD1
Systemic Lupus Erythematosus Signaling	1.45E-03	CD3E, IGHM, IGHG1, PTPRC
NGF Signaling	2.14E-03	SMPD2, SMPD3, SMPD1
Gαi Signaling	2.29E-03	LPAR1, S1PR3, S1PR1
B Cell Development	2.63E-03	IGHM, PTPRC
Type II Diabetes Mellitus Signaling	2.69E-03	SMPD2, SMPD3, SMPD1
Dendritic Cell Maturation	8.13E-03	CD1D, IGHG1, CD1B
cAMP-mediated signaling	1.23E-02	LPAR1, S1PR3, S1PR1
PDGF Signaling	1.66E-02	SPHK2, SPHK1
G-Protein Coupled Receptor Signaling	2.09E-02	LPAR1, S1PR3, S1PR1
T Cell Receptor Signaling	2.34E-02	CD3E, PTPRC
iCOS-iCOSL Signaling in T Helper Cells	2.88E-02	CD3E, PTPRC
Gustation Pathway	2.88E-02	LPAR6, LPAR4
CD28 Signaling in T Helper Cells	3.31E-02	CD3E, PTPRC

Table S16. The sphingolipid/lysophosphatidate/immune signature-linked Upstream Regulators identified by IPA. The ranking is based on the corresponding IPA-based p-value; all significant results are shown ($p < 0.05$); the sphingolipid/immune signature-derived molecules associated with the corresponding Upstream Regulator are listed.

Upstream Regulator	Molecule Type	p-value	Target molecules in dataset
ASAH1	enzyme	6.26E-14	CERS1,CERS2,CERS3,CERS6,SMPD1,SPHK1,SPHK2
TLR4	transmembrane receptor	2.36E-12	CD14,CD163,CERK,CERS2,CERS5,CERS6,SGMS2,SGPL1,SMPD1,SMPD2,SPHK1,UGCG
CERS2	transcription regulator	9.26E-10	ASAH1,CERS4,CERS5,CERS6
CERS4	transcription regulator	7.21E-09	ASAH1,CERS2,CERS6
TLR9	transmembrane receptor	8.00E-09	CERK,CERS6,SGMS2,SGPL1,SMPD1,SMPD2,SPHK1,UGCG
sphingosine-1-phosphate	chemical - endogenous mammalian	6.60E-07	ENPP2,S1PR1,S1PR2,S1PR3,SPHK1
CERS3	transcription regulator	3.82E-06	ASAH1,CERS2
GNPAT	enzyme	3.82E-06	UGCG,UGT8
rituximab	biologic drug	3.96E-06	IGHG1,IGHM,MS4A1
CERS5	transcription regulator	1.14E-05	ASAH1,CERS2
TNF	cytokine	1.26E-05	CD14,CD163,CD1B,CD3E,ENPP2,LPAR6,PLPP1,PTPRC,S1PR3,SMPD1,SMPD2,SPHK1,UGCG
idelalisib	chemical drug	2.28E-05	S1PR1,UGCG
Ki16425	chemical reagent	3.80E-05	S1PR3,SPHK1
SPI1	transcription regulator	5.26E-05	CD14,CD1D,CD68,MS4A1,PTPRC
lipopolysaccharide	chemical drug	6.38E-05	CD14,CD163,CD1D,CD3E,ENPP2,IGHG1,IGHM,LPAR1,S1PR1,S1PR3,SGPP2,SPHK1
CERS6	transcription regulator	1.06E-04	ASAH1,CERS5
Gm-csf	group	1.49E-04	CD14,CD163,CD1B
fingolimod	chemical drug	1.58E-04	S1PR1,S1PR3,SPHK1
LPAR1	g-protein coupled receptor	2.93E-04	S1PR3,SPHK1
IgG	complex	3.21E-04	ASAH1,MS4A1,S1PR1,UGCG
S1PR2	g-protein coupled receptor	3.94E-04	S1PR2,SPHK1
Vegf	group	4.03E-04	ENPP2,LPAR1,LPAR6,PLPP1,PLPP3,UGCG
NOS2	enzyme	4.84E-04	CD14,CD3E,IGHG1,LPAR1
fumonisin B1	chemical toxicant	6.39E-04	CD1D,UGCG
carbon tetrachloride	chemical toxicant	7.12E-04	S1PR2,S1PR3,SPHK1,SPHK2
IL4	cytokine	8.18E-04	CD14,CD163,CD1B,IGHG1,MS4A1,SPHK1,SPHK2
prostaglandin A1	chemical - endogenous non-mammalian	8.59E-04	CD14,LPAR6
IL13	cytokine	8.70E-04	CD14,CD163,CD1B,ENPP2,SPHK1
phorbol myristate acetate	chemical drug	9.89E-04	CD14,CD163,CD68,CERK,PTPRC,SMPD1,SPHK1,UGCG
KITLG	growth factor	1.04E-03	CD14,CD68,IGHM,UGCG
TGFB1	growth factor	1.10E-03	CD14,CD163,CD68,IGHM,LPAR1,LPAR4,PTPRC,S1PR3,S1PR4,SPHK1
hemocyanin	biologic drug	1.11E-03	CD14,IGHG1
LEP	growth factor	1.52E-03	ASAH1,CD14,CD68,SMPD1,SMPD2
MIR124	group	1.60E-03	PTPRC,SPHK1
cyclosporin A	biologic drug	1.64E-03	CD14,CD163,CD1D,CD68,SPHK1
tretinoin	chemical - endogenous mammalian	1.66E-03	CD14,CD1D,CD68,ENPP2,IGHM,MS4A1,PTPRC,SMPD1,SMPD3
INPP5D	phosphatase	1.71E-03	IGHG1,IGHM
cyclopamine	chemical reagent	1.94E-03	CD14,SMPD3
1-oleoyl-lysophosphatidic acid	chemical - endogenous mammalian	1.97E-03	ENPP2
FD & C Yellow no. 6	chemical toxicant	1.97E-03	CD3E
DNAJ	group	1.97E-03	ENPP2
ALDH1L1	enzyme	1.97E-03	CERS6
PTPRCAP	other	1.97E-03	PTPRC
1,2,3,6,7,8-hexachlorodibenzodioxin	chemical toxicant	1.97E-03	IGHM
IRF8	transcription regulator	2.16E-03	ASAH1,CD14,MS4A1
NCSTN	peptidase	2.30E-03	CD14,CD68
IL6	cytokine	2.38E-03	CD14,CD163,CD68,ENPP2,IGHM,PTPRC
Hsp27	group	2.56E-03	CD14,CD163
IRF6	transcription regulator	2.98E-03	CD14,ENPP2
lipid A	chemical toxicant	3.42E-03	CD14,PTPRC
Pam3-Cys	chemical toxicant	3.58E-03	CD14,CD163
docosahexaenoic acid	chemical drug	3.62E-03	SMPD1,SMPD2,SPHK1
TCF7L2	transcription regulator	3.62E-03	CERS2,ENPP2,LPAR3,UGT8
N-acetylsphingosine	chemical reagent	3.74E-03	SPHK1,UGCG
methylprednisolone	chemical drug	3.93E-03	ASAH1,ENPP2,PTPRC,SMPD3,UGCG
interferon gamma-1b	biologic drug	3.95E-03	CD163
SEW 2871	chemical reagent	3.95E-03	S1PR1
KLF14	other	3.95E-03	SPHK1
GFRA1	transmembrane receptor	3.95E-03	PTPRC
miR-574-5p (miRNAs w/seed GAGUGUG)	mature microrna	3.95E-03	CERS1
HNRNPL	other	3.95E-03	PTPRC
Ly6a (includes others)	other	3.95E-03	IGHM
RACGAP1	transporter	3.95E-03	CD14
urushiol	chemical - endogenous non-mammalian	3.95E-03	CD1D
Z-LEHD-FMK	chemical reagent	3.95E-03	SPHK1
KT 5926	chemical - kinase inhibitor	3.95E-03	CD14
FOS	transcription regulator	4.05E-03	CD14,CD68,CERS4,SPHK1,UGCG
SHC1	other	4.40E-03	S1PR1,S1PR2
POU2AF1	transcription regulator	4.40E-03	IGHG1,IGHM
RUNX3	transcription regulator	4.57E-03	SGPP1,UGCG
CSF1	cytokine	4.61E-03	CD163,CD68,PTPRC
IL21	cytokine	4.76E-03	CD1D,IGHG1,PTPRC
PRKCD	kinase	4.99E-03	CD68,SPHK1,UGCG
NKX2-1	transcription regulator	5.15E-03	CD14,ENPP2,UGT8
BIRC5	other	5.30E-03	IGHG1,IGHM
trichostatin A	chemical drug	5.32E-03	CD14,CD1D,ENPP2,SGPL1,SPHK1
E. coli serotype 0127B8 lipopolysaccharide	chemical - endogenous non-mammalian	5.91E-03	CD14,LPAR4,S1PR3
SOX12	transcription regulator	5.91E-03	CD3E
SLA	other	5.91E-03	CD3E
DL-threo-dihydrosphingosine	chemical - kinase inhibitor	5.91E-03	SPHK1
adapalene	chemical drug	5.91E-03	CD1D
lisofylline	chemical drug	5.91E-03	CERS1
progesterone	chemical - endogenous mammalian	5.93E-03	CD1D,ENPP2,PLPP3,SPHK1,UGCG
C3	peptidase	6.08E-03	IGHG1,PTPRC
Fcer1	complex	6.28E-03	S1PR2,SPHK1
TAZ	enzyme	6.28E-03	ENPP2,S1PR1
TREM1	transmembrane receptor	7.02E-03	CD14,PLPP3,S1PR3
genistein	chemical drug	7.45E-03	ASAH1,CD68,SMPD1,SPHK1
CD3E	transmembrane receptor	7.55E-03	CD3E,SPHK1
RELB	transcription regulator	7.55E-03	IGHG1,IGHM
PTPN11	phosphatase	7.77E-03	CD14,CD68
PPP3CA	phosphatase	7.77E-03	LPAR1,PLPP3
IL17A	cytokine	7.82E-03	CD14,CD163,CD68
2-(3-hydroxypropoxy)calcitriol	chemical drug	7.88E-03	S1PR2
ZXDC	transcription regulator	7.88E-03	CD14
B3GNT2	enzyme	7.88E-03	CD14
SP6	transcription regulator	7.88E-03	SPHK1
TAP1	transporter	7.88E-03	CD1D
SLA2	other	7.88E-03	CD3E
miR-515-5p (and other miRNAs w/seed UCUCCAA)	mature microrna	7.88E-03	SPHK1
lansoprazole	chemical drug	7.88E-03	CD14
procysteine	chemical drug	7.88E-03	SMPD3
Il3	cytokine	8.00E-03	CD14,CD68
tyrphostin AG 1478	chemical - kinase inhibitor	8.00E-03	S1PR3,SPHK1
SMAD4	transcription regulator	8.68E-03	LPAR1,PTPRC,S1PR4
rosiglitazone	chemical drug	9.21E-03	ASAH1,CD1D,CD68,PLPP3
forskolin	chemical toxicant	9.28E-03	CD14,CD68,CERK,PLPP1,S1PR3
TCF3	transcription regulator	9.36E-03	CD3E,CERK,IGHM
SMAD3	transcription regulator	9.47E-03	LPAR1,S1PR1,S1PR4
BTNL2	transmembrane receptor	9.66E-03	CERK,IGHM
JUN	transcription regulator	9.82E-03	CD14,CD68,LPAR1,SPHK1
W146	chemical reagent	9.84E-03	S1PR1
HLA-DR	complex	9.84E-03	IGHM
zVAD	chemical - protease inhibitor	9.84E-03	SPHK1
prednisolone	chemical drug	1.03E-02	CD163,IGHG1,SPHK2
ZBTB16	transcription regulator	1.04E-02	CD14,CD3E
ERK	group	1.08E-02	CD1D,MS4A1,UGCG
SMAD2	transcription regulator	1.17E-02	S1PR1,S1PR4
IFNG	cytokine	1.17E-02	CD14,CD163,CD1D,CERS6,IGHG1,LPAR4,S1PR3
P glycoprotein	group	1.18E-02	CD3E
Camk	complex	1.18E-02	S1PR3
CCAR1	transcription regulator	1.18E-02	SPHK1
IGLL1/IGLL5	other	1.18E-02	IGHM
CD79B	transmembrane receptor	1.18E-02	IGHM

TNFRSF10A	transmembrane receptor	1.18E-02	CD14
miR-219a-5p (and other miRNAs w/seed GAUUGUC)	mature microrna	1.18E-02	CD14
TRAT1	kinase	1.18E-02	CD3E
PSMD4	other	1.18E-02	CD14
CD99	other	1.18E-02	PTPRC
CD79A	transmembrane receptor	1.18E-02	IGHM
itraconazole	chemical drug	1.18E-02	CD14
calcium chloride	chemical drug	1.18E-02	CD1D
Z-IETD-FMK	chemical - protease inhibitor	1.18E-02	SPHK1
Z-VEID-FMK	chemical reagent	1.18E-02	SPHK1
12-hydroxyeicosatetraenoic acid	chemical - endogenous mammalian	1.18E-02	PTPRC
ALKBH5	enzyme	1.23E-02	ASAH1,SGMS1
5-O-mycolyl-beta-araf-(1->2)-5-O-mycolyl-alpha-araf-(1->1')-glycerol	chemical - endogenous non-mammalian	1.23E-02	CD1D,SPHK1
NFKB1	transcription regulator	1.25E-02	ENPP2,IGHG1,IGHM
lysophosphatidic acid	chemical - other	1.28E-02	S1PR3,SPHK1
SEMA4D	transmembrane receptor	1.37E-02	IGHG1
CD3G	transmembrane receptor	1.37E-02	CD3E
PROK1	growth factor	1.37E-02	SPHK1
FZD5	g-protein coupled receptor	1.37E-02	CD14
HP	peptidase	1.37E-02	CD163
NCR3	transmembrane receptor	1.37E-02	CD3E
zimelidine	chemical drug	1.37E-02	ASAH1
MYD88	other	1.39E-02	CD14,IGHG1,LPAR1
fingolimod phosphate	chemical - endogenous mammalian	1.57E-02	SPHK1
tectorigenin	chemical - endogenous non-mammalian	1.57E-02	LPAR1
CD8A	other	1.57E-02	IGHM
APCS	other	1.57E-02	CD163
ARF6	transporter	1.57E-02	UGCG
LY86	other	1.57E-02	CD14
ATP	chemical - endogenous mammalian	1.67E-02	CD14,SPHK1
voltage-gated calcium channel	complex	1.76E-02	PTPRC
SERPINB2	other	1.76E-02	CD14
MSH2	enzyme	1.76E-02	IGHG1
IL5	cytokine	1.80E-02	IGHG1,IGHM,PTPRC
WNT5A	cytokine	1.86E-02	CD14,ENPP2
paclitaxel	chemical drug	1.94E-02	ENPP2,IGHM,UGCG
KLRD1	transmembrane receptor	1.96E-02	CD3E
NCR1	transmembrane receptor	1.96E-02	CD3E
GBX2	transcription regulator	1.96E-02	ENPP2
terbutaline	chemical drug	1.96E-02	IGHG1
CA074-methyl ester	chemical reagent	1.96E-02	SPHK1
Z-DEVD-FMK	chemical - protease inhibitor	1.96E-02	SPHK1
ciprofloxacin	chemical drug	1.96E-02	ASAH1,CD68
prostaglandin E2	chemical - endogenous mammalian	2.01E-02	CD14,IGHG1,SPHK1
hyaluronic acid	chemical - endogenous mammalian	2.03E-02	PTPRC,SPHK1
RUNX2	transcription regulator	2.03E-02	SGPP1,UGCG
c-Src	group	2.15E-02	ASAH1
PLA2G2D	enzyme	2.15E-02	CD68
miR-208a-3p (and other miRNAs w/seed UAAGACG)	mature microrna	2.15E-02	CD14
ibrutinib	chemical drug	2.15E-02	UGCG
Igha	other	2.15E-02	IGHM
LY6E	other	2.15E-02	CD14
CACNA1A	ion channel	2.15E-02	CD68
tyrphostin AG 1296	chemical - kinase inhibitor	2.15E-02	CD14
ARNT	transcription regulator	2.21E-02	IGHM,S1PR1
IL10RA	transmembrane receptor	2.29E-02	CD1D,CERS4,S1PR1
chlorcyclizine	chemical drug	2.34E-02	ASAH1
CDK5R1	kinase	2.34E-02	CD14
LY-2510924	biologic drug	2.34E-02	CD14
N,N-dimethylsphingosine	chemical reagent	2.34E-02	SPHK1
JUNB	transcription regulator	2.46E-02	CD68,LPAR1
BCR (complex)	complex	2.54E-02	IGHG1,S1PR1
perhexiline	chemical drug	2.54E-02	ASAH1
LILRA2	other	2.54E-02	CD1B
OTX1	transcription regulator	2.54E-02	ENPP2
PON1	phosphatase	2.54E-02	CD68
INS	other	2.57E-02	SMPD1,SMPD2
APP	other	2.63E-02	CD68,ENPP2,LPAR6,PTPRC,S1PR1
IL27	cytokine	2.65E-02	CD14,CD163
dimethyl sulfoxide	chemical drug	2.65E-02	SGMS2,SMPD3
prostaglandin A2	chemical - endogenous non-mammalian	2.73E-02	LPAR6
AMG 827	biologic drug	2.73E-02	CD3E
HIRA	transcription regulator	2.73E-02	SPHK1
NHLH2	other	2.73E-02	CD68
CSF3R	transmembrane receptor	2.73E-02	CD14
SMPD1	enzyme	2.73E-02	UGCG
picryl chloride	chemical toxicant	2.73E-02	IGHG1
onapristone	chemical drug	2.73E-02	SPHK1
hydrocortisone	chemical - endogenous mammalian	2.80E-02	CD163,SMPD2
KLF3	transcription regulator	2.84E-02	CD1D,PLPP2,UGCG
BCOR	transcription regulator	2.92E-02	MS4A1
MDK	growth factor	2.92E-02	PTPRC
ITGB4	transmembrane receptor	2.92E-02	ENPP2
CR2	transmembrane receptor	2.92E-02	IGHG1
ITIH1	other	2.92E-02	SPHK1
TNFSF9	cytokine	2.92E-02	CD14
CBFA2T3	transcription regulator	2.92E-02	PTPRC
Z-551	chemical reagent	2.92E-02	CD68
IL7	cytokine	2.96E-02	IGHM,S1PR1
GC-GCR dimer	complex	3.11E-02	CD163
SATB2	transcription regulator	3.11E-02	IGHM
ABCB1	transporter	3.11E-02	UGCG
RAG1	enzyme	3.11E-02	IGHM
IL21R	transmembrane receptor	3.11E-02	IGHG1
TNFRSF13B	transmembrane receptor	3.11E-02	IGHM
PTGER4	g-protein coupled receptor	3.12E-02	LPAR6,S1PR1
IL10	cytokine	3.20E-02	CD14,CD163,CD68
Tnf (family)	group	3.21E-02	CD163,ENPP2
ACOX1	enzyme	3.29E-02	UGCG,UGT8
HNRNPA2B1	other	3.29E-02	LPAR1,S1PR3
cryptotanshinone	chemical drug	3.31E-02	CD14
LGR4	transmembrane receptor	3.31E-02	CD14
LBP	transporter	3.31E-02	CD14
IRF4	transcription regulator	3.33E-02	CD68,MS4A1
rifampin	chemical drug	3.46E-02	CD14,ENPP2
hemoglobin	complex	3.50E-02	CD163
ARHGDIG	other	3.50E-02	LPAR1
GPER1	g-protein coupled receptor	3.50E-02	ASAH1
ITGA6	transmembrane receptor	3.50E-02	ENPP2
PTK2B	kinase	3.50E-02	IGHM
cuprizone	chemical toxicant	3.50E-02	S1PR1
W7	chemical reagent	3.50E-02	CD14
thiazolidinedione	chemical drug	3.50E-02	SPHK1
heme	chemical - endogenous mammalian	3.50E-02	CD163
KLF2	transcription regulator	3.50E-02	PLPP3,S1PR1
MGEA5	enzyme	3.58E-02	CD14,CD68,PLPP3
prostaglandin E1	chemical - endogenous mammalian	3.69E-02	CD14
TRPC1	ion channel	3.69E-02	S1PR3
CD69	transmembrane receptor	3.69E-02	S1PR1
vitamin K2	chemical drug	3.69E-02	CD14
hydroxypropyl-beta-cyclodextrin	chemical drug	3.69E-02	CD68
NCF1	enzyme	3.88E-02	CD14
FGFR4	kinase	3.88E-02	LPAR1
CYP2E1	enzyme	3.88E-02	CD14
SP100	transcription regulator	3.88E-02	CD14
EPOR	transmembrane receptor	3.88E-02	IGHG1
2-mercaptoacetate	chemical - endogenous non-mammalian	3.88E-02	CD68
K-252	chemical - kinase inhibitor	3.88E-02	CD14
salvin	chemical toxicant	3.88E-02	CD14

CD40LG	cytokine	3.95E-02	CD1D,IGHG1,SPHK2
doxorubicin	chemical drug	3.95E-02	CD14,SPHK1,UGCG
RELA	transcription regulator	4.01E-02	CD14,IGHG1,IGHM
etoposide	chemical drug	4.03E-02	LPAR6,SPHK1
PTGS2	enzyme	4.03E-02	CD163,CD68
androgen	chemical drug	4.03E-02	PLPP1,PTPRC
FUT8	enzyme	4.07E-02	IGHM
IL15RA	transmembrane receptor	4.07E-02	PTPRC
TFE3	transcription regulator	4.07E-02	MS4A1
nickel	chemical toxicant	4.07E-02	UGCG
AGN194204	chemical drug	4.12E-02	CD3E,IGHM
IgG1	complex	4.26E-02	CD14
SFTPD	other	4.26E-02	CD14
RUNX1	transcription regulator	4.30E-02	SGPP1,UGCG
CSF3	cytokine	4.40E-02	CD14,ENPP2
NFAT (complex)	complex	4.45E-02	CD68
TRPS1	transcription regulator	4.45E-02	SMPD3
oxymatrine	chemical drug	4.45E-02	PTPRC
MBP	other	4.45E-02	PTPRC
desipramine	chemical drug	4.45E-02	ASAH1
lipoarabinomannan	chemical - endogenous non-mammalian	4.45E-02	CD14
raloxifene	chemical drug	4.49E-02	ENPP2,SMPD1
deoxycorticosterone acetate	chemical drug	4.64E-02	CD68
TICAM2	other	4.64E-02	IGHG1
MBD1	other	4.64E-02	IGHM
dexamethasone	chemical drug	4.74E-02	CD163,CD3E,IGHM,LPAR1,MS4A1,SPHK1,UGCG
GAS6	growth factor	4.82E-02	CD68
ELF1	transcription regulator	4.82E-02	CD1D
imipramine	chemical drug	4.82E-02	ASAH1
amitriptyline	chemical drug	4.82E-02	ASAH1

Table S17. Real-time PCR primers. Gene symbol, Gene ID and sequences of forward and reverse primers are indicated.

Gene symbol	Gene ID	Forward Primer	Reverse Primer
ASAH1	427	CAACAAAACTGTCTTCAAGGCTTC	CTGAACAGTCCTGGTTTGAATCC
CD1B	910	TCTCTTGGGCGTCCTCAATG	GGCCTCAGGCTTCACTTGTC
CD1D	912	CCTCTACTGGGGTGGGAGCTA	GAGGTAAAGCCCACAATGAGGAG
CERK	64781	TGGTTGGGTCTTGCCAGATAC	ACTTCCCACAGACGACTTGC
CERS1	10715	CTGCTGTTTGGCACCGACTA	CCGGCGTCCAGTCGTAGA
CERS2	29956	CGCCAAAGCCTCAGATCTCT	CACGTACAGCTCAAAGAAGTATCGA
CERS3	204219	GCTTTGATGTCAAGAGAAAGGATTTT	CACCAAGAGAAGCTCATCAGACTAAT
CERS4	79603	AACGGGCTTCTGATGTTGCT	CTCCATCTGGCCCTTCTTCA
CERS5	91012	TTTACAGACATTAAAAGAAAGGACTTCCT	CCCACTCGAACCATATTGTTGA
CERS6	253782	GGTCTTTGATGTTTTCTCAGTTCACT	CTCGGGCCATATTGTTGACA
ENPP2	5168	GCCTGCCGACAAGTGTGAC	GATTCGTCCTCTGAGCTATTGCA
LPAR1	1902	TGCTCACATCTTTGGCTATGTTC	AGGCCCCAAGCACAATGA
LPAR2	9170	ACACCCGCATTTTCTTCTACGT	ACGCCCCCAGGATGATG
LPAR3	23566	CCGCATACAAGTGGGTCCAT	CCACAAACGCCCCTAAGACA
LPAR4	2846	TTGCTTTTTGTCTGTACACTACCTTTTAA	CTTGCAGAGGGTGTCACCAA
LPAR5	57121	TCTGTTCTCCCGTGTCCTGACT	CAGCCAGCACCAAGCTGTAG
LPAR6	10161	CCGCCGTTTTTGTTCAGTCT	CCATGTGGCTTCTGGAAAATTT
NAAA	27163	GCTGACAGTGGATGTGCAATTC	TGGCCAGTCCATAATCCTACATAG
PPAP2A	8611	CGTTTTCCATGTACTGCATGCT	GCGTAAGAGTCTTGCCCAGTCT
PPAP2B	8613	CGTCCCGGAGAGCAAGAAC	AGGCCCGCCATGAAGAG
PPAP2C	8612	ATGGTGTTCTTGGCGCTGTAT	AGCAGCCGTGCCCACTT
S1PR1	1901	GGCTCTCCGAACGCAACTT	CAGGCTTTTTGTGTAGCTTTTCC
S1PR2	9294	GCCTCTCTACGCCAAGCATTATG	GCAGACGATAAAGACGCCTAGC
S1PR3	1903	TCTACGCACGCATCTACTTCC	TGAACCACTGAGCCTTGAAGAG
S1PR4	8698	ACCACCGACAGCTCTCTGAG	TGCTCCCCATACAGGCGAG
S1PR5	53637	CCAAAGGTGCAGTCGGAAA	CGAGGGCACAGATTTTTTGTC
SGMS1	129049	ACGCTGTACCTGTATCGGTGTATTAC	TCCCAGTCTCCGAAAAGCTTC
SGMS2	9905	CAGTGTGCTCCAAAGCTCAATG	GACAATCCACCACCAGAAATCA
SGPL1	8879	GCCAGAGAGTTTATGGTCAAGGTT	CAACTTGTCTTGAATCTTACGACCAA
SGPP1	81537	GCCGCTGGCAGTACCCT	AATAGAGTGCATTCCCATGTAAATTCT
SGPP2	130367	GGATGCATACGGTCCTGGAT	AACACAGGAAGAATGGCACAACT
SMPD1	6609	TCTATTCACCGCCATCAACCTC	CACACCTCCACCATGTCATCC
SMPD2	6610	TGAACCAGGAGAGCTTCGACC	CCACTGAACCAGTCACCATGATG
SMPD3	55512	AGGTGTTTGACAAGCGAGCAG	GGCGATGTACCCGACGATTC
SPHK1	8877	TTGAACCATTATGCTGGCTATGA	GCGGCACAGCAATAGCG
SPHK2	56848	CGTGCTTCCCATGATCTCTGA	CTGTCGTTCTGTCTGGATGAGG
UGCG	7357	GTTTGCAATGTCCACTCAAGTTG	CGTAGTTTGGTCCACCTGATCA
UGT8	7368	CCCACTACCAGAAGATCTCCAAA	CAGATACTTGACACCAGCTCCAA
CD14	929	CGGAAGACTTATCGACCATGGA	GACGCAGCGGAAATCTTCA
CD68	968	ATCCCCACCTGCTTCTCTCA	TTCTGCTGGAGGTCCTGCAT
CD163	9332	TCGTGCAATTCATCGTCTTTG	GCGAAGTTGACCACTCTCTATGC
CD3E	916	CTGGCGGCAGGCAAAGG	TTCCGGATGGGCTCATAGTCT
IGHG1*	3500	ACCCTGGTCACCGTCTCCTCAG	GTTCCACGACACCGTCACC
IGHM	3507	ACCCTGGTCACCGTCTCCTCAG	GAAGTCCTGTGCGAGGCAG
MS4A1	931	CACTCTTCAGGAGGATGTCTTCAC	GACAGCCCCCAAAGTCTTAGATT
PTPRC	5788	CCCTCAAAGATCATTTCATAATTTTACC	GTAGGCATGTAATGATAAAACATATTTCG
ACTB	60	AGGCACCAGGGCGTGAT	TGTAGAAGGTGTGGTGCCAGATT
TOP1	7150	TCCGGGCCGACATGAGT	GCCGGTGTTCTCGATCTTTG
UBC	7316	ATTTGGGTCGCAGTTCTTG	TGCCTTGACATTCTCGATGGT
YWHAZ	7534	TACTTTTGGTACATTGTGGCTTCAA	ACCTGTGACTGGAACCAATGATC

* primers detect all IgG isotypes (Mechtcheriakova, D., et al., Activation-induced cytidine deaminase (AID)-associated multigene signature to assess impact of AID in etiology of diseases with inflammatory component. PLoS One, 2011. 6(10): p. e25611.)
